# Exploring Biogeochemistry and Microbial Diversity of Extant Microbialites in Mexico and Cuba

**DOI:** 10.3389/fmicb.2018.00510

**Published:** 2018-04-03

**Authors:** Patricia M. Valdespino-Castillo, Ping Hu, Martín Merino-Ibarra, Luz M. López-Gómez, Daniel Cerqueda-García, Roberto González-De Zayas, Teresa Pi-Puig, Julio A. Lestayo, Hoi-Ying Holman, Luisa I. Falcón

**Affiliations:** ^1^Climate and Ecosystem Sciences Division, Lawrence Berkeley National Laboratory, University of California, Berkeley, Berkeley, CA, United States; ^2^Unidad Académica de Ecología y Biodiversidad Acuática, Instituto de Ciencias del Mar y Limnología, Universidad Nacional Autónoma de México, Mexico City, Mexico; ^3^Laboratorio de Ecología Bacteriana, Instituto de Ecología, Universidad Nacional Autónoma de México, Mexico City, Mexico; ^4^Centro de Investigaciones de Ecosistemas Costeros, Cayo Coco, Cuba; ^5^Instituto de Geología, Universidad Nacional Autónoma de México, Mexico City, Mexico; ^6^Laboratorio Nacional de Geoquímica y Mineralogía, Universidad Nacional Autónoma de México, Mexico City, Mexico; ^7^Molecular Biophysics and Integrated Bioimaging Division, Lawrence Berkeley National Laboratory, University of California, Berkeley, Berkeley, CA, United States

**Keywords:** mineral diversity, organic C, biomineralization, biogeochemical interactions, microbe lithification chemistry, bioactive transition elements, Mexico, Cuba

## Abstract

Microbialites are modern analogs of ancient microbial consortia that date as far back as the Archaean Eon. Microbialites have contributed to the geochemical history of our planet through their diverse metabolic capacities that mediate mineral precipitation. These mineral-forming microbial assemblages accumulate major ions, trace elements and biomass from their ambient aquatic environments; their role in the resulting chemical structure of these lithifications needs clarification. We studied the biogeochemistry and microbial structure of microbialites collected from diverse locations in Mexico and in a previously undescribed microbialite in Cuba. We examined their structure, chemistry and mineralogy at different scales using an array of nested methods including 16S rRNA gene high-throughput sequencing, elemental analysis, X-Ray fluorescence (XRF), X-Ray diffraction (XRD), Scanning Electron Microscopy-Energy Dispersive Spectroscopy (SEM-EDS), Fourier Transformed Infrared (FTIR) spectroscopy and Synchrotron Radiation-based Fourier Transformed Infrared (SR-FTIR) spectromicroscopy. The resulting data revealed high biological and chemical diversity among microbialites and specific microbe to chemical correlations. Regardless of the sampling site, Proteobacteria had the most significant correlations with biogeochemical parameters such as organic carbon (C_org_), nitrogen and C_org_:Ca ratio. Biogeochemically relevant bacterial groups (dominant phototrophs and heterotrophs) showed significant correlations with major ion composition, mineral type and transition element content, such as cadmium, cobalt, chromium, copper and nickel. Microbial-chemical relationships were discussed in reference to microbialite formation, microbial metabolic capacities and the role of transition elements as enzyme cofactors. This paper provides an analytical baseline to drive our understanding of the links between microbial diversity with the chemistry of their lithified precipitations.

## Introduction

Extant microbialites are modern analogs of stromatolite deposits left by ancient microbial consortia as early as ~3,500 Ma ago (Krumbein, 1983; Schopf, 2006). Modern microbialites comprise massive mineral structures with a growing surface layer where diverse microbial communities reside. Modern microbialites are often found in low-nutrient (oligotrophic) aquatic environments, extremely cold or hot environments (Coman et al., 2015; White et al., 2015), marine (Myshrall et al., 2010) and athalassohaline (whose ionic composition differs from that of seawater) environments (Dupraz et al., 2004; Centeno et al., 2012). The diverse metabolic capacities of microbes induce and mediate a variety of mineral precipitations (Dupraz et al., 2009) and have thereby contributed to the geochemical history of the Earth (see Des Marais, 1995, 2000; Dupraz et al., 2009).

Mineral-forming microbial assemblages accumulate biomass as well as major ions and trace elements in their growing layer. Through microbial specific metabolisms, biorelevant trace elements may be concentrated and preserved in microbialites (Webb and Kamber, 2000). In recent years, different studies have focused on the microbial communities within microbialites (microbial structure and metabolic potential) using 16S rRNA gene sequencing and metagenomics. These studies have found that microbialites harbor a highly diverse microbial community fundamentally driven by environmental factors such as pH, conductivity and availability of nitrate (Centeno et al., 2012). Genomic surveys for some microbialites have revealed a broad potential for photoautotrophy and heterotrophic pathways involved in biogeochemical C, S, N, and P cycling (Beltrán et al., 2012; Valdespino-Castillo et al., 2014, 2017; Cerqueda-García and Falcón, 2016; Saghaï et al., 2016; Alcántara-Hernández et al., 2017); and synthesis of enzyme cofactors, amino acids, production and degradation of extracellular polymeric substances (EPS) (Breitbart et al., 2009; Myshrall et al., 2010; Mobberley et al., 2013; White et al., 2015; Cerqueda-García and Falcón, 2016). Microbialite extracellular polymeric substances provide an adequate environment for binding transition metals to organic ligands (Geesey et al., 1988; Sforna et al., 2017) that may be microbe-dependent (Micheletti et al., 2008) and needs further exploration. These transition elements (Period 4 in the periodic table, from V to Zn) and heavier elements such as Cd and Mo are essential trace nutrients for organisms, present as cofactors for enzymes (i.e., Co and Ni) or structural elements in proteins (i.e., Fe and Mn) (Ledin, 2000; Rosen, 2002; Cavet et al., 2003; Silver and Phung, 2005) although some are toxic for microorganisms (Tebo and Obraztsova, 1998; Ledin, 2000; Williams and Da Silva, 2000; Silver and Phung, 2005). Besides being redox reagents, metals are used in a variety of metabolic pathways (see Webb and Kamber, 2000). Examples include: Co in cobalamin (vitamin B12) and carbonic anhydrase, Ni in [NiFe]-hydrogenase and as a cofactor in methyl-CoM reductase, Cu in thylakoidal plastocyanin and Cd in carbonic anhydrase (Ankel-Fuchs and Thauer, 1988; Lee and Morel, 1995; Butler, 1998; Williams and Da Silva, 2000; Cavet et al., 2003; Morel and Price, 2003; Giordano et al., 2005).

The chemistry and mineralogy of microbialites in relation to particular microbes needs clarification since different groups of microbes, both prokaryote and eukaryote, utilize trace elements in different ways and in different fundamental ratios (Quigg et al., 2003). It has been proposed that an accumulation of elements out of equilibrium with the surrounding environment may provide a biosignature (Webb and Kamber, 2000) of life processes. Illuminating relations between microbe type and microbialite chemistry will likely facilitate understanding of the processes that create these organo-sedimentary structures. Interdisciplinary efforts will be needed to address these questions.

Some studies, focused on the elemental chemistry of microbialites and lithifying mats have incorporated the microbial component structure and metabolic potential (see Webb and Kamber, 2011; Gérard et al., 2013; Wong et al., 2015; Paul et al., 2016; Zeyen et al., 2017) providing relevant clues to the understanding of microbialite formation and the role of microbes in geochemical signatures and mineral diversity. Here we studied microbialites collected from low nutrient aquatic environments, from four locations in Mexico and one previously undescribed microbialite in Cuba (Northern Keys, Sabinal System), using a cross-system comparison approach. Their contrasting hydro-geochemical features and the ionic composition of microbialite ambient waters are summarized in Table 1. Sampling locations include a soda lake (Lake Alchichica) and karstic (calcium carbonate) environments. Karstic locations include an inland system (PAI = Cuatro Ciénegas, México) and lagoons near the coast. Karst coastal systems characteristics include a salinity gradient from oligosaline (BAC = Bacalar and MU = Muyil, Mexico) and a hypersaline sytem (CU = Sabinal, Cuba). In order to uncover the microbial communities' compositions and their relationship with the microstructure and chemical signature of microbialites, we performed an array of nested methods including 16S rRNA gene high-throughput sequencing, XRF and elemental analysis, XRD, SEM-EDS, Fourier transform infrared (FTIR) spectroscopy and Synchrotron Radiation-based Fourier transform infrared (SR-FTIR) spectromicroscopy. We intend that this cross-system approach will be useful to explore microbial taxa relationships with chemical composition descriptors, and to gain insight on the links between microbial community structure, chemical composition, microstructure and mineral diversity.

**Table 1:** Microbialite sample locations, physicochemical and geochemical aquatic environment.

**Aquatic ecosystem**	**Temp (°C)**	**pH**	**Conductivity (mS/cm)**	**Salinity (psu)**	**Altitude (masl)**	**Major cations in water**	**Ca^+2^**	**Mg^+2^**	**Na^+^**	**K^+^**	**Cl^−^**	**${\text{SO}}_{4}^{-2}$**	**HCO3^−^**	**Mg:Ca**	**References**
							**mg/L**								
***Soda inland (athalassohaline)***															
AS, AC Alchichica lake, Mexico	18.7	9.3	13.0	7.5	2,350	Na^+^> Mg^+2^ > K^+^ > Ca^+2^	11	431	2,349	232	3,195	978	966	39.182	3, 4, 7
***Karst inland (oligosaline)***															
PAI Pozas Azules, Mexico	28.8	7.4	2.7	1.5^c^	0–700	Ca^+2^> Na^+^> Mg^+2^	385	114	165	10	121	1,441	189	0.30	1, 2
***Karst inland-coastal (oligosaline)***															
BAC Bacalar Lagoon, Mexico	29	7.8	2.2	1.2^c^-9	0–20	Ca^+2^ > Mg^+2^ > Na^+^	320	78	61	5	70	1,112	171	0.24	5
MU Muyil Lagoon, Mexico	25.5	7.7	1.7	0.7	0–20	Na^+^> Mg^+2^ > Ca^+2^ >K^+^	48	37	147	4	277	38	201	0.76	2, 6
**Karst coastal** ***(hypersaline)***															
CU Sabinal (Sabana-Camagüey System), Cuba	29	8.9	97.2	58.8	0–20	Na^+^> Mg^+2^ > K^+^ >Ca^+2^	750	2,689	21,046	807	43,330	5,529	–	3.59	This study

*^1^Johannesson et al., 2014 (mean Cuatro Cienegas); ^2^Centeno et al., 2012; ^3^Armienta et al., 2008; ^4^Caballero et al., 2003; ^5^Gischler et al., 2008; ^6^Lagomasino et al., 2015; ^7^Kaźmierczak et al., 2011; ^c^calculated from conductivity*.

## Materials and methods

### Study area

**Figure 1:**
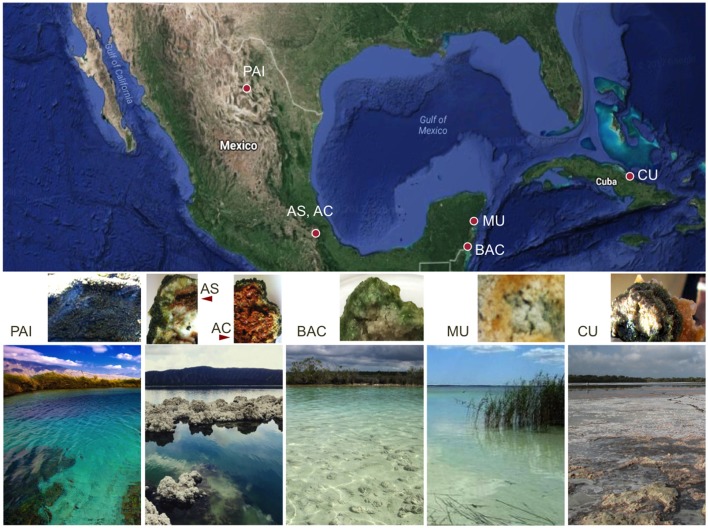
Geographical location, landscape view and cross-sections of extant microbialites. Five sampling locations include six microbialites: PAI, Pozas Azules I, Cuatro Ciénegas Basin, karstic inland, freshwater system; AS and AC, Alchichica crater lake, Mexican highlands, soda lake, athalassohaline; BAC, Bacalar Lagoon, karstic coastal, freshwater system; MU, Muyil Lagoon, Quintana Roo, karstic coastal, freshwater system; and CU, Cayo Sabinal, Northern Cuban Keys, Sabana-Camagüey System, karstic coastal, hypersaline system.

Microbialites collected for this study were sampled in five different tropical locations. The geographical location, altitude, landscape type and main water physicochemical conditions for each of the locations are described in Figure 1 and Table 1. Photographs and environmental data show that these systems are clear-water, low-nutrient (oligotrophic) environments, with characteristic ionic compositions ranging from low conductivity to hypersaline. Human activity occurs to some extent (mostly associated with tourism) near these microbialites. This study is the first report on the microbialites from Cayo Sabinal, Cuba, a hypersaline (hypersalinity >40‰; Battaglia, 1959) lagoon system in the Northern Cuban Keys (Sabana-Camagüey System; Table 1). High microbial diversity has been previously documented for some of the microbialites from Mexico. Environmental factors, such as pH, conductivity and nitrate content are relevant drivers of the microbial structure of these microbialites (Centeno et al., 2012). A metagenomic exploration over two locations of Cuatro Ciénegas Basin (same pond of our PAI microbialite) showed microbialites were enriched in genes for phosphorus metabolism, establishment and development of biofilms and heterotrophic respiration (Breitbart et al., 2009). A vast genetic diversity for nitrogen (N) and phosphorus (P) cycling has been described for the microbialites from Alchichica soda lake (Beltrán et al., 2012; Valdespino-Castillo et al., 2014, 2017; Alcántara-Hernández et al., 2017), where microbialites actively fix nitrogen (Beltrán et al., 2012). The geochemical characteristics (major cations) of microbialite ambient waters (Table 1) were compiled from different reference studies.

### Sample collection and post-treatment

Approximately 12 grams of microbialites were collected in each of five locations that include Cuatro Ciénegas, North of Mexico, Mexican highlands, Mexican Caribbean, and Cuban coast, Cayo Sabinal. All microbialites were found and collected at the surface (~less than 0.4 m depth). The general physico- and geochemical features of each sampling site are detailed in Figure 1 and Table 1. Physicochemical characterization of microbialite ambient waters included temperature, dissolved oxygen (DO) and pH. Samples used for chemical determinations (XRD, XRF, elemental analysis, SEM and DNA surveys were frozen at −20°C and sectioned in the laboratory (before drying) in order to control elemental content per area units (~1 cm^2^) and to avoid aquarium effects. Fresh samples for Infrared spectromiscroscopy were collected (1–2 weeks before the analyses) and preserved (during this period) in an aquarium with lake water in similar physicochemical conditions (DO, temperature and diel light cycle).

### Microbialite chemical characterization

#### Pulverized microbialites

Each microbialite surface sample (three per site) was divided into three fractions: (A) 5 g of each microbialite were cold-dried (10°C) for mineral and chemical composition determinations, samples were pulverized in agate mortars. Each pulverized sample was used for the next analyses: X-Ray Diffraction (XRD), X-Ray Fluorescence (XRF), elemental analysis (EA), and Fourier Transform Infrared FTIR Spectroscopy. (B) Approximately 5 g were kept frozen (−20°C) until DNA extraction and amplification and (C) approximately a cubic cm from the undisturbed microbialite surface was preserved for Scanning Electron Microscopy (SEM) coupled to an Energy-dispersive micro spectroscopy (SEM-EDS) and for SR-FTIR spectromicroscopy examinations.

#### X-ray diffraction (XRD)

For XRD analyses, samples were cold dried (10°C), ground and homogenized using a pestle and agate mortar (<75 μm) and mounted using double-side aluminum holders as non-oriented fractions. Measurements were performed in triplicates in an angular range 2θ from 5° to 70° in step scanning of 0.003° (2 Theta) and 40 s of integration time per step. Diffractograms were obtained using an EMPYREAN diffractometer equipped with a Ni filter, cooper tube and a PIXcel3D detector. The diffraction patterns were analyzed with the HighScore version 4.5 software with reference patterns from the ICDD PDF-2 and ICSD databases.

#### Elemental analysis (EA)

For organic elemental analysis, about 20 mg of the microbialite powder was used for the determination of elemental C and N (PerkinElmer 2400 Elemental Analyzer) in five replicates. Additionally, one gram subsamples of pulverized microbialite samples followed inorganic carbon removal of each sample (incubation in HCl 10%) to analyze organic carbon (C_org_) through elemental analysis; all elemental N in this fraction was assumed to be part of biomass. Total N and P were determined using this (incubated) fraction through a high temperature persulfate oxidation (Valderrama, 1981). P fractions were determined after alkaline and acid extractions as reported by López-Gómez (2003).

#### X-ray fluorescence (XRF)

Elemental composition of major elements (mg/g content in microbialites) and trace element (μg/g) was determined in triplicates by X-ray fluorescence (XRF) system (Spectro™ Xepos) under Helium atmosphere. Here pulverized microbialite samples were compressed manually with a Teflon roller prior to the measurements. In this the subsequent analysis, we organized the data into three groups: the main biogeochemical elements (C, N, P, S, Si,), the major ions in aquatic systems (Na, Mg, K, Ca, Cl), and the trace elements (i.e., Cr, As, Co, Cu, Fe, Cd, Mo, Mn, Ag, Se).

#### Fourier transform infrared (FTIR) spectroscopy and spectromicroscopy

To verify and enrich the results from XRD analysis, we also used transmission FTIR spectroscopy to characterize carbonate minerals, sulfate-bearing minerals, and silicate minerals in the microbialite powder prepared from all six microbialites. Our previous experience has shown that silicate minerals are readily detected by FTIR, but uncertain from XRD. Here, a drop of aqueous microbialite powder suspension was micropipetted onto a 1-mm thick ZnSe disc and allowed to dehydrate under a stream of dry nitrogen gas to dryness at room temperature. Normal incidence transmission spectra of the microbialite powder films and the microbialite-free ZnSe disc were recorded using a Nicolet Nic-Plan IR microscope which was coupled to a Nicolet Magna 760 FTIR bench (Thermo Scientific Inc., MA, USA), a thermal emission mid infrared light source (thermal globar) and a single-element MCT (mercury-cadmium-telluride) detector. All transmission spectra were collected in the mid-infrared region (~2.5 to ~15.5 μm wavelength, or ~4,000 to ~650 wavenumber in cm^−1^) at a spectral resolution of 4 cm^−1^ with 8 co-added scans and a peak position accuracy of 1/100 cm^−1^. All data pre- and post-processing were conducted using Thermo Electron's Omnic version 7.3. Spectral absorption peaks were compared to those in our in-house library and in published database to derive mineral information (see Table 2).

**Table 2:** Band assignments of the diagnostic vibrational modes used in FTIR spectroscopy (Figure 3) of microbialite pulverized samples.

**Minerals**	**Peak position (cm^−1^)**	**Assignment**	**References**
Carbonates	~1,780, ~1,470, ~875, ~712, ~699	Aragonite structure; coupling among ${\text{CO}}_{3}^{2-}$ groups in the presence of Ca	White, 1975; Dubrawski et al., 1989; Jones and Jackson, 1993
	~1,775, ~1,460, ~857, ~706	Aragonite structure; coupling among ${\text{CO}}_{3}^{2-}$ groups in the presence of Sr^2+^	
	~2,515, ~1,798, ~1,740, ~1,430, ~1,162, ~870, ~712	Calcite structure; coupling among ${\text{CO}}_{3}^{2-}$ groups in the presence of Ca^2+^	
	~1,480, ~1,425, ~884, ~854, ~790	Hydromagnesite structure; coupling among ${\text{CO}}_{3}^{2-}$ groups in the presence of Mg^2+^	
	~3,650, ~3,510, ~3,445	Hydromagnesite structure; coupling among O–H…O groups in the presence of Mg carbonates.	
	~1,422, ~865, ~735	Siderite structure; coupling among ${\text{CO}}_{3}^{2-}$ groups in the presence of Fe^2+^	
Silicates	~3,740, ~3,500—~3,300	Layer silicates (kaolinite) structure; O–H vibration associated with Si; replacement of Si by Al; the band broadened leads to peak broadenin	Farmer, 1975; Nash and Salisbury, 1991; Ritz et al., 2010; Müller et al., 2011; Djomgoue and Njopwouo, 2013; Kuang et al., 2016
	~1,117, ~1,100, ~1,033, ~1,011	Layer silicates (kaolinite) structure; Si–O–Si and Si–O–Al stretching vibration	
	~1,540, ~1,625	Vibration of heterocylic organic compounds H-bonded to layer silicates (kaolinite)	
	~2,970, ~2,930, ~2,875	Vibration of CH of organic compounds bonded to layer silicates (kaolinite)	
	~1,303, ~1,245, ~1,149, ~1,098, ~1,030, ~1,010	Plagioclase structure; stretching and bending vibrations of the Si–O and Al–O bonds.	
	~1,150, ~1,080, ~1,050	Quartz (α-, β-); SiO_4_ stretching and Si–O–Si bending transition.	
Sulfate-containing minerals	~1,010, ~676	Gypsum; stretch and bending vibration modes of (SO_4_)^2−^ in the presence of Ca^2+^	Ross, 1975; Lane, 2007
	~3,500, ~3,400, ~3,250	Gypsum; Combination modes of (SO_4_)^2^- and O–H (of H_2_O) vibrations in the presence of Ca^2+^	
	~1,250, ~1,124, ~676	Hexahidrite; stretch and bending vibration modes of (SO_4_)^2−^ in the presence of 6H_2_O and Mg^2+^	
Water as inclusion or structurally bonded molecules	1,640–1,620	O–H bending modes of the H_2_O molecules	Henning, 1975; Aines and Rossman, 1984; Kronenberg and Wolf, 1990
	3,600–3,000	O–H stretching vibrations of the H_2_O molecules	

To supplement information on the relative abundance and distributions of biomolecules and minerals, SR-FTIR spectromicrosopy was also performed on fresh thin layers of intact microbialites. By using a bright synchrotron as an infrared light source, this SR-FTIR approach offers a signal-to-noise ratio that is 100–1,000 times better than the thermal global FTIR approaches (Holman et al., 2010). SR-FTIR has enabled a variety of studies in biogeochemical processes (Holman et al., 1999, 2002; Baelum et al., 2012; Probst et al., 2013, 2014), in cyanobacterial silicification (Benning et al., 2004), in cyanobacteria bicarbonate transporters (Kamennaya et al., 2015), and even in microbial metabolic functions at terrestrial interface of extreme fluctuations (Holman et al., 2009, 2010). Here, intact and fresh microbialites were placed onto an infrared transparent ZnSe disc and free-flowing lake water was removed carefully without disturbing the structure of the microbialites prior to imaging. For each SR-FTIR imaging measurement, the entire view-field of the intact microbialite was divided into equal-sized 5 × 5-μm squares before scanning. Background spectra were acquired from locations without any microbialite material and were used as reference spectra. A data cube of position-associated infrared spectra was obtained following each SR-FTIR data acquisition experiment. This data cube was then subjected to an array of data processing calculations using Thermo Electron's Omnic version 7.3.

#### Scanning electron microscopy coupled to energy-dispersive detector

Intact surface (~0.1 cm^3^) microbialite dry samples were analyzed using a JEOL35C scanning electron microscope (SEM) with a dispersive X-ray spectrometer (EDS). Operating conditions were set at 15 kV accelerating voltage and 100 s measuring time.

### Nucleic acids extraction and total DNA 16S rRNA gene amplification

Approximately 2 g of each microbialite (corresponding to a cubic cm of the surface layer; in triplicates) were ground in mortars adding liquid nitrogen and a buffer solution buffer solution composed by 100 mM Tris-HCl, 20 20 mM NaCl, 100 mM EDTA (pH 8), and cetyl trimethylammonium bromide (CTAB) 0.06 of volume. Mixtures were then incubated with lysozyme (30 mg ml^−1^) (Sigma Aldrich, USA) for 30 min at 37°C. An incubation adding proteinase K (10 mg ml^−1^, Sigma Aldrich, USA) and 0.1 V of sodium dodecyl sulfate (SDS) followed (at 55°C, overnight). The aqueous phase was carefully separated (centrifuged 20 min, 1,800 g) and extracted twice with a 25:24:1 solution of phenol:chloroform:isoamyl alcohol and with 24:1 chloroform:isoamyl alcohol. DNA precipitation was conducted (at −20°C, adding 0.1 volume of sodium acetate (3M), 2 sample volumes of 2-propanol, and 2 μL of GlycoBlue (Ambion Inc., USA). Precipitated DNA was washed with ethanol twice (90–80%) and resuspended in molecular grade water. For DNA purification, Mini Spin columns (DNeasy Blood & Tissue, QIAGEN, Alameda, CA) were used following the instructions of the manufacturer. Purified DNA was stored at −20°C until analysis.

The V4 hypervariable region of prokaryote 16S rRNA gene was amplified from total DNA (in triplicates per sample site) by PCR using primers 515F/806R (Caporaso et al., 2010, 2012); PCR reactions contained a specific Golay reverse primer (Caporaso et al., 2010). Every PCR reaction plus negative controls were prepared with nuclease free-water and 2 ng/μl of total DNA per mat studied. Every PCR mix of a final volume of 25 μl contained 2.5 μl Takara ExTaq PCR 10X buffer (TaKaRa Corp., Shiga, Japan), 0.7 μl bovine serum albumin (20 mg ml-1, Roche), forward and reverse primers (10 8M final concentration), 2 μl of Takara dNTP mix (2.5 mM), and 0.625 U Takara Ex Taq DNA Polymerase. Amplification program included a (95°C, 3 min) initial denaturalization step followed by 35 cycles of 95°C (30 s) - 52°C (40 s) - 72°C (90 s); and a final (72°C, 12 min) extension. When no amplicons were detected in negative controls, three PCR products (length ~250 bp) were purified and pooled for each microbialite location (~20 ng per sample) using SPRI platform (Beckman Coulter, Brea, CA, USA). Amplicons were sequenced on the Illumina MiSeq platform (Yale Center for Genome Analysis, CT, USA). Sequences derived from this study (16S rRNA gene) are available in GenBank under BioProject PRJNA418176.

### Bioinformatic analyses

16S rRNA gene sequences were obtained in paired end reads (V4 PE reads, 2 x 250 bp), which were merged with FLASH (Magoč and Salzberg, 2011), and analyzed in the QIIME pipeline (Caporaso et al., 2010; Bokulich et al., 2013). Quality filtering and demultiplexing were performed *sensu* (Caporaso et al., 2012; Bokulich et al., 2013) using parameters *r* = 1; *p* = 0.75; *q* = 20; *n* = 0. Operational taxonomic units (OTU) clustering and chimeric sequences detection and removal were performed with USEARCH (Edgar et al., 2011) grouping sequences at 97% of similarity. Taxonomic assignation was performed using the RDP classifier (Wang et al., 2007) and Greengenes database 13.5 in QIIME 1.9. Singletons were removed (*n* = 1) and counts were normalized by rarefaction to a maximum value of 10,000 sequences. Alpha and Beta diversity analysis were performed using unifrac distance metrics (in QIIME platform) to compare the community structure and diversity of microbialite samples. We used Mantel tests based on distance dissimilarity matrices (permutations = 999) in R vegan package, and adonis (multivariate ANOVA based on dissimilarities, QIIME; permutations = 999) to determine the analysis of variance using unifrac distance matrices in order to statistically test differences in the community structure (composition and relative abundance of different taxa) with environmental parameters (microbialite chemistry). Analyses were performed with the overall community and with the most abundant bacterial groups; 75 variables were tested including Geography, Category (an indication of beta diversity clustering), biogeochemical, mineral, elemental composition and stoichiometrical ratios. Spearman tests (rho coefficient) were used to clarify significant correlations with chemical parameters at OTU level.

A taxonomic exploration of cyanobacterial phylotypes (OTUs shared in at least in four microbialites) was performed using refseq Database, NCBI. Phylogenetic affiliations are shown in Figure S2, in a phylogenetic reconstruction (GTR evolution model, Maximum likelihood, 1,000 bootstrap) that included microbialite phylotypes and their best hits.

## Results

### Chemical composition of microbialites: mineral content and major ions

**Figure 2:**
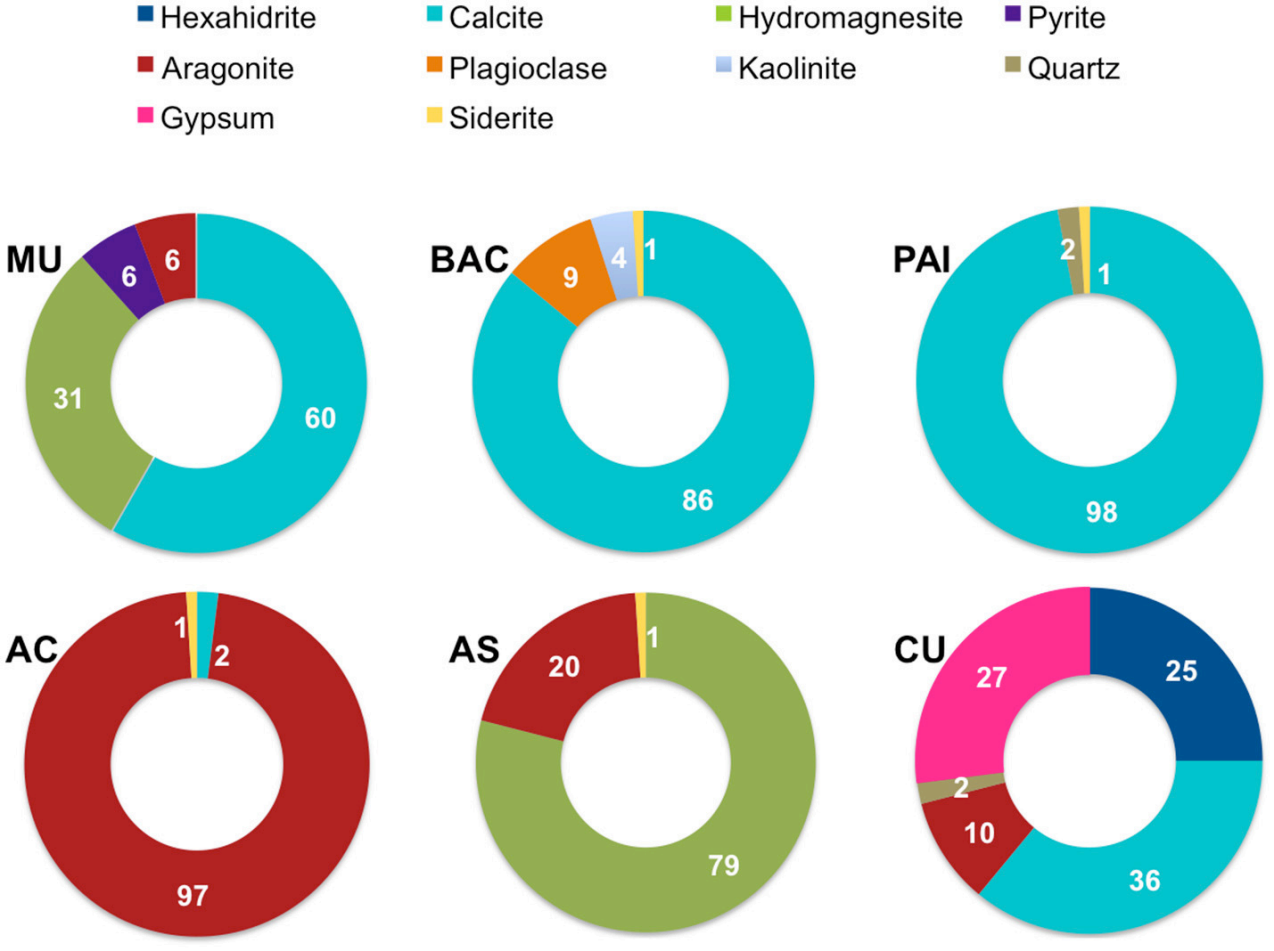
Mineral composition (as percentage) of microbialites from PAI (Pozas Azules I, Cuatro Ciénegas Basin); AS and AC (Alchichica crater lake); BAC (Bacalar Lagoon); MU (Muyil Lagoon, Quintana Roo), and CU (Cayo Sabinal, Northern Keys, Cuba).

Determinations of mineral composition by XRD [relative abundance, as percentage calculated using the Reference Intensity Method (RIR)] per site, are shown in Figure 2. XRD exam showed a total of 10 different mineral species (mineral content >1% of the bulk sample). The most abundant minerals were calcite, aragonite and hydromagnesite, these carbonates correspond to primary minerals (non diagenetically altered) *sensu* (Müller et al., 1972); iron carbonate (siderite) contributed to <1% when present. Sulfur minerals contributing >10% of microbialites were hexahidrite (hydrated magnesium sulfate), gypsum (calcium sulfate dihydrate) and pyrite (iron sulfide). Other minerals detected in low proportion by XRD were non biogenic detrital silicates such as quartz, plagioclase and kaolinite, a clay mineral derived from the weathering of alumino-silicate minerals.

**Figure 3:**
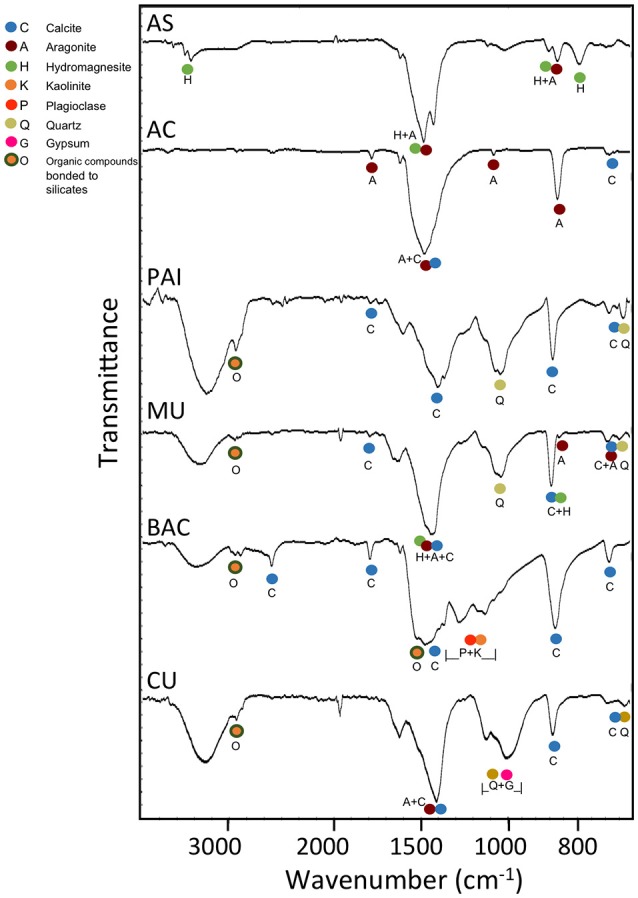
Typical FTIR transmittance spectra of pulverized microbialite samples from Alchichica soda lake (AC and AS morphotypes), Pozas Azules I (PAI, karst inland), Muyil and Bacalar (MU and BAC, karst coastal, oligosaline lagoons) in Mexico and Sabinal (CU, karst coastal, hypersaline system) in Cuba. The mineral markers are color coded for easier comparison against the FTIR band assignments of the fundamental vibrational modes in carbonate minerals (aragonite, calcite, hydromagnesite, siderite), silicate minerals (kaolinite, plagioclase, quartz), and sulfate minerals (gypsum, hexahidrite) (see Table 2). The band depths centered around the regions of 1,640–1,620 cm^−1^ and 3,600–3,000 cm^−1^ in the PAI, MU, BAC, and CU samples are from the bending and stretching vibrations of mineral water (as inclusion or structurally bonded molecules). Additional fine spectral features in the 3,000–2,850 cm^−1^ region detected are likely from the CH vibrations of organics bonded to silicate minerals.

The mid-infrared spectra of pulverized microbialite samples (Figure 3) highlight absorption peaks of the fundamental vibrational modes assigned to carbonate minerals, complex silicate minerals, and sulfate minerals. Our assignments for all peaks, as summarized in Table 2, are consistent with those in previous literature Farmer, 1975; Hu, 1980; Nash and Salisbury, 1991; Smith and Seshadri, 1999; Lane, 2007; Djomgoue and Njopwouo, 2013; Müller et al., 2014). A comparative analysis of FTIR spectra shows spectra from all six microbialites (AS, AC, PAI, MU, BAC, and CU) have strong absorption bands in the 900–700 cm^−1^ and the 1,500–1,400 cm^−1^ regions exhibited strong spectral features characteristics of carbonate minerals containing metal ions Ca^2+^, Mg^2+^, and Sr^2+^. Spectra from all but samples from AS and AC showed spectral signatures of silicates in the region of 1,030–1,150 cm^−1^ (Si-O and Al-O bonds of plagioclase structure and quartz), water inclusions or bonded water molecules in the region of 3,300–3,500 cm^−1^ (O-H vibration associated with water molecules in silicates); and signatures of organic molecules at ~1,540 cm^−1^ and ~2,900 cm^−1^ (heterocyclic organic compounds -H and -CH bonded to layer silicates). Only spectra of CU microbialites exhibit sulfate signatures typical of gypsum (~1,010, ~676 cm^−1^) and of hexahydrite (~1,250, ~1,124, stretch and bending vibration modes of sulfate in the presence of 6H_2_O and Mg^2+^)(see Table 2 and Figure 3 for details).

**Figure 4:**
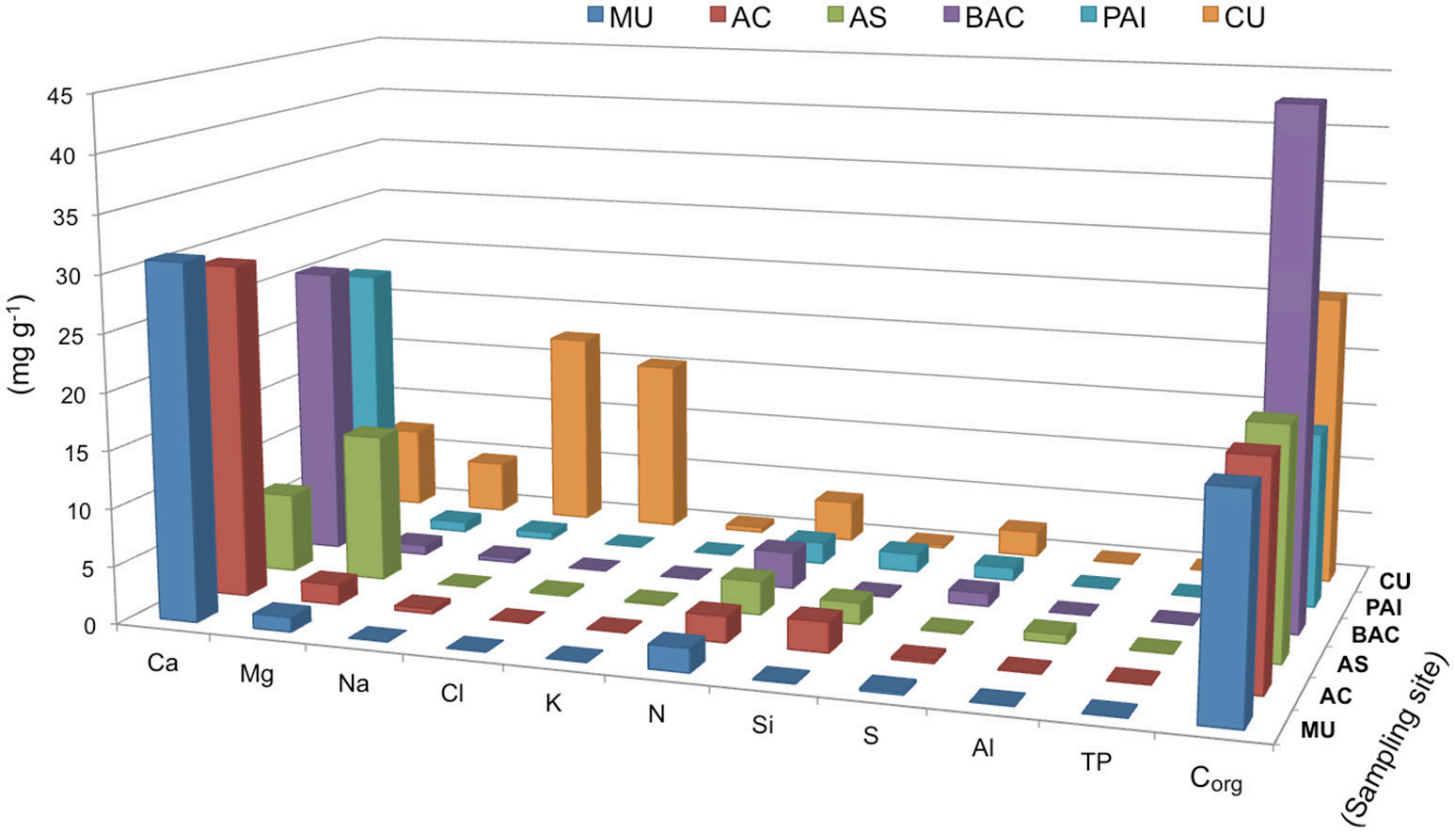
Biogeochemical parameters and major ions in six microbialites studied, bars show concentrations in mg/g of microbialites from. Parameters are organized in the x axis to allow better visualization. PAI (Pozas Azules I, Cuatro Ciénegas Basin); AS and AC (Alchichica crater lake); BAC (Bacalar Lagoon); MU (Muyil Lagoon, Quintana Roo) and CU (Cayo Sabinal, Northern Keys, Cuba).

**Figure 5:**
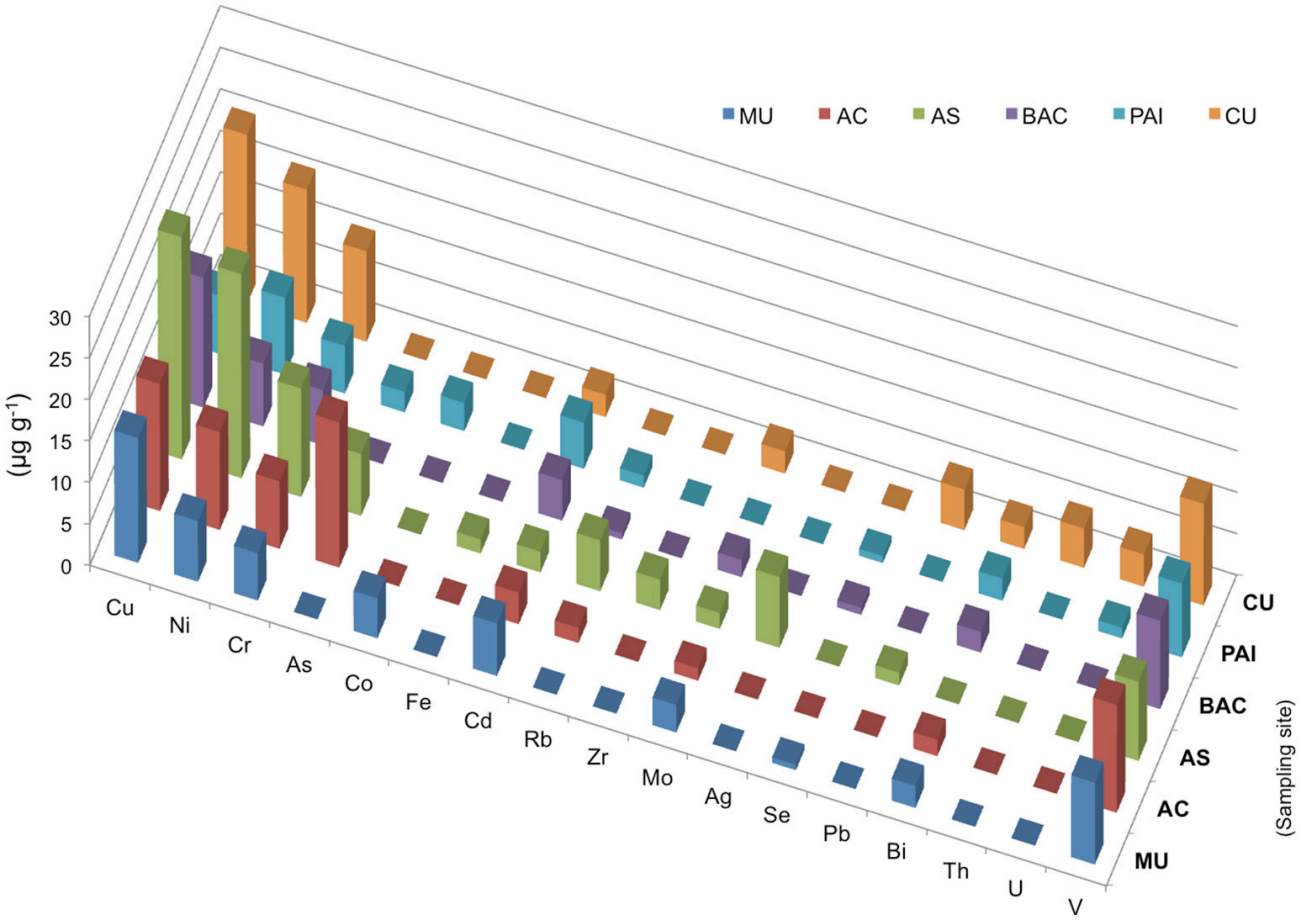
Elemental chemistry (XRF) of microbialites. Bars show concentration in μg/mg of microbialites from PAI (Pozas Azules I, Cuatro Ciénegas Basin); AS and AC (Alchichica crater lake); BAC (Bacalar Lagoon); MU (Muyil Lagoon, Quintana Roo), and CU (Cayo Sabinal, Northern Keys, Cuba).

The sequence of major ions content in the microbialites was Ca<Mg<Na in general (Figure 4). The exception was CU (coastal hypersaline system), where microbialite Na content was two orders of magnitude higher, compared to the rest of microbialites. Ca and Mg were interestingly different between the microbialite morphotypes of lake Alchichica. Mg content was the maximum in AS, the microbialite with the highest content in hydromagnesite, and contrastingly low in the Alchichica columnar morphotype (AC). Ca content showed the opposite pattern between these microbialites. Mg showed an inverse trend with aragonite among microbialites (Figure 4) and direct with arsenic, particularly for AC and AS (Figure 5). Although Mg:Ca ratio is useful to predict the type of mineral (particularly for carbonates, Müller et al., 1972), major ion ratios were not sufficient to reconstruct accurately microbialite mineral diversity or microbialite chemistry because carbonate mineral precipitation is not commonly that which would be predicted via straightforward equilibrium thermodynamic considerations, but is formed as a result of complex reaction kinetics (e.g., Morse and Casey, 1988).

### Elemental composition and main biogeochemical parameters

Main biogeochemical elements (C, N, P, S, and Si) as well as major ions (Na, Mg, K, Ca, Cl) together with aluminum contributed to concentrations in the range of mg/g in each microbialite, (Figure 4). Elemental analyses of microbialite surface samples (~1 cm^3^) showed that organic carbon concentration was similar among systems, being BAC the system with the highest C_org_ content (Figure 4). Elements (i.e., transition elements) exhibiting lower concentrations (in the range of μg/g) in the microbialites are included in Figure 5. Both, major and trace elements are more concentrated in microbialites relatively to their ambient waters. Natural systems exhibit concentration of major ions in the range of mg/L (e.g., Table 1), and trace elements in the range of μg/L (Calabrese et al., 1985). Replicates and standard deviation of microbialite chemical determinations may be consulted in Table S9.

Chromium (sensitive to aerobic manganese cycling, *sensu* Hardisty, 2016) and vanadium exhibited and elevated concentration in the microbialites studied ranging from 5.7 to 13 μg/mg overall (Figure 5). These two elements were relatively higher in the systems with higher Mg:Ca ratios and interestingly their concentration in Alchichica lake morphotypes was different and inverse. Holocene reef microbialites from Australian Great Barrier Reef also have elevated Cr and V concentrations relative to associated skeletal carbonates (Webb and Kamber, 2011), such as scleractinian -corals, mollusks and coralline red algae, and in ratios that do not reflect their abundances in seawater.

In addition to geography and nitrogen content (Table 3 and Table S5), notoriously, differences in microbialite community structure were also associated to the concentration of some metals (Table 3). Cadmium showed the strongest correlation, but the overall communities correlations to cobalt, chromium, copper and nickel also showed high scores (adonis results, Table 3). Specific microbial associations to these elements will be discussed in section The Role of Bioreactive Transition Elements Within Microbialites From Mexico and Cuba (Tables S6–S8).

**Table 3:** Adonis tests significant correlations between environmental and chemical data and overall microbial community structure (unifrac distance).

**Parameter**	**Adonis (*R*^2^)**
Category	0.3539
Cd	0.3320
N	0.3136
Geography	0.3043
Co	0.3764
Cr	0.2896
C_org_:Ca	0.2835
Cu	0.2804
N:Ca	0.2655
Ca:Mg	0.2627
Pyrite	0.2469
Calcite	0.2457
P_XRF_	0.2446
Ni	0.2380
C_org_	0.2257
C_org_:S	0.2251
N:Mg	0.1953
C_org_:Mg	0.1851

*Significant (adonis p < 0.05) are shown in red while results corresponding to (adonis > 0.05 < 0.1 are shown in blue color). Non-significant relationships (p > 0.05) with R^2^ > 0.1851 are colorless. Geography groups are karst inland, coastal and soda systems, and Category is an indication of beta diversity clustering*.

### Microbialite microstructure

SEM microscopic observations of organic “trabeculae” provided a suggestion that EPS may be contributing to C_org_ in BAC, the microbialite with the highest content of C_org._(Figure 4). Accordingly, BAC also exhibited the highest C:N and C:P and N:P ratios. Additionally, FTIR spectroscopy of BAC pulverized samples indicate the presence of organic compounds bonded to layer silicates (Figure 3, Table 2) in the mineral matrix. SR-FTIR spectromicroscopy of fresh BAC microbialite reveal the lowest transmittance (i.e., the strongest absorptions) at ~1,000 cm^−1^ that are associated with carbohydrates (Hazen et al., 2010) (**Figure 8A**). The distribution of calcite (embedded in a carbohydrates layer) spatially converges with sites rich in lipids and protein amides II (**Figure 8B**).

**Figure 6:**
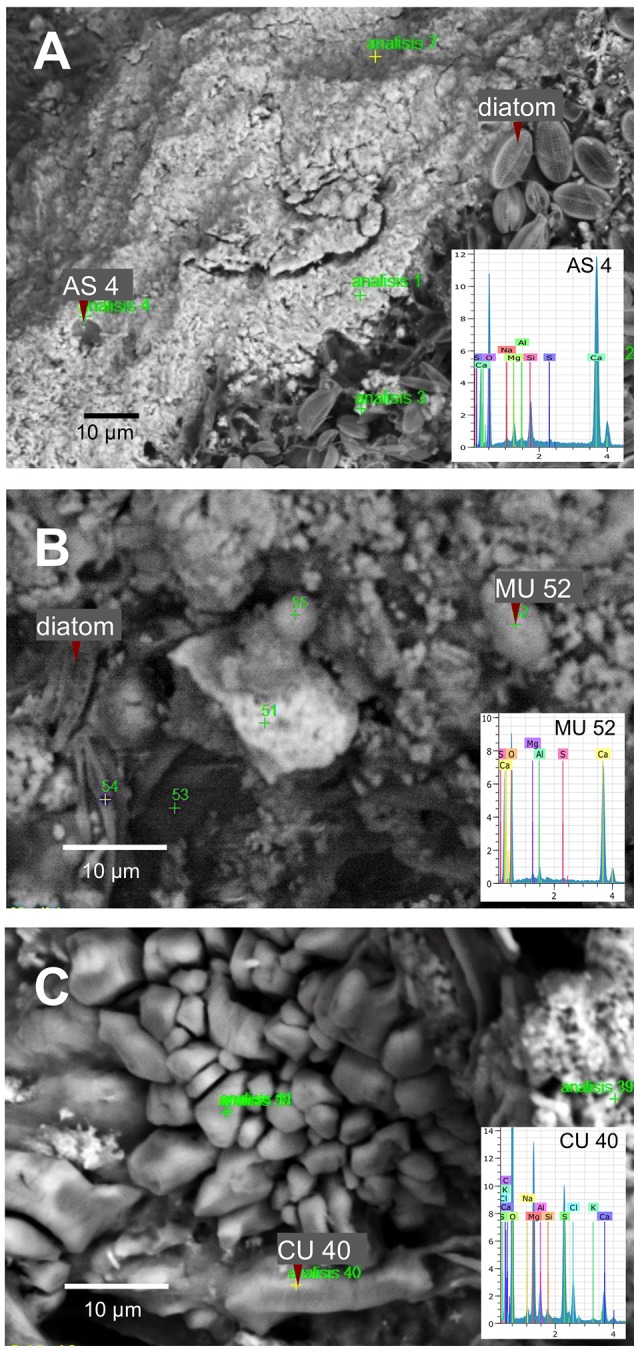
SEM-EDS spectromicroscopy exploration of microbialite surface microstructure of **(A)** AS (Alchichica spongy), **(B)** MU (Muyil), and **(C)** CU (Sabinal).

**Figure 7:**
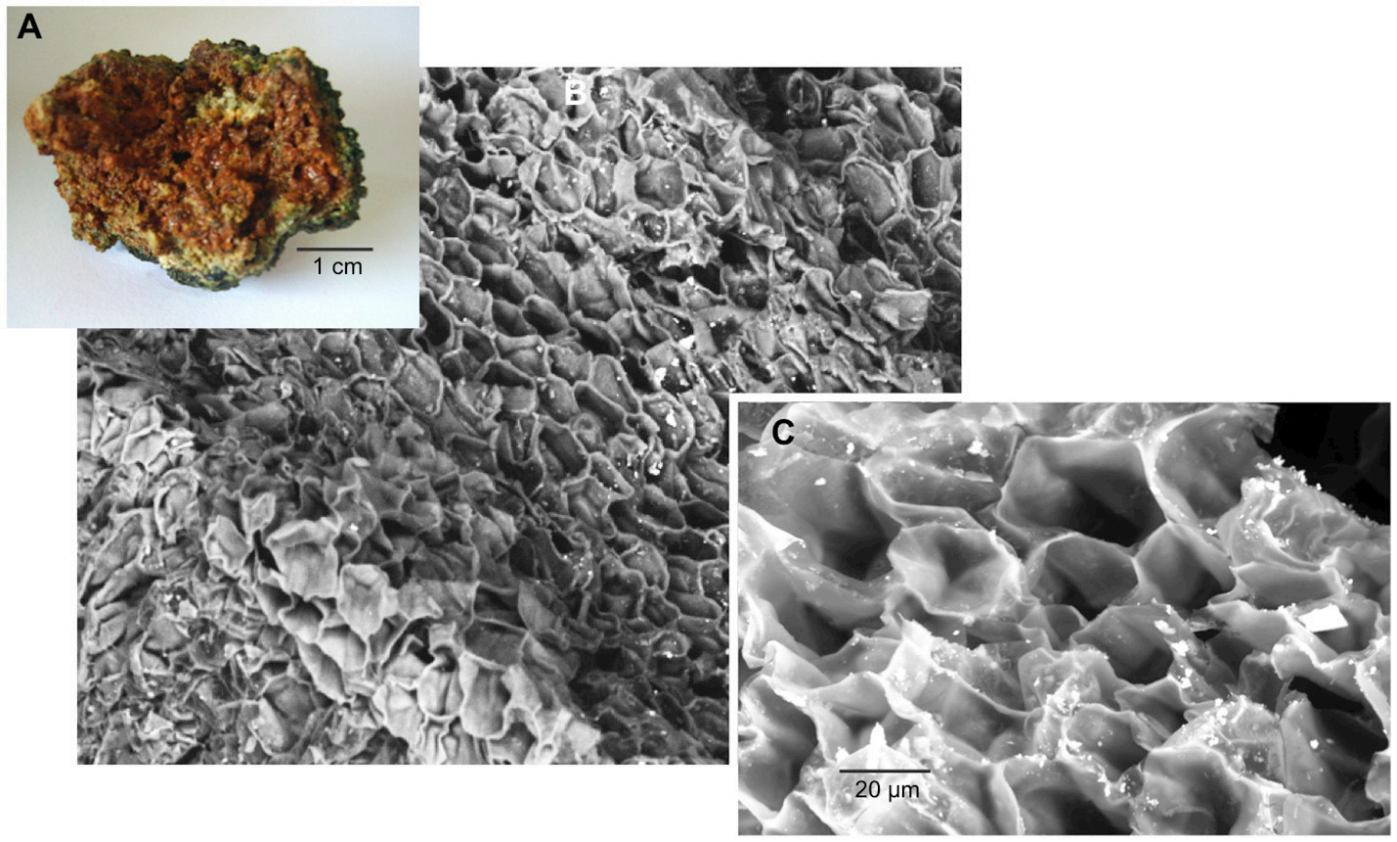
AC (Alchichica columnar) microbialite **(A)** cross section, SEM observation **(B)** 200X and **(C)** 1,000X, aragonite honey-comb shaped microstructure.

**Figure 8:**
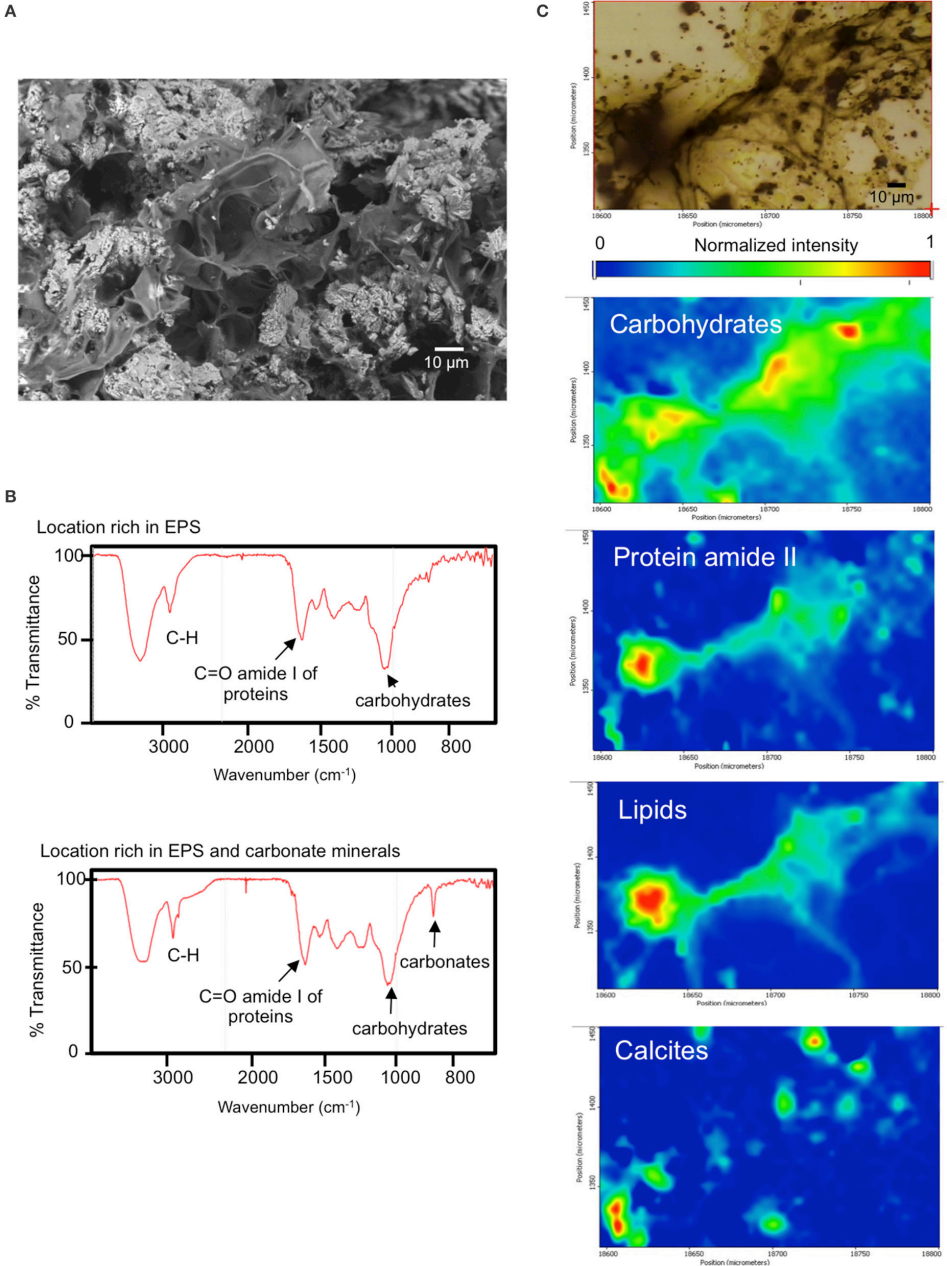
**(A)** BAC (Bacalar microbialite) mineral inclusions and embedding organic matter (top, scanning electron micrograph), **(B)** SR-FTIR spectra of surface locations rich in EPS and EPS plus minerals of fresh BAC microbialite, **(C)** SR-FTIR spectromicroscopy images (200 μm by 150 μm) showing the distribution of microbes and minerals in a living BAC microbialite. Distribution heat maps of the protein amide II vibration modes at ~1,542 cm^−1^, the carbohydrates vibration modes at ~1,000 cm^−1^, calcite at ~870 cm^−1^, and lipid is base on the CH vibration modes near 2,900 cm^−1^. Scale bars: 10 μm. Transmittance is given in % units.

SEM microscopy- EDS spectroscopy observations were useful to visualize different microbialite surface microstructures, intra and inter-site heterogeneity and the micro-features of mineral precipitations. Most of the observations showed amorphous shaped precipitations ranging in size from round (~2 μm) to tabular/laminar (up to ~20 μm) (Figures 6–8). SEM-EDS results were consistent with the results from XRD analysis, showing the presence of major cations (calcium and magnesium), Si in all microbialites, and sulfur rich-microlocations in microbialites MU and CU (Figure 6). Alchichica columnar (AC) SEM exploration showed it was the most crystalline-structured microbialite (Figure 7), a mineral matrix organized as a regular (honey bee type) network builds this mineral architecture (the main component of this, as explored by the XRD, is aragonite exhibiting pentagonal-hexagonal conducts whose diameter is ~25–30 μm). Diatoms comprised a relevant feature of the macro-architecture of microbialites including AS, MU and PAI (Figure 6). SEM-EDS analysis was useful to identify iron signals in the mineral matrix of AS.

### Microbial community structure and diversity

The total number of sequences was 1.42 million (rarefaction was performed to a depth of 10,000 per sample). Sequences clustered in 8,843 total OTUs. Four dominating microbial phyla contributed to the main differences in microbial structure. Cyanobacteria (accounting for 22–70% of abundance in Alchichica and BAC, was more abundant than in the rest of microbialites (in which they accounted for 1.6–2.5%). Proteobacteria, contributing 45–50% of abundance in the low cyanobacterial-microbialites, accounted for 7.8–33% in BAC and Alchichica microbialites (Figure S1). Firmicutes, contributing 25.5–27.6% in MU and PAI accounted for ≤0.5% in the rest of the samples. Bacteroidetes exhibited an overall variation from 3.2 to 19.6% throughout the samples. Cyanobacteria and Deltaproteobacteria distribution are shown as examples of OTU distribution among microbialites. Although Alchichica lake morphotypes share a number of cyanobacterial phylotypes, at OTU level, most of Cyanobacteria are unique for each system (Figure S1), actually only four phylotypes are shared among the five microbialite types: OTUs 818188, 164038, and 763271 (Pseudanabaenaceae) and 208315 (Phormidiaceae). A deeper taxonomic exploration of these phylotypes can be consulted in Supplementary Material (Figure S2).

Mantel tests showed that β-diversity was positively correlated with category, geography, nitrogen and cadmium (*r* = 0.632, *p* = 0.05).

### Minerals, major ions and microbes

Overall, differences in community structure were related to C_org_:Ca ratio (Table 3, Table S1), although stoichiometrical ratios N:Ca and Ca:Mg exhibited also high correlation with community structure (high adonis scores) (Table 3, Tables S2, S4). Microbial correlations with these parameters will be discussed below. Spearman test revealed that at OTU level, a number of Bacteroidetes showed positive correlation coefficients (mostly from Flavobacteriia, Cytophagia), as well as some Rhodobacterales and Burkholderiales (Alpha and Betaproteobacteria) and Pseudanabaenales (Cyanobacteria) OTUs. A number of OTUs related to these previous groups showed also strong (inverse) correlations with Ca:Mg this was supported by a high adonis *R*^2^ correlation (Table 3). Among these, Alphaproteobacteria showed a significant correlation with Ca:Mg ratio, Spearman test revealed that OTUS comprised in families Sphingomonadaceae and Rhodobacteraceae exhibited significant, inverse rho > 0.90, *p* < 0.02, Table S2). Besides their correlation to C_org_:Ca ratio, N:Ca ratio was significantly correlated to Bacteroidetes (mostly Cytophagales and Saprospirales, Alpha and Betaproteobacteria (at OTU level, Table S4). Significant correlations with mineral content (adonis *R*^2^ < 0.24, Table 3) were found in Oscillatoriales (Cyanobacteria), Xanthomonadales (Gammaproteobacteria), and Betaproteobacteria, groups that overall showed a significant correlation with pyrite. Planctomycetes was correlated with calcite and with ratios C_org_:Mg and N:Mg, in particular through OTUs assigned to the Pirellulaceae family (Table S3). The Spearman test over Oceanospirillales (Gammaproteobacteria), particularly *Halomonas* OTUs showed significant (positive rho > 0.80, *p* < 0.04) relationship.

### Specific OTU correlations with microbialite N content

**Figure 9:**
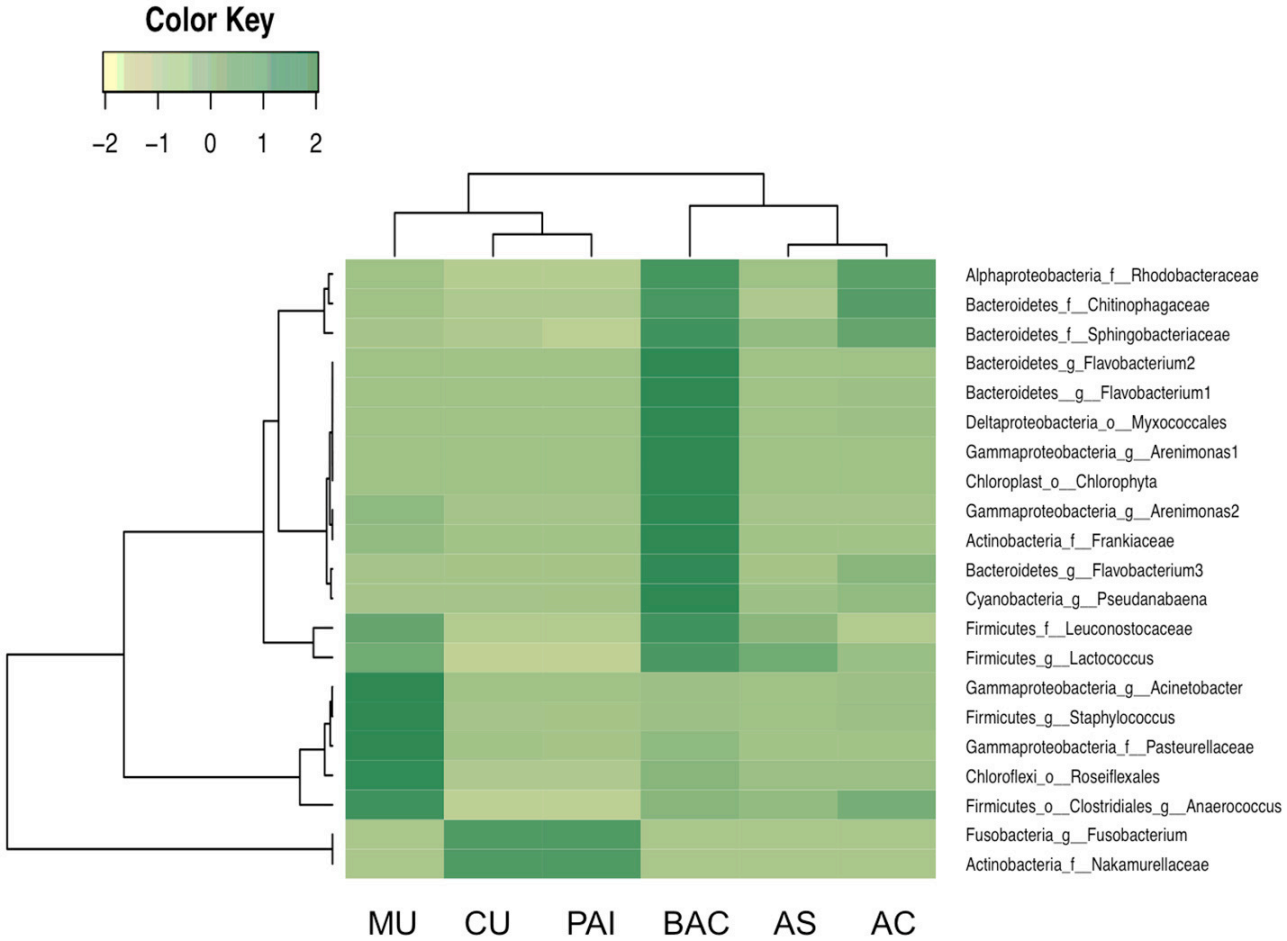
Distribution of N*OTUs, phylotypes who exhibited specific significant correlation to microbialite N content (cutoff: Spearman rho > 0.08, *p* < 0.05; shared in at least three of the six systems).

Overall microbial distribution showed a significant correlation with N (adonis *R*^2^ = 0.31358, *p* < 0.05) (Table 3). Figure 9 shows individual OTUs who exhibited a specific significant correlation to microbialite N content (cutoff: Spearman rho > 0.08, *p* < 0.05; OTUs shared in at least three of the six systems, named here N^*^OTUs). The identity of these N^*^OTUs was comprised into seven bacterial groups: Cyanobacteria, Proteobacteria (Alpha and Gamma), Bacteroidetes, Firmicutes, Actinobacteria, Chloroflexi, and Fusobacteria; the distribution was heterogeneous among systems (Figure 9). The abundance of these bacterial OTUs among the systems is shown in Figure 9. Relevant OTUs among the filtered 15 OTUs are *Pseudanabaena* (241071) and Gammaproteobacteria, Pasteurellaceae (823745), Deltaproteobacteria, Myxococcales (873518), Bacteroidetes, *Flavobacterium* (1117222), Firmicutes: Staphylococcus (1084865), *Anaerococcus* (495084). At genus level, five of the most prevalent N^*^OTUs were the Clostridiales genus *Anaerococcus* (Firmicutes) and the Bacillales *Staphylococcus* and OTU 823745 (Pasteurellaceae family, Gammaproteobacteria). Two more N^*^OTUs were the genera *Pseudanabaena* (Cyanobacteria) and OTU 1088120 from the Sphingobacteriaceae family (Bacteroidetes, Sphingobacteriales).

## Discussion

### Specific relationships among major ions, minerals and geography

The concentration of major cations including Na^+^, Ca^+2^, and K^+^ in the water environment provided a good approximation to their content in the microbialites where a higher concentration in water corresponded to higher Na^+^, Ca^+2^, and K^+^ in the microbialite, although Mg^+2^ showed a more complex pattern (see Table 1 and Figure 4), being overall higher in Alchichica AS microbialite. Our results, in general, agree with those of Müller et al. (1972) who concluded that aquatic system's Mg:Ca drives the carbonate-type formation (higher Mg:Ca ratios result on the formation of high Mg carbonates, such as hydromagnesite, and aragonite compared to low Mg calcite that forms in environmental lower Mg:Ca ratio). Intriguingly, the two microbialite morphotypes of lake Alchichica (Mg:Ca = 39) have a different mineral composition, dominated by hydromagnesite in AS and by aragonite in AC (Figures 2, 7). Since both minerals are considered primary (non-diagenetic) carbonates, we hypothesize that different particularities may be contributing to this result: i.e., (1) cation exchange among minerals (see Putnis, 2002), (2) both microbialites may have significantly different microbial communities resulting in different physiology, (3) a physicochemical process modifying the thermodynamics of mineral formation (e.g., a Mg^+2^ or Ca^+2^ local source different than the rest of the lake) or the influence of a particular physicochemical process (e.g., high energy input through waves) since AC microbialites are segregated to the area of the lake were wave-breaking occurs (see location of AS and AC in Valdespino-Castillo et al., 2014).

Besides Alchichica's microbialites, CU exhibited a relatively high content of Mg. The influence of ocean water (rich in Mg^+2^ and ${\text{SO}}_{4}^{-2}$), may be certainly contributing to this result. Among microbialites, CU (rich in Mg and NaCl) had the highest content of sulfur and one of the lowest of Ca (Figure 4). CU was the only microbialite containing gypsum (CaSO_4_·2H_2_O, hydrous calcium sulfate) and hexahidrite (MgSO_4_·6H_2_O, hydrous magnesium sulfate), the last, a mineral that has been detected in the sediments of Mars (Vaniman et al., 2004). Hydrated sulfates (i.e., bloedite, epsomite, and gypsum) have been also found in Guerrero Negro, Mexico (associated to biofilms related to gypsum precipitations; Vogel et al., 2009) and in biolaminated crusts (microbialites) living in modern magnesium sulfate lakes (Del Buey et al., 2016); here, most hydrated sulfates were associated with microbial activity since geochemical modeling was able to explain only the presence of mirabilite (Cabestrero et al., 2016). Mineral forms of the MgSO_4_.nH2O series have many hydration states; these are salts that retain a higher content of water than other cation's salts formed in extreme temperature and pressure conditions. Further studies are needed to explore if this feature may be related to life potential in extreme environments.

Besides Mg, the segregation of other elements is affected by mineral formation because carbonates are seldom pure and aragonite minerals show preferential substitution with large cations such as Sr (Milliman et al., 2012). Alchichica's microbialite morphotypes are a good example, our results showed a Sr content close to three times higher in morphotype AC compared to AS.

S and Si content in the microbialites were explained in general by the geography of the aquatic systems, which influences the microbialite geochemistry in agreement with Chagas et al. (2016), where water Si concentration and salinity exhibited strong relationships with mineral type. Si content was higher in the inland systems (microbialites AS, AC, and PAI), which have volcanic basements, than in the coastal karstic environments, various studies actually use Si as a useful tracer for ground water in karstic systems (Smith et al., 1999; Hernández-Terrones et al., 2011). Microbialite sulfur content followed the distribution of ${\text{SO}}_{4}^{-2}$ in the water (Table 1). The lowest sulfur content corresponded to microbialites from lake Alchichica and MU. Sulfur concentration (higher in the systems in proximity with marine water, such as CU) was interestingly relatively high in PAI (an inland location). It is interesting that in lithifying marine mats (i.e., in Shark Bay marine water), sulfur content is not among the most abundant elements (calcium, sodium, magnesium and potassium; Wong et al., 2015) suggesting that even in high sulfur cycling systems, sulfur is not highly accumulated; their accumulation was therefore more related to hydrated sulfates (as discussed above).

### Minerals contributing to microbialite microstructure preservation

No evident signals of diagenetic recrystallization were found for carbonates since most of the mineral composition among microbialites corresponds to primary minerals (as explained in the Results section), from recent (living) surface microbialite subsections. Silicon components of microbialites may have their origin in pre-existing substrates or from erosion processes; they were particularly abundant within BAC microbialite mineralogy (Figure 2) but were found in association with organic compounds also in PAI, MU and CU microbialites (Figure 3).

In the complex structure of surface microbialites, diatoms and some cyanobacteria (such as filamentous morphotypes) may structure cavities (crypts) that contribute to microenvironment architecture but overall, to a complex depositional environment, which includes trapped particles or shells (in the range of tens of micrometers). The growth of a microbialite, incorporating geochemical as well as biologically induced (or influenced) precipitation, requires a continual influx of ambient water into the microenvironment to provide adequate ions for mineral growth (Webb and Kamber, 2011). The surface crypts allow the formation of micro-niches (with different oxygen, light and nutrient availability) favorable for the settlement and development of average size bacteria (~2 μm); it is in these micro-sites where diverse metabolic processes mediate or influence mineral precipitations (see physicochemical models in Riding, 1991, 2000; Reid et al., 2000; Dupraz and Visscher, 2005; Dupraz et al., 2009; Martinez et al., 2016). Additionally, polymeric biogenic silica has been shown to act as a buffer for carbonic anhydrase in diatoms conducting the CO_2_ system to carbonates in the surface of diatoms (Milligan and Morel, 2002). SR-FTIRs analyses showed silicate signatures in the microbialites of PAI, MU, and BAC. In each case, signatures of heterocyclic H-bonded and CH of organic compounds bonded to layer silicates were evidenced (Figure 3, Table 2). Silicification of microbes has been studied in mats of Lake Bogoria, described as a mechanism that involves impregnation of organic biomass by amorphous silica (and silica spheroids), which contributes to the microbial microstructure preservation (Renaut et al., 1998).

### Organic carbon content, potential for oxygenic phototrophy and sulfate-reduction

BAC was the microbialite with the highest content of C_org_. Relative to the rest of microbialites, BAC showed also the highest Se content and the second highest in N and S content. Although kaolinite (Al_2_Si_2_O_5_(OH)_4_) and plagioclase (CaAl_2_Si_2_O_8_) were minerals only present in BAC, the microbialite chemistry provides no evidence of a significant accumulation of Al or Si (from detritic minerals), suggesting a higher dynamics of these elements through biotic compartments or erosive processes affecting microbialites. SEM shows BAC posses a “low” horizontally layered macrostructure compared to other microbialites. BAC phylotypes are mostly unique and the cyanobacterial community is large among microbialites (see Figure S1).

CU has the second largest C_org_ content. The abundance of sodium chloride and magnesium sulfate in a hypersaline environment (such as CU) suggests that microbes present there may be specialized to hypersaline conditions, and while halophilic archaea abundance was low, other microbial groups such as Acidobacteria distinguish CU microbial community from the rest of the microbialites studied (details are described below). All microbialite C:N, C:P, and N:P ratios were higher than Redfield ratios suggesting N and P limitation, a condition that is consistent with low nutrient concentration reported for the systems studied (Centeno et al., 2012).

Planctomycetes, particularly OTUs from Pirellulaceae family were correlated to C_org_:Mg and N:Mg ratios (Table S3). Planctomycetes are generally aerobic chemo-organoheterotrophs with complex membrane systems; their compartmentalization allows different electrochemical ion gradients linked to anammox efficiency and ATP synthesis. Their relationship with Mg may be related to the presence of volutin (or metachromatic granules) rich in phosphorus, magnesium, potassium and calcium (van Niftrik et al., 2004). Anammox microbes have the potential to assimilate ammonium without the addition of organic carbon (van Niftrik et al., 2004), therefore, Planctomycetes has been correlated to low C_org_ content and increasing C:N in soils (Hermans et al., 2017). Their direct Spearman correlations with C_org_:Mg and N:Mg in microbialites suggest the relationship of this group to low C_org_ and N sources.

Alphaproteobacterial phylotypes showed interesting relationships with major cations content. Particularly significant (and inverse) Spearman coefficients with Ca:Mg ratio found in Sphingomonadaceae and Rhodobacteraceae (Table S2), as well as an inverse correlation of OTUs to N:Ca ratio for most alphaproteobacterial families (Table S4) revealing a particular sensitivity to the proportion of these cations. Although this relationship needs further clarification; these strong correlations with major cations may suggest the relevant role of Mg in metabolic pathways (as relevant as the synthesis of bacteriochlorophyll; Boldareva-Nuianzina et al., 2013). The contrasting N:Ca correlation of OTUs, mostly positive for Bacteroidetes but negative for Proteobacteria (Table S4), may be an indication of profound differences between these groups such as in reproductive strategies, resource utilization (Taylor et al., 2013), diazotrophic potential (Alcántara-Hernández et al., 2017) and overall niche specialization. Actually, in some cases, synergistic associations have been proposed for these microbes (particularly between Flavobacterales and Rhodobacterales in phytoplankton blooms; Buchan et al., 2014).

*Halomonas* (Oceanospirillales, Gammaproteobacteria), correlated to Ca:Mg ratio, has been described as a moderately halophilic bacterial genus, mostly marine but also found in soda systems (see Valenzuela-Encinas et al., 2009) and considered part of the beneficial microbes in holobionts such as coral because of their sulfur metabolism (catabolism of dimethylsulfoniopropionate) that potentially generates sulfur-based antimicrobial compounds (Peixoto et al., 2017). Our results suggest a relationship of the halophilic condition of these bacteria, but if these bacteria mediate or bioinduce carbonates (e.g., in corals), will need further exploration.

Both oxygenic and anoxygenic photosynthetic OTUs found, indicated a broad potential for phototrophic metabolisms across the microbialite samples (Figure S1). Cyanobacteria (oxygenic photosynthetic), purple non-sulfur and purple sulfur bacteria found belong to groups Rhodobacterales, Rhodospirillales; families Acetobacteraceae, Rhodospirillaceae, Bradyrhizobiaceae, Rhodobacteraceae, Rhodobiaceae, Erythrobacteraceae (Alphaproteobacteria); families Rhodocyclaceae and Comamonadaceae (Betaproteobacteria); Chromatiaceae (Gammaproteobacteria) and chlorophototrophic bacteria (Chlorobi, Chloroflexus, and Chloracidobacteria) but no Heliobacteria (Firmicutes). Proteobacteria are dominant autotrophs across microbialite systems (Figure S1), which exhibited specific correlations to major ions (Tables S2, S4). These correlations probably suggest the marine diversification of this group, since major ions Mg^+2^, Ca^+2^, K^−^, and ${\text{SO}}_{4}^{-2}$ (and therefore Mg:Ca) of Precambrian seawater exhibited secular variations (Hardie, 1996, 2003). Species from these groups have been described to harbor carbon fixation pathways such as reductive pentose phosphate cycle, reductive citric acid cycle, reductive Acetyl-CoA pathway and 3-hydroxypropionate cycle that are likely to be present (see Canfield et al., 2005). The frequency of transcripts in oxic zones revealed that Cyanobacteria and Proteobacteria are dominant functional participants of thrombolytic mats (Mobberley et al., 2015). Further studies are needed to clarify if rhodopsin-coupled metabolic strategies are present and therefore some organisms may exhibit chlorophyll-independent photosynthetic pathways (see Bryant and Frigaard, 2006).

BAC is the microbialite with the highest content of C_org_ among samples (Figure 4). Although Cyanobacteria comprises the most abundant phototrophic composition of BAC microbialite (Figure S1), and are probably dominant in biomass, SEM and FTIR analyses revealed the contribution of EPS chemistry and organic matter bonded to silicates (Figure 3) to C_*org*_; the high content of carbohydrates to proteins in BAC EPS helped to understand the highest C:N ratio of BAC microbialite (Figure 8).

The abundance of potential sulfate reducing deltaproteobacteria (e.g., Desulfobacterales, Desulfovibrionales, Desulfuromonadales, Syntrophobacterales OTUs) accounted for less than 0.01 percent, even in the CU microbialite, abundant in ${\text{SO}}_{4}^{-}$ and NaCl, compounds required for sulfate reducing bacteria development. Consistent with this, in a metagenomic study of Alchichica's microbialites, sulfate reduction genes were also found to be negligible (Saghaï et al., 2016). Other Deltaproteobacteria such as Myxococcales, Bdellovibrionales, Spirobacillales, PB19, MIZ46 showed higher abundance. These results suggest that despite sulfate reduction has been linked to the precipitation of carbonates in modern stromatolites (Visscher et al., 1998, 2000; Reid et al., 2000; Andres et al., 2006), and to other minerals precipitation *in-vitro* (Wolicka and Borkowski, 2011) their contribution to biomineralization may be rather low in these systems, or other participants may be involved in sulfur metabolism. Acidobacteria abundance may provide a hint in this sense since Chloracidobacteria, more specifically *Chloracidobacterium thermophilum* has been used as a model to understand different pathways of sulfur metabolism including assimilatory and dissimilatory sulfate reduction and oxidation genes (cysteine and methionine metabolisms, KEGG database). The higher abundance of Acidobacteria (Chloracidobacteria Ellin6075) was a relevant feature distinguishing CU from the rest of the microbialites.

### Lactic acid bacteria (LAB) potential contribution to EPS formation and heterotrophy (fermentation)

Discarding unclassified sequences, microbial structures of MU and PAI were considerably similar (Figure S1). Both are located over karstic basement systems and showed higher abundance of Firmicutes groups (Lactobacillales, Clostridiales, and Erysipelotrichales), Gammaproteobacteria (Xanthomonadales, Oceanospirillales) and Bacteroidetes (Bacteroidales and Cytophagales). Their community structure differed clearly from the karstic systems with higher (CU) and lower (BAC) salinity. PAI and MU microbialites share some chemical features such as the highest cobalt and cadmium concentration among samples, relatively low nickel concentration (Figure 5) and the indication of organic compounds bonded to layered silicates (Figure 3); specific OTU relationships with these parameters needs clarification, although results broadly suggest the sensitivity of microbialite lactic acid bacteria (LAB) to heavy metals. Lactobacilliales have been referenced as exopolysaccharide producing LAB, their highest abundance in microbialites MU and PAI, referred by the presence of e.g., Carnobacteriaceae, genera *Dezemzia* and *Leuconostoc* (heterofermentative metabolism) points to similarities in bacterial metabolic functionality within these systems. LAB exopolysaccharides have been reported to participate in both, biofilm formation or anti-formation (Ruas-Madiedo et al., 2002). Other known LAB bacteria, such as *Bifidobacterium* (Actinobacteria) were present exclusively in these two systems. Roughly, higher fermenter LAB bacteria abundance may be an indication of high heterotrophy; in addition, C_org_ content of PAI and MU were the lowest among microbialites studied. Moreover, Firmicutes and Actinobacteria were the groups that exhibited the highest proportion of the total respiration transcripts recovered in thrombolites (Mobberley et al., 2015), together with Cyanobacteria, Alpha- and Gamma-proteobacteria. The physiological influence of fermenters (and LAB bacteria) in microbialite formation needs further exploration.

### Bacterial phylotypes related to nitrogen patterns

Different studies have shown that nitrogen availability has significant effects on microbial structural assembly (particularly bacteria; see Centeno et al., 2012; Zhao et al., 2012). While Centeno et al. (2012) showed a significant relationship of microbialite community structure and environmental nitrate, our results showed that regardless of the microbialite type (sample location), microbial phylotypes belonging to Gamma and Alphaproteobacteria (Rhodobacterales), Archaea and Acidobacteria show a significant statistical relationship with microbialite N content (Figure 9, Table 5S). Elemental C and N correlations with Cyanobacteria and Proteobacteria may be referred to their well-known machineries for (oxygenic) photosynthesis and nitrogen fixation. Interestingly, the groups with the highest correlations with organic carbon show also a high C_org_:S correlation (Synechococcales, Cyanobacteria, as well as Alpha, Beta, and Gammaproteobacteria) probably because sulfur is an abundant element in the nitrogenase architecture or because cellular sulfur (previously reduced) may be efficiently recycled during protein turnover, contributing to higher C:S ratios (see Cuhel et al., 1984).

Cyanobacterial OTUs in general showed a very heterogeneous distribution among microbialites (Figure S1), but it is interesting that three Synechococcales OTUs (two assigned to Pseudanabaenaceae) were present in most microbialites (Figure S2). *Pseudanabaena* (OTUs 241071, 225125) were the cyanobacteria that exhibited significant association to N composition (Table S5). Interestingly, *Pseudanabaena* has been observed to exhibit a particular control of nitrogen acquisition compared to other cyanobacteria. *Pseudanabaena* sp. PCC 6903 encodes only one type of glutamine synthetase (GS) type III, different to most cyanobacterial GS (type I). GS plays a major role in fixing ammonium to form glutamine and GS type III is only present in N limited environments (Crespo et al., 1998), such as the ones included in this study. *Pseudanabaena glnN* gene expression and GS type III activity showed upregulation under nitrogen starvation or using nitrate as a nitrogen source. GS catalyzes the synthesis of glutamine from glutamic acid and ammonium in the presence of divalent cations (Mg^+2^ or Mn^+2^) and using the energy of ATP hydrolysis (Muro-Pastor et al., 2005). Cyanobacterial genera *Leptolyngbya, Pseudanabaena, Acaryochloris*, and *Microcoleus* were found to be dominant photosynthetic participants of other microbialites (as in Pavilion Lake: Chan et al., 2014; Russell et al., 2014) and in tufa biofilms from karstic waters (*Pseudanabaena* and *Phormidium*: Arp et al., 2001, 2010). An exploration to insight the taxonomic resolution of the shared cyanobacterial OTUs can be consulted in Figure S2.

Other OTUs significantly related to N content comprise genera such as *Flavobacterium* (Bacteroidetes) and *Clostridium* (Firmicutes). *Flavobacterium* is one of the genera that Repert et al. (2014) found to explain differences in N-processing rates (in lake sediments). Furthermore Firmicutes and Bacteroidetes have shown a potential participation in the N cycle, harboring periplasmic pentahaem nitrite reductase (*nrfA*) genes, NADH-dependent nitrite reductase *(nirB*) in Bacilliales and in Bacteroidetes such as *Flavobacterium* (Moir, 2011). Some metalloenzymes such as copper nitrite reductase have been characterized in Flavobacteriales, who also exhibit nitric oxide reductases with homologs in Chitinophagaceae and Staphylococcus (Bacilli) (Moir, 2011).

The role of Cyanobacteria (such as *Pseudanabaena*) and Clostridiales as diazotrophs has been confirmed by the presence of nitrogenase *nifH* and more particularly, Mo-Fe-type nitrogenases in the case of Clostridiales (Moir, 2011). Also clostridial genera have been reported to harbor a NADH- dependent *nirB* type nitrite reductase. Cyanobacterial *nifH* assigned to Nostocales and Oscillatoriales cyanobacteria as well as Alpha and Gammaproteobacteria (from different microbialite locations including Alchichica, Cuatro Ciénegas and Muyil) are described in Beltrán et al. (2012) as well as Clostridiales and Deltaproteobacteria phylotypes (Alcántara-Hernández et al., 2017) in Alchichica microbialites.

### The role of bioreactive transition elements within microbialites from mexico and cuba

Interestingly, certain transition elements showed significant associations with bacterial taxa (Table 3). These are elements accumulated in microbialite precipitations (relative to their water environments), such as Co, Cu, Fe, and Ni, besides Cd and Zn (elements usually included in the transition elements group). Together these results outline a first analytic baseline in the search for the bonds between microbial diversity and the chemical environment. At taxonomic level Order, Alphaproteobacteria seems to be the group with the strongest relationships with the concentrations of transition elements; Alphaproteobacteria showed significant relations with Cd, Co, Cu, Fe, and Ni (following adonis and Spearman tests), statistical results for the whole community are shown in Table 3. Previous research following a metagenomic approach has also suggested a series of metabolic adaptations of microbialites to heavy metals (White et al., 2015; Warden et al., 2016).

#### Copper and chromium

Cu and Cr correlations with microbial community structure were high (adonis *R*^2^ > 0.28; Table 3), positive Spearman coefficients indicated a direct relationship with Alphaproteobacterial OTUs (rho > 0.8, *p* < 0.05; Table S6). Comparatively, more OTUs showed statistically significant (Spearman) relationships with Cr than with Cu. Alphaproteobacterial families Sphingomonadacea and Rhodobacteraceae (particularly *Rubellimicrobium* for Cr) grouped the OTUs more strongly and positively correlated to Cr (Table S6). The microbial response to chromium depends on the oxidation state of chromium, since Cr^(IV)^ is highly toxic while Cr^(III)^ is less toxic and bioavailable. Some Proteobacteria, Bacillales, and Clostridiales, aerobic and anaerobic, have chromate reduction abilities, acting as mediators in the reduction process of Cr^(VI)^ to Cr^(III)^, which facilitate biosorption by other organisms and therefore environmental remediation of oxidized chromium pollutants (Tandukar et al., 2009). Trivalent chromium is an essential nutrient involved in glucose utilization, lipid metabolism and possibly in the stabilization of nucleic acids (Huff et al., 1964; Mertz, 1993). Although chromium toxicity is microbe-specific, *Micrococcus, Bacillus, Pseudomona*s strains and other EPS producers exhibit remarkable high tolerance to environmental chromium. A concentration as high as 51–100 mg Cr^(VI)^ / L was reported by Srinath et al. (2002) as the minimal inhibitory concentration (at which growth doesn't occur), but some microbes have been found capable of surviving concentrations of up to 8,000 mg/L (Congeevaram et al., 2007). Chromium concentrations in the microbialites studied are lower (≤13.1 μg/g of the microbialite lithification; in the same order or magnitude if parts per million are considered) than these thresholds, but the microbialite living layer is likely to provide a locally complex (potentially more concentrated, related to their microstructure) chromium microenvironment (i.e., Cr^(VI)^ is present as dichromate in acidic environments or as chromate in alkaline environments).

Copper is an active metal for redox metabolism, it is potentially toxic and apparently carefully regulated by microbes (Prohaska, 2008). Proteobacteria harbor by themselves more than half of the proteins annotated for copper homeostasis (Protein database, NCBI) and more than 80% of the proteins associated with copper resistance. Copper homeostasis genes (e.g., copper homeostasis protein *cutC*, copper transporter *cupA*) have been recently identified in freshwater microbialites of Lake Pavilion (White et al., 2015). Alphaproteobacteria harbor 10.7 and 40% (respectively) of the total annotated bacterial *cutC* and *cupA* genes (NCBI, gene database).

### Cadmium and cobalt

Cd is a toxic element for organisms (Trevors et al., 1986) and cadmium resistance has been found in Gram-positive and Gram-negative bacteria (Trajanovska et al., 1997). Our results suggest that among metals, Cd and Co may be key elements involved in microbialite microbial composition since these heavy metals showed the strongest correlation with the distribution of different bacterial groups. Cadmium and cobalt were significantly related to the distribution of dominant microbialite organisms such as Alpha, Beta, Deltaproteobacteria, and Cyanobacteria (Oscillatoriales and Synechococcales), Bacteroidetes and Acidobacteria (overall community results are shown in Table 3 and inverse significant phylotype correlations in Table S8). These bacterial groups lead redox microbialite chemistry and metabolic pathways closely linked to mineral formation (phototrophy and sulfate reduction; see Visscher and Stolz, 2005; Mobberley et al., 2013).

Compared to Spearman Co results, more OTUs were significantly (inversely) correlated to Cd (meaning a higher number of OTUs with higher rho coefficients) strengthening the hypothesis of widespread toxicity by Cd (significant inverse Spearman coefficient). Actually, Co Spearman showed positive and inverse significant relationships with different OTUs, but the strongest correlations were inverse, including OTUs assigned to Cyanobacteria (*Synechococcales*), Bacteroidetes (Cytophagales and Flavobacteriales), Alphaproteobacteria (Rhodobacterales, Rhizobiales, and Sphingomonadales), Gammaproteobacteria (Pasteurellales, Aeromonadales, and Enterobacteriales), Firmicutes (Lactobacillales and Clostridiales) (Table S7).

Alphaproteobacteria was one of the microbial groups with the highest representation of cobaltochelatase (*cobN* genes) in thrombolites from Australia, together with Cyanobacteria, Gammaproteobacteria, Bacteroidetes and Actinobacteria (Warden et al., 2016). Consistently, in the microbialites studied here, OTUs from these groups, besides Beta- and Delta-proteobacteria showed significant relationships with Co content. Presumably *cobN* is participating in the oxygen dependent synthetic pathway of cobalamin (vitamin B12). Although vitamin B_12_ acts as a coenzyme in a wide spectrum of metabolic pathways, the actual number of known B_12_-dependent enzymes is relatively small and most organisms require cobalamin in small amounts (Raux et al., 2000). Accordingly, Spearman tests shown direct and inverse relationships within bacterial groups (e.g., Spearman coefficients were positive for many Comamonadacea, Xanthomonadaceae, and Chitinophagaceae OTUs and Beta-, Gamma-proteobacteria and Bacteroidetes families respectively), Cobalt is a relevant regulator of microbial composition among sampled microbialites from Mexico and Cuba. Saito et al. (2003) findings have been useful to explain the Cu and Co toxicity to cyanobacteria, a relevant fact probably because the concentration of metals such as Cu and Co is higher in the present biosphere compared to that of early oceans (Saito et al., 2003), overall in evaporation inland and coastal systems.

There is a broad consensus that the signatures of some transition metals remain in time, and are useful to reconstruct ancient seawater chemistry (see Riding et al., 2014). However, their signature may be disrupted by diagenetic mobilization, fractionation during secondary mineral precipitation (such as hematite and siderite), or contamination with metals derived from exogenous sources (see Petrash et al., 2016).

The concentrations of transition elements in the microbialites reported here are in the range of those reported by Petrash et al. (2016) for ancient stromatolites (chromium was marginally higher in AS and iron was overall lower.) Microbialites of this study show in general higher concentrations of trace elements compared to other microbial carbonates (Kamber and Webb, 2007).

## Conclusions

Our results revealed a high genetic and chemical (elemental and mineral) diversity among microbialites, comprising a gradient of major ions and metallic elements. Besides geography and nitrogen content, cadmium content was significantly correlated to microbial structure in the cross-system microbialite comparison. Micrometric SR-FTIR analysis showed relatively low-N polysaccharides are a major component of the EPS embedding the microbialites' surface layer. Carbonate IR signals spatially converged with nitrogen-rich (protein amide II) and lipid-rich microsites of the microbialite living layer. SR-FTIRs was essential to reveal organic compounds bonded to layer silicates in the mineral matrix, likely contributing to microbialite total organic carbon content. Cyanobacterial phylotypes differed between microbialites. Pseudanabaenaceae with metabolic abilities for life in low N environments comprised most of the cyanobacterial phylotypes shared among microbialites, and some phylotypes were significantly correlated with N content. The abundance and distribution of Synechococcales (Cyanobacteria), Rhodobacterales and Ricketsiales (Alphaproteobacteria), and Burkholderiales (Betaproteobacteria) was correlated with microbialite C_org_ content, C_org_:Ca and Ca:Mg ratios; the major cations calcium, magnesium and sodium evidently influenced both mineralogy and microbial community composition. Cyanobacteria and Planctomycetes correlated most significantly with mineral content (pyrite, calcite), C_org_:Mg and N:Mg ratios. Magnesium and calcium contents correlated with the distributions of alphaproteobacterial microbes, particularly those involved in phototrophy and N_2_ fixation. Interestingly, the dominant groups of Proteobacteria and Bacteroidetes showed the strongest correlations with trace elements, mainly Cd, Co, Cu, and Ni. These biogeochemical relationships with microbial metabolic capacities and with specific transition elements (metals) are part of the analytical baseline established here to target the search for the bonds between microbial diversity and the geochemistry of microbialites.

## Author contributions

PV-C: manuscript design. LF, H-YH, and MM-I: contributed to the drafting of the manuscript. LF, H-YH, MM-I, DC-G, LL-G, TP-P, PH, RG-DZ, and JL: contributed with analyses and expertise. all authors approved the final version. LF, H-YH, and MM-I: obtained funding for this research.

### Conflict of interest statement

The authors declare that the research was conducted in the absence of any commercial or financial relationships that could be construed as a potential conflict of interest.
