# Supplementary material for: Exploring Biogeochemistry and Microbial Diversity of Extant Microbialites in Mexico and Cuba
Source: Front Microbiol. 2018 Apr 3;9:510. doi: 10.3389/fmicb.2018.00510 (PMC5891642; doi:10.3389/fmicb.2018.00510)
Supplement: Supplementary file 1 [file DataSheet1.PDF]

Exploring Biogeochemistry And Microbial Diversity Of Extant Microbialites In Mexico And Cuba

Patricia M. Valdespino-Castillo[1], Ping Hu[1], Martín Merino-Ibarra[2], Luz María López-Gómez[2], Daniel Cerqueda-García[3], Roberto González-De Zayas[4], Teresa Pi-Puig[5], Julio A. Lestayo[4], Hoi-Ying Holman[1,7] and Luisa I. Falcón[3*]

[1] Climate and Ecosystem Sciences Division, Lawrence Berkeley National Laboratory, University of California, CA, USA.
[2] Unidad Académica de Ecología y Biodiversidad Acuática, Instituto de Ciencias del Mar y Limnología, Universidad Nacional Autónoma de México. México, D.F. México.
[3] Laboratorio de Ecología Bacteriana, Instituto de Ecología, Universidad Nacional Autónoma de México, México, D.F. México.
[4] Centro de Investigaciones de Ecosistemas Costeros. Cayo Coco, Cuba.
[5] Instituto de Geología, Universidad Nacional Autónoma de México, México, D.F. México.
[7] Molecular Biophysics and Integrated Bioimaging Division, Lawrence Berkeley National Laboratory, University of California, CA, USA.

*** Correspondence:**
Luisa I. Falcón (falcon@ecologia.unam.mx)

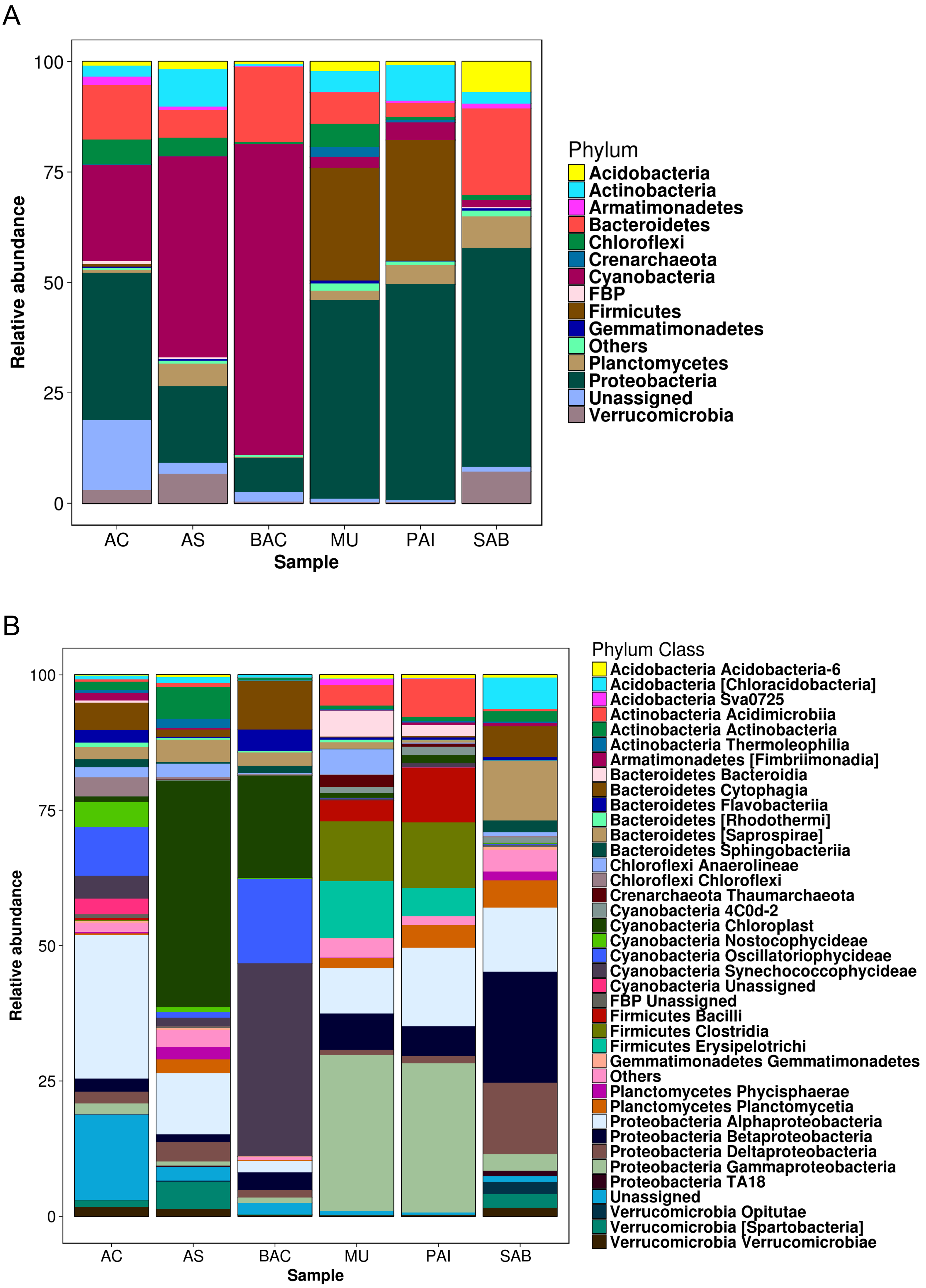

Fig. S1 Supplementary Material. OTU distribution among sampling sites. Taxonomic level A) Phylum, B) Phylum-Class. Plots show phylotypes relative abundance.

Table S1 Supplementary Material. Microbialite C_{org}:Ca ratio Spearman test (OTU level) results, cutoff= Spearman rho > 0.8, *p* <0.05

OTU ID	Spearman rho	p value	taxonomy
854272	0.9710	0.0003	k__Bacteria; p__Proteobacteria; c__Alphaproteobacteria; o__Rhodobacterales; f__Rhodobacteraceae; g__; s__
141302	0.9411	0.0025	k__Bacteria; p__Bacteroidetes; c__Flavobacteriia; o__Flavobacteriales; f__Flavobacteriaceae; g__Flavobacterium; s__
489865	0.9411	0.0025	k__Bacteria; p__Bacteroidetes; c__Flavobacteriia; o__Flavobacteriales; f__Flavobacteriaceae; g__Flavobacterium; s__
New.CleanUp.ReferenceOTU10285	0.9411	0.0025	k__Bacteria; p__Bacteroidetes; c__[Saprospirae]; o__[Saprospirales]; f__Chitinophagaceae; g__; s__
873518	0.9276	0.0045	k__Bacteria; p__Proteobacteria; c__Deltaproteobacteria; o__Myxococcales; f__; g__; s__
1117222	0.9276	0.0045	k__Bacteria; p__Bacteroidetes; c__Flavobacteriia; o__Flavobacteriales; f__Flavobacteriaceae; g__Flavobacterium; s__
1088120	0.8857	0.0152	k__Bacteria; p__Bacteroidetes; c__Sphingobacteriia; o__Sphingobacteriales; f__Sphingobacteriaceae; g__; s__
241071	0.8857	0.0152	k__Bacteria; p__Cyanobacteria; c__Synechococcophycideae; o__Pseudanabaenales; f__Pseudanabaenaceae; g__Pseudanabaena; s__
582257	-0.8804	0.0170	k__Bacteria; p__Chloroflexi; c__Anaerolineae; o__Caldilineales; f__Caldilineaceae; g__; s__
150955	0.8697	0.0211	k__Bacteria; p__Bacteroidetes; c__Cytophagia; o__Cytophagales; f__Cytophagaceae; g__Hymenobacter; s__
New.CleanUp.ReferenceOTU15689	0.8697	0.0211	k__Bacteria; p__Bacteroidetes; c__Flavobacteriia; o__Flavobacteriales; f__Flavobacteriaceae; g__Flavobacterium; s__
4396446	0.8452	0.0319	k__Bacteria; p__[Thermi]; c__Deinococci; o__Deinococcales; f__Deinococcaceae; g__Deinococcus; s__
331473	0.8452	0.0319	k__Bacteria; p__Bacteroidetes; c__Cytophagia; o__Cytophagales; f__Cytophagaceae; g__Hymenobacter; s__
1116759	0.8452	0.0319	k__Bacteria; p__Bacteroidetes; c__Flavobacteriia; o__Flavobacteriales; f__Flavobacteriaceae; g__Flavobacterium; s__
735569	0.8452	0.0319	k__Bacteria; p__Bacteroidetes; c__[Saprospirae]; o__[Saprospirales]; f__Chitinophagaceae; g__; s__
541746	0.8452	0.0319	k__Bacteria; p__Bacteroidetes; c__Cytophagia; o__Cytophagales; f__Cytophagaceae; g__Hymenobacter; s__
4410283	0.8452	0.0319	k__Bacteria; p__Bacteroidetes; c__Cytophagia; o__Cytophagales; f__Cytophagaceae; g__Hymenobacter; s__
1116398	0.8452	0.0319	k__Bacteria; p__Bacteroidetes; c__Flavobacteriia; o__Flavobacteriales; f__Flavobacteriaceae; g__Flavobacterium; s__
73825	0.8452	0.0319	k__Bacteria; p__Bacteroidetes; c__Flavobacteriia; o__Flavobacteriales; f__Flavobacteriaceae; g__Flavobacterium; s__
952130	0.8452	0.0319	k__Bacteria; p__Verrucomicrobia; c__[Spartobacteria]; o__[Chthoniobacterales]; f__[Chthoniobacteraceae]; g__; s__
719030	0.8452	0.0319	k__Bacteria; p__Bacteroidetes; c__Cytophagia; o__Cytophagales; f__Cytophagaceae; g__Hymenobacter; s__
1081614	0.8452	0.0319	k__Bacteria; p__Bacteroidetes; c__Flavobacteriia; o__Flavobacteriales; f__Flavobacteriaceae; g__Flavobacterium; s__
570086	0.8452	0.0319	k__Bacteria; p__Bacteroidetes; c__Flavobacteriia; o__Flavobacteriales; f__Cryomorphaceae; g__Fluviicola; s__
4438613	0.8452	0.0319	k__Bacteria; p__Proteobacteria; c__Betaproteobacteria; o__Burkholderiales; f__Comamonadaceae; g__; s__
994982	0.8452	0.0319	k__Bacteria; p__Bacteroidetes; c__Flavobacteriia; o__Flavobacteriales; f__Flavobacteriaceae; g__Flavobacterium; s__
1146920	0.8452	0.0319	k__Bacteria; p__Bacteroidetes; c__Cytophagia; o__Cytophagales; f__Cytophagaceae; g__Hymenobacter; s__
988172	0.8452	0.0319	k__Bacteria; p__Bacteroidetes; c__Cytophagia; o__Cytophagales; f__Cytophagaceae; g__Hymenobacter; s__
827674	0.8452	0.0319	k__Bacteria; p__Bacteroidetes; c__Cytophagia; o__Cytophagales; f__Cytophagaceae; g__Hymenobacter; s__
574686	0.8452	0.0319	k__Bacteria; p__Bacteroidetes; c__Flavobacteriia; o__Flavobacteriales; f__Flavobacteriaceae; g__Flavobacterium; s__
3261	0.8452	0.0319	k__Bacteria; p__Cyanobacteria; c__Synechococcophycideae; o__Synechococcales; f__Chamaesiphonaceae; g__; s__
4410974	0.8452	0.0319	k__Bacteria; p__Proteobacteria; c__Betaproteobacteria; o__Burkholderiales; f__Comamonadaceae; g__Polaromonas; s__
812154	0.8452	0.0319	k__Bacteria; p__Cyanobacteria; c__; o__; f__; g__; s__
4319059	0.8452	0.0319	k__Bacteria; p__Proteobacteria; c__Alphaproteobacteria; o__Sphingomonadales; f__; g__; s__
New.ReferenceOTU75	0.8452	0.0319	k__Bacteria; p__Bacteroidetes; c__Cytophagia; o__Cytophagales; f__Cytophagaceae; g__Adhaeribacter; s__
New.CleanUp.ReferenceOTU17	0.8452	0.0319	k__Bacteria; p__Bacteroidetes; c__Cytophagia; o__Cytophagales; f__Cytophagaceae; g__Adhaeribacter; s__
New.CleanUp.ReferenceOTU202	0.8452	0.0319	k__Bacteria; p__Bacteroidetes; c__Sphingobacteriia; o__Sphingobacteriales; f__; g__; s__
New.CleanUp.ReferenceOTU1808	0.8452	0.0319	k__Bacteria; p__Bacteroidetes; c__Cytophagia; o__Cytophagales; f__Cytophagaceae; g__Hymenobacter; s__
New.CleanUp.ReferenceOTU2042	0.8452	0.0319	k__Bacteria; p__Bacteroidetes; c__Cytophagia; o__Cytophagales; f__Cytophagaceae; g__Hymenobacter; s__
New.CleanUp.ReferenceOTU3674	0.8452	0.0319	k__Bacteria; p__Cyanobacteria; c__Synechococcophycideae; o__Pseudanabaenales; f__Pseudanabaenaceae; g__Pseudanabaena; s__
New.CleanUp.ReferenceOTU3819	0.8452	0.0319	k__Bacteria; p__Bacteroidetes; c__Flavobacteriia; o__Flavobacteriales; f__Flavobacteriaceae; g__Flavobacterium; s__
New.CleanUp.ReferenceOTU4614	0.8452	0.0319	k__Bacteria; p__[Thermi]; c__Deinococci; o__Deinococcales; f__Deinococcaceae; g__Deinococcus; s__aquatilis
New.CleanUp.ReferenceOTU4947	0.8452	0.0319	k__Bacteria; p__Cyanobacteria; c__Oscillatoriophycideae; o__Oscillatoriales; f__Phormidiaceae; g__Phormidium; s__
New.CleanUp.ReferenceOTU6700	0.8452	0.0319	k__Bacteria; p__Armatimonadetes; c__[Fimbriimonadia]; o__[Fimbriimonadales]; f__[Fimbriimonadaceae]; g__Fimbriimonas; s__
New.CleanUp.ReferenceOTU7270	0.8452	0.0319	k__Bacteria; p__Proteobacteria; c__Alphaproteobacteria; o__Rhodobacterales; f__Rhodobacteraceae; g__Rhodobacter; s__
New.CleanUp.ReferenceOTU7576	0.8452	0.0319	k__Bacteria; p__Bacteroidetes; c__Cytophagia; o__Cytophagales; f__Cytophagaceae; g__Hymenobacter; s__
New.CleanUp.ReferenceOTU9696	0.8452	0.0319	k__Bacteria; p__Cyanobacteria; c__Synechococcophycideae; o__Pseudanabaenales; f__Pseudanabaenaceae; g__Leptolyngbya; s__
New.CleanUp.ReferenceOTU10418	0.8452	0.0319	k__Bacteria; p__Bacteroidetes; c__Cytophagia; o__Cytophagales; f__Cytophagaceae; g__Adhaeribacter; s__
New.CleanUp.ReferenceOTU11746	0.8452	0.0319	k__Bacteria; p__Cyanobacteria; c__Chloroplast; o__Stramenopiles; f__; g__; s__
New.CleanUp.ReferenceOTU12067	0.8452	0.0319	k__Bacteria; p__Cyanobacteria; c__Synechococcophycideae; o__Pseudanabaenales; f__Pseudanabaenaceae; g__Leptolyngbya; s__
New.CleanUp.ReferenceOTU12137	0.8452	0.0319	k__Bacteria; p__Bacteroidetes; c__Cytophagia; o__Cytophagales; f__Cytophagaceae; g__Hymenobacter; s__
New.CleanUp.ReferenceOTU12435	0.8452	0.0319	k__Bacteria; p__Bacteroidetes; c__Cytophagia; o__Cytophagales; f__Cytophagaceae; g__Hymenobacter; s__
New.CleanUp.ReferenceOTU13117	0.8452	0.0319	k__Bacteria; p__Bacteroidetes; c__[Saprospirae]; o__[Saprospirales]; f__Chitinophagaceae; g__; s__
New.CleanUp.ReferenceOTU13692	0.8452	0.0319	k__Bacteria; p__Bacteroidetes; c__Flavobacteriia; o__Flavobacteriales; f__Cryomorphaceae; g__Fluviicola; s__
New.CleanUp.ReferenceOTU14548	0.8452	0.0319	k__Bacteria; p__Cyanobacteria; c__Oscillatoriophycideae; o__Oscillatoriales; f__Phormidiaceae; g__Phormidium; s__
New.CleanUp.ReferenceOTU14809	0.8452	0.0319	k__Bacteria; p__Bacteroidetes; c__[Rhodothermi]; o__[Rhodothermales]; f__Rhodothermaceae; g__Rubricoccus; s__
New.CleanUp.ReferenceOTU15000	0.8452	0.0319	k__Bacteria; p__Proteobacteria; c__Deltaproteobacteria; o__Myxococcales; f__Nannocystaceae; g__Nannocystis; s__
New.CleanUp.ReferenceOTU15410	-0.8452	0.0319	k__Bacteria; p__Planctomycetes; c__Planctomycetia; o__Gemmatales; f__Gemmataceae; g__; s__
New.CleanUp.ReferenceOTU17111	0.8452	0.0319	Unassigned
New.CleanUp.ReferenceOTU17406	0.8452	0.0319	k__Bacteria; p__Proteobacteria; c__Deltaproteobacteria; o__Bdellovibrionales; f__Bacteriovoracaceae; g__; s__
New.CleanUp.ReferenceOTU17558	0.8452	0.0319	Unassigned
New.CleanUp.ReferenceOTU17708	0.8452	0.0319	k__Bacteria; p__Proteobacteria; c__Deltaproteobacteria; o__Spirobacillales; f__; g__; s__
New.CleanUp.ReferenceOTU18117	-0.8452	0.0319	k__Bacteria; p__Proteobacteria; c__Betaproteobacteria; o__Burkholderiales; f__Comamonadaceae; g__; s__
New.CleanUp.ReferenceOTU19353	0.8452	0.0319	k__Bacteria; p__Bacteroidetes; c__Sphingobacteriia; o__Sphingobacteriales; f__Sphingobacteriaceae; g__; s__
572889	-0.8281	0.0406	k__Bacteria; p__Fusobacteria; c__Fusobacteriia; o__Fusobacteriales; f__Fusobacteriaceae; g__Fusobacterium; s__
1120966	0.8281	0.0406	k__Bacteria; p__Proteobacteria; c__Betaproteobacteria; o__Burkholderiales; f__Comamonadaceae; g__Acidovorax
4408928	0.8281	0.0406	k__Bacteria; p__Proteobacteria; c__Alphaproteobacteria; o__Sphingomonadales; f__Sphingomonadaceae; g__Novosphingobium; s__
1004022	0.8281	0.0406	k__Bacteria; p__Proteobacteria; c__Gammaproteobacteria; o__Xanthomonadales; f__Xanthomonadaceae; g__Luteimonas; s__
610462	0.8281	0.0406	k__Bacteria; p__Proteobacteria; c__Gammaproteobacteria; o__Enterobacteriales; f__Enterobacteriaceae; g__Providencia; s__
782955	0.8281	0.0406	k__Bacteria; p__Bacteroidetes; c__Cytophagia; o__Cytophagales; f__Cytophagaceae; g__Hymenobacter; s__
805174	0.8281	0.0406	k__Bacteria; p__Bacteroidetes; c__Cytophagia; o__Cytophagales; f__Cytophagaceae; g__Hymenobacter; s__
New.CleanUp.ReferenceOTU1602	0.8281	0.0406	k__Bacteria; p__Proteobacteria; c__Alphaproteobacteria; o__Sphingomonadales; f__Sphingomonadaceae; g__Kaistobacter; s__
New.CleanUp.ReferenceOTU1965	0.8281	0.0406	k__Bacteria; p__Bacteroidetes; c__Sphingobacteriia; o__Sphingobacteriales; f__Sphingobacteriaceae; g__; s__
New.CleanUp.ReferenceOTU3689	0.8281	0.0406	k__Bacteria; p__Bacteroidetes; c__Cytophagia; o__Cytophagales; f__Cytophagaceae; g__Hymenobacter; s__
New.CleanUp.ReferenceOTU4227	0.8281	0.0406	k__Bacteria; p__Bacteroidetes; c__[Saprospirae]; o__[Saprospirales]; f__Saprospiraceae; g__; s__
New.CleanUp.ReferenceOTU5264	0.8281	0.0406	Unassigned
New.CleanUp.ReferenceOTU5644	0.8281	0.0406	k__Bacteria; p__Proteobacteria; c__Deltaproteobacteria; o__Spirobacillales; f__; g__; s__
New.CleanUp.ReferenceOTU5964	0.8281	0.0406	k__Bacteria; p__Bacteroidetes; c__[Saprospirae]; o__[Saprospirales]; f__Chitinophagaceae; g__Segetibacter; s__
New.CleanUp.ReferenceOTU6333	0.8281	0.0406	k__Bacteria; p__Proteobacteria; c__Alphaproteobacteria; o__Caulobacterales; f__Caulobacteraceae; g__Mycoplana; s__
New.CleanUp.ReferenceOTU6510	0.8281	0.0406	k__Bacteria; p__Cyanobacteria; c__Oscillatoriophycideae; o__Oscillatoriales; f__Phormidiaceae; g__Phormidium; s__
New.CleanUp.ReferenceOTU8028	0.8281	0.0406	k__Bacteria; p__Bacteroidetes; c__Cytophagia; o__Cytophagales; f__Cytophagaceae; g__Hymenobacter; s__
New.CleanUp.ReferenceOTU8948	0.8281	0.0406	k__Bacteria; p__Bacteroidetes; c__Cytophagia; o__Cytophagales; f__Cytophagaceae; g__Spirosoma; s__
New.CleanUp.ReferenceOTU9894	0.8281	0.0406	Unassigned
New.CleanUp.ReferenceOTU10050	0.8281	0.0406	k__Bacteria; p__Bacteroidetes; c__Cytophagia; o__Cytophagales; f__Cytophagaceae; g__Hymenobacter; s__
New.CleanUp.ReferenceOTU10749	0.8281	0.0406	k__Bacteria; p__Proteobacteria; c__Alphaproteobacteria; o__Rhodobacterales; f__Rhodobacteraceae; g__Rhodobacter; s__
New.CleanUp.ReferenceOTU10857	0.8281	0.0406	k__Bacteria; p__Acidobacteria; c__Acidobacteria-6; o__iii1-15; f__; g__; s__
New.CleanUp.ReferenceOTU12783	0.8281	0.0406	k__Bacteria; p__Proteobacteria; c__Alphaproteobacteria; o__Sphingomonadales; f__Sphingomonadaceae; g__Kaistobacter; s__
New.CleanUp.ReferenceOTU15436	0.8281	0.0406	k__Bacteria; p__Cyanobacteria; c__Nostocophycideae; o__Nostocales; f__Nostocaceae; g__; s__
New.CleanUp.ReferenceOTU16203	0.8281	0.0406	k__Bacteria; p__Proteobacteria; c__Alphaproteobacteria; o__Rhodospirillales; f__Acetobacteraceae; g__Roseococcus; s__
New.CleanUp.ReferenceOTU18131	-0.8281	0.0406	k__Bacteria; p__Actinobacteria; c__Actinobacteria; o__Actinomycetales; f__Nakamurellaceae; g__; s__
New.CleanUp.ReferenceOTU18808	0.8281	0.0406	k__Bacteria; p__Bacteroidetes; c__Cytophagia; o__Cytophagales; f__Cytophagaceae; g__Adhaeribacter; s__
New.CleanUp.ReferenceOTU19250	0.8281	0.0406	k__Bacteria; p__Bacteroidetes; c__[Rhodothermi]; o__[Rhodothermales]; f__Rhodothermaceae; g__Rubricoccus; s__
New.CleanUp.ReferenceOTU19310	0.8281	0.0406	k__Bacteria; p__Bacteroidetes; c__[Saprospirae]; o__[Saprospirales]; f__Chitinophagaceae; g__; s__
1119063	0.8197	0.0453	k__Bacteria; p__Bacteroidetes; c__Flavobacteriia; o__Flavobacteriales; f__Flavobacteriaceae; g__Flavobacterium; s__
4346564	0.8197	0.0453	k__Bacteria; p__Bacteroidetes; c__Flavobacteriia; o__Flavobacteriales; f__Flavobacteriaceae; g__Flavobacterium; s__
249034	0.8197	0.0453	k__Bacteria; p__Proteobacteria; c__Alphaproteobacteria; o__; f__; g__; s__
1065314	0.8197	0.0453	k__Bacteria; p__Bacteroidetes; c__Cytophagia; o__Cytophagales; f__Cytophagaceae; g__Sporocytophaga; s__
1132926	0.8197	0.0453	k__Bacteria; p__[Thermi]; c__Deinococci; o__Deinococcales; f__Deinococcaceae; g__Deinococcus; s__
2994818	0.8197	0.0453	k__Bacteria; p__Proteobacteria; c__Alphaproteobacteria; o__Sphingomonadales; f__; g__; s__
789806	0.8197	0.0453	k__Bacteria; p__Bacteroidetes; c__Cytophagia; o__Cytophagales; f__Cytophagaceae; g__Hymenobacter; s__
New.ReferenceOTU134	0.8197	0.0453	k__Bacteria; p__Bacteroidetes; c__Cytophagia; o__Cytophagales; f__Cytophagaceae; g__Spirosoma; s__
New.ReferenceOTU11	0.8197	0.0453	k__Bacteria; p__Bacteroidetes; c__Flavobacteriia; o__Flavobacteriales; f__Flavobacteriaceae; g__Flavobacterium; s__
New.CleanUp.ReferenceOTU523	0.8197	0.0453	k__Bacteria; p__Cyanobacteria; c__Synechococcophycideae; o__Pseudanabaenales; f__Pseudanabaenaceae; g__Pseudanabaena; s__
New.CleanUp.ReferenceOTU2506	0.8197	0.0453	k__Bacteria; p__Proteobacteria; c__Deltaproteobacteria; o__Bdellovibrionales; f__Bacteriovoracaceae; g__; s__
New.CleanUp.ReferenceOTU4431	0.8197	0.0453	Unassigned
New.CleanUp.ReferenceOTU9723	0.8197	0.0453	k__Bacteria; p__Bacteroidetes; c__Sphingobacteriia; o__Sphingobacteriales; f__; g__; s__
New.CleanUp.ReferenceOTU11751	0.8197	0.0453	Unassigned
New.CleanUp.ReferenceOTU14608	0.8197	0.0453	k__Bacteria; p__Cyanobacteria; c__Oscillatoriophycideae; o__Chroococcales; f__Xenococcaceae; g__; s__
New.CleanUp.ReferenceOTU14820	0.8197	0.0453	Unassigned
New.CleanUp.ReferenceOTU17507	0.8197	0.0453	k__Bacteria; p__Cyanobacteria; c__Synechococcophycideae; o__Pseudanabaenales; f__Pseudanabaenaceae; g__Leptolyngbya; s__
New.CleanUp.ReferenceOTU19169	0.8197	0.0453	k__Bacteria; p__Bacteroidetes; c__Cytophagia; o__Cytophagales; f__Cytophagaceae; g__Hymenobacter; s__
927623	0.8117	0.0499	k__Bacteria; p__Bacteroidetes; c__Cytophagia; o__Cytophagales; f__Cytophagaceae; g__Hymenobacter; s__
610107	0.8117	0.0499	k__Bacteria; p__Bacteroidetes; c__Cytophagia; o__Cytophagales; f__Cytophagaceae; g__Flectobacillus; s__
593908	0.8117	0.0499	k__Bacteria; p__Proteobacteria; c__Gammaproteobacteria; o__Xanthomonadales; f__Xanthomonadaceae; g__; s__
35188	0.8117	0.0499	k__Bacteria; p__Cyanobacteria; c__Oscillatoriophycideae; o__Oscillatoriales; f__Phormidiaceae; g__Phormidium; s__

Table S2 Supplementary Material. Microbialite Ca:Mg ratio Spearman tests for Alphaproteobacteria (OTU level) results, cutoff= Spearman rho > 0.9, *p* <0.02

OTU ID	Spearman rho	p value	taxonomy
593441	-0.9856	0.0000	k__Bacteria; p__Proteobacteria; c__Alphaproteobacteria; o__Rhodobacterales; f__Rhodobacteraceae; g__Anaerospora; s__
New.CleanUp.ReferenceOTU19464	-0.9856	0.0000	k__Bacteria; p__Proteobacteria; c__Alphaproteobacteria; o__Sphingomonadales; f__Sphingomonadaceae; g__Novosphingobium; s__
807415	-0.9710	0.0003	k__Bacteria; p__Proteobacteria; c__Alphaproteobacteria; o__Rhodobacterales; f__Rhodobacteraceae; g__Rhodobacter; s__
822853	-0.9429	0.0023	k__Bacteria; p__Proteobacteria; c__Alphaproteobacteria; o__Sphingomonadales; f__Sphingomonadaceae; g__; s__
4479555	-0.9429	0.0023	k__Bacteria; p__Proteobacteria; c__Alphaproteobacteria; o__Rhodospirillales; f__Acetobacteraceae
4614	0.9411	0.0025	k__Bacteria; p__Proteobacteria; c__Alphaproteobacteria; o__Rhizobiales; f__Hyphomicrobiaceae; g__Pedomicrobium; s__
645729	-0.9411	0.0025	k__Bacteria; p__Proteobacteria; c__Alphaproteobacteria; o__Sphingomonadales; f__Sphingomonadaceae; g__; s__
2228264	-0.9411	0.0025	k__Bacteria; p__Proteobacteria; c__Alphaproteobacteria; o__Rhodobacterales; f__Rhodobacteraceae; g__Rubellimicrobium; s__
New.CleanUp.ReferenceOTU3070	-0.9411	0.0025	k__Bacteria; p__Proteobacteria; c__Alphaproteobacteria; o__Sphingomonadales; f__Sphingomonadaceae; g__; s__
New.CleanUp.ReferenceOTU4511	-0.9411	0.0025	k__Bacteria; p__Proteobacteria; c__Alphaproteobacteria; o__Rhodobacterales; f__Rhodobacteraceae
New.CleanUp.ReferenceOTU13612	0.9411	0.0025	k__Bacteria; p__Proteobacteria; c__Alphaproteobacteria; o__Rhizobiales; f__Phyllobacteriaceae
New.CleanUp.ReferenceOTU14798	-0.9411	0.0025	k__Bacteria; p__Proteobacteria; c__Alphaproteobacteria; o__BD7-3; f__; g__; s__
365967	-0.9276	0.0045	k__Bacteria; p__Proteobacteria; c__Alphaproteobacteria; o__Rhodobacterales; f__Rhodobacteraceae; g__Rubellimicrobium; s__
818854	-0.9276	0.0045	k__Bacteria; p__Proteobacteria; c__Alphaproteobacteria; o__Rhodobacterales; f__Rhodobacteraceae; g__Rhodobacter; s__
4312153	-0.9276	0.0045	k__Bacteria; p__Proteobacteria; c__Alphaproteobacteria; o__Sphingomonadales; f__Sphingomonadaceae; g__; s__
489457	-0.9276	0.0045	k__Bacteria; p__Proteobacteria; c__Alphaproteobacteria; o__Sphingomonadales; f__Sphingomonadaceae; g__; s__
New.CleanUp.ReferenceOTU15572	-0.9276	0.0045	k__Bacteria; p__Proteobacteria; c__Alphaproteobacteria; o__Sphingomonadales; f__Sphingomonadaceae; g__; s__
1126769	-0.9258	0.0048	k__Bacteria; p__Proteobacteria; c__Alphaproteobacteria; o__Caulobacterales; f__Caulobacteraceae; g__Mycoplana; s__
111797	-0.9258	0.0048	k__Bacteria; p__Proteobacteria; c__Alphaproteobacteria; o__Rhizobiales
690867	-0.9258	0.0048	k__Bacteria; p__Proteobacteria; c__Alphaproteobacteria; o__Sphingomonadales; f__; g__; s__
New.CleanUp.ReferenceOTU5811	-0.9258	0.0048	k__Bacteria; p__Proteobacteria; c__Alphaproteobacteria; o__Sphingomonadales; f__Sphingomonadaceae; g__; s__
New.CleanUp.ReferenceOTU10170	-0.9258	0.0048	k__Bacteria; p__Proteobacteria; c__Alphaproteobacteria; o__Sphingomonadales; f__Sphingomonadaceae; g__; s__
New.CleanUp.ReferenceOTU10344	-0.9258	0.0048	k__Bacteria; p__Proteobacteria; c__Alphaproteobacteria; o__Rhodobacterales; f__Rhodobacteraceae; g__Rubellimicrobium; s__
New.CleanUp.ReferenceOTU16485	-0.9258	0.0048	k__Bacteria; p__Proteobacteria; c__Alphaproteobacteria; o__Rhodobacterales; f__Rhodobacteraceae; g__Rubellimicrobium; s__
1554680	-0.9122	0.0076	k__Bacteria; p__Proteobacteria; c__Alphaproteobacteria; o__Sphingomonadales; f__; g__; s__
798621	-0.9122	0.0076	k__Bacteria; p__Proteobacteria; c__Alphaproteobacteria; o__Sphingomonadales; f__; g__; s__

Table S3 Supplementary Material. Microbialite A) N:Mg ratio and C_{org}:Mg ratio Spearman tests for Planctomycetes (OTU level) results, cutoff= Spearman rho > 0.8, *p* <0.05

A) N:Mg

OTU ID	Spearman rho	p value	taxonomy
2617885	0.9429	0.0023	k__Bacteria; p__Planctomycetes; c__Planctomycetia; o__Gemmatales; f__Gemmataceae; g__Gemmata; s__
4397065	0.8452	0.0319	k__Bacteria; p__Planctomycetes; c__Planctomycetia; o__Planctomycetales; f__Planctomycetaceae; g__Planctomyces; s__
592393	0.8452	0.0319	k__Bacteria; p__Planctomycetes; c__Planctomycetia; o__Pirellulales; f__Pirellulaceae; g__; s__
327231	0.8452	0.0319	k__Bacteria; p__Planctomycetes; c__Planctomycetia; o__Pirellulales; f__Pirellulaceae; g__; s__
4374557	0.8452	0.0319	k__Bacteria; p__Planctomycetes; c__Planctomycetia; o__Pirellulales; f__Pirellulaceae; g__; s__
818655	0.8452	0.0319	k__Bacteria; p__Planctomycetes; c__Planctomycetia; o__Pirellulales; f__Pirellulaceae; g__; s__
569946	0.8452	0.0319	k__Bacteria; p__Planctomycetes; c__Planctomycetia; o__Pirellulales; f__Pirellulaceae; g__; s__
558165	0.8452	0.0319	k__Bacteria; p__Planctomycetes; c__Planctomycetia; o__Pirellulales; f__Pirellulaceae; g__; s__
823761	0.8452	0.0319	k__Bacteria; p__Planctomycetes; c__Planctomycetia; o__Pirellulales; f__Pirellulaceae; g__; s__
1106491	0.8452	0.0319	k__Bacteria; p__Planctomycetes; c__C6; o__d113; f__; g__; s__
New.ReferenceOTU27	0.8452	0.0319	k__Bacteria; p__Planctomycetes; c__Planctomycetia; o__Pirellulales; f__Pirellulaceae; g__; s__
New.CleanUp.ReferenceOTU5097	0.8452	0.0319	k__Bacteria; p__Planctomycetes; c__Planctomycetia; o__Pirellulales; f__Pirellulaceae; g__; s__
New.CleanUp.ReferenceOTU14522	-0.8452	0.0319	k__Bacteria; p__Planctomycetes; c__Phycisphaerae; o__WD2101; f__; g__; s__
New.CleanUp.ReferenceOTU15950	0.8452	0.0319	k__Bacteria; p__Planctomycetes; c__Planctomycetia; o__Pirellulales; f__Pirellulaceae; g__; s__
New.CleanUp.ReferenceOTU17198	0.8452	0.0319	k__Bacteria; p__Planctomycetes; c__Planctomycetia; o__Gemmatales; f__Gemmataceae; g__Gemmata; s__
New.CleanUp.ReferenceOTU17959	0.8452	0.0319	k__Bacteria; p__Planctomycetes; c__Planctomycetia; o__Pirellulales; f__Pirellulaceae; g__; s__
150298	0.8281	0.0406	k__Bacteria; p__Planctomycetes; c__Planctomycetia; o__Pirellulales; f__Pirellulaceae; g__; s__
545931	0.8281	0.0406	k__Bacteria; p__Planctomycetes; c__Planctomycetia; o__Pirellulales; f__Pirellulaceae; g__; s__
263866	0.8281	0.0406	k__Bacteria; p__Planctomycetes; c__Planctomycetia; o__Pirellulales; f__Pirellulaceae; g__; s__
321503	0.8281	0.0406	k__Bacteria; p__Planctomycetes; c__Planctomycetia; o__Pirellulales; f__Pirellulaceae; g__; s__
4375216	0.8281	0.0406	k__Bacteria; p__Planctomycetes; c__Planctomycetia; o__Pirellulales; f__Pirellulaceae; g__; s__
New.CleanUp.ReferenceOTU4313	0.8281	0.0406	k__Bacteria; p__Planctomycetes; c__Planctomycetia; o__Pirellulales; f__Pirellulaceae; g__; s__

B) C_{org}:Mg

OTU ID	Spearman rho	p value	taxonomy
2617885	0.9429	0.0023	k__Bacteria; p__Planctomycetes; c__Planctomycetia; o__Gemmatales; f__Gemmataceae; g__Gemmata; s__
4397065	0.8452	0.0319	k__Bacteria; p__Planctomycetes; c__Planctomycetia; o__Planctomycetales; f__Planctomycetaceae; g__Planctomyces; s__
592393	0.8452	0.0319	k__Bacteria; p__Planctomycetes; c__Planctomycetia; o__Pirellulales; f__Pirellulaceae; g__; s__
327231	0.8452	0.0319	k__Bacteria; p__Planctomycetes; c__Planctomycetia; o__Pirellulales; f__Pirellulaceae; g__; s__
4374557	0.8452	0.0319	k__Bacteria; p__Planctomycetes; c__Planctomycetia; o__Pirellulales; f__Pirellulaceae; g__; s__
818655	0.8452	0.0319	k__Bacteria; p__Planctomycetes; c__Planctomycetia; o__Pirellulales; f__Pirellulaceae; g__; s__
569946	0.8452	0.0319	k__Bacteria; p__Planctomycetes; c__Planctomycetia; o__Pirellulales; f__Pirellulaceae; g__; s__
558165	0.8452	0.0319	k__Bacteria; p__Planctomycetes; c__Planctomycetia; o__Pirellulales; f__Pirellulaceae; g__; s__
823761	0.8452	0.0319	k__Bacteria; p__Planctomycetes; c__Planctomycetia; o__Pirellulales; f__Pirellulaceae; g__; s__
1106491	0.8452	0.0319	k__Bacteria; p__Planctomycetes; c__C6; o__d113; f__; g__; s__
New.ReferenceOTU27	0.8452	0.0319	k__Bacteria; p__Planctomycetes; c__Planctomycetia; o__Pirellulales; f__Pirellulaceae; g__; s__
New.CleanUp.ReferenceOTU5097	0.8452	0.0319	k__Bacteria; p__Planctomycetes; c__Planctomycetia; o__Pirellulales; f__Pirellulaceae; g__; s__
New.CleanUp.ReferenceOTU14522	-0.8452	0.0319	k__Bacteria; p__Planctomycetes; c__Phycisphaerae; o__WD2101; f__; g__; s__
New.CleanUp.ReferenceOTU15950	0.8452	0.0319	k__Bacteria; p__Planctomycetes; c__Planctomycetia; o__Pirellulales; f__Pirellulaceae; g__; s__
New.CleanUp.ReferenceOTU17198	0.8452	0.0319	k__Bacteria; p__Planctomycetes; c__Planctomycetia; o__Gemmatales; f__Gemmataceae; g__Gemmata; s__
New.CleanUp.ReferenceOTU17959	0.8452	0.0319	k__Bacteria; p__Planctomycetes; c__Planctomycetia; o__Pirellulales; f__Pirellulaceae; g__; s__
150298	0.8281	0.0406	k__Bacteria; p__Planctomycetes; c__Planctomycetia; o__Pirellulales; f__Pirellulaceae; g__; s__
545931	0.8281	0.0406	k__Bacteria; p__Planctomycetes; c__Planctomycetia; o__Pirellulales; f__Pirellulaceae; g__; s__
263866	0.8281	0.0406	k__Bacteria; p__Planctomycetes; c__Planctomycetia; o__Pirellulales; f__Pirellulaceae; g__; s__
321503	0.8281	0.0406	k__Bacteria; p__Planctomycetes; c__Planctomycetia; o__Pirellulales; f__Pirellulaceae; g__; s__
4375216	0.8281	0.0406	k__Bacteria; p__Planctomycetes; c__Planctomycetia; o__Pirellulales; f__Pirellulaceae; g__; s__
New.CleanUp.ReferenceOTU4313	0.8281	0.0406	k__Bacteria; p__Planctomycetes; c__Planctomycetia; o__Pirellulales; f__Pirellulaceae; g__; s__

Table S4 Supplementary Material. Microbialite N:Ca ratio Spearman tests (OTU level) results for A) Bacteroidetes and B) Alpha and C) Betaproteobacteria, cutoff= Spearman rho > 0.8, p <0.04

A)

OTU ID	Soearman rho	p value	taxonomy
141302	0.9411	0.0025	k__Bacteria; p__Bacteroidetes; c__Flavobacteriia; o__Flavobacteriales; f__Flavobacteriaceae; g__Flavobacterium; s__
489865	0.9411	0.0025	k__Bacteria; p__Bacteroidetes; c__Flavobacteriia; o__Flavobacteriales; f__Flavobacteriaceae; g__Flavobacterium; s__
New.CleanUp.ReferenceOTU10285	0.9411	0.0025	k__Bacteria; p__Bacteroidetes; c__[Saprospirae]; o__[Saprospirales]; f__Chitinophagaceae; g__; s__
331473	0.8452	0.0319	k__Bacteria; p__Bacteroidetes; c__Cytophagia; o__Cytophagales; f__Cytophagaceae; g__Hymenobacter; s__
1116759	0.8452	0.0319	k__Bacteria; p__Bacteroidetes; c__Flavobacteriia; o__Flavobacteriales; f__Flavobacteriaceae; g__Flavobacterium; s__
363617	-0.8452	0.0319	k__Bacteria; p__Bacteroidetes; c__[Saprospirae]; o__[Saprospirales]; f__Chitinophagaceae; g__; s__
735569	0.8452	0.0319	k__Bacteria; p__Bacteroidetes; c__[Saprospirae]; o__[Saprospirales]; f__Chitinophagaceae; g__; s__
541746	0.8452	0.0319	k__Bacteria; p__Bacteroidetes; c__Cytophagia; o__Cytophagales; f__Cytophagaceae; g__Hymenobacter; s__
4410283	0.8452	0.0319	k__Bacteria; p__Bacteroidetes; c__Cytophagia; o__Cytophagales; f__Cytophagaceae; g__Hymenobacter; s__
1116398	0.8452	0.0319	k__Bacteria; p__Bacteroidetes; c__Flavobacteriia; o__Flavobacteriales; f__Flavobacteriaceae; g__Flavobacterium; s__
73825	0.8452	0.0319	k__Bacteria; p__Bacteroidetes; c__Flavobacteriia; o__Flavobacteriales; f__Flavobacteriaceae; g__Flavobacterium; s__
719030	0.8452	0.0319	k__Bacteria; p__Bacteroidetes; c__Cytophagia; o__Cytophagales; f__Cytophagaceae; g__Hymenobacter; s__
1081614	0.8452	0.0319	k__Bacteria; p__Bacteroidetes; c__Flavobacteriia; o__Flavobacteriales; f__Flavobacteriaceae; g__Flavobacterium; s__
570086	0.8452	0.0319	k__Bacteria; p__Bacteroidetes; c__Flavobacteriia; o__Flavobacteriales; f__Cryomorphaceae; g__Fluviicola; s__
1131830	-0.8452	0.0319	k__Bacteria; p__Bacteroidetes; c__Cytophagia; o__Cytophagales; f__Cytophagaceae
994982	0.8452	0.0319	k__Bacteria; p__Bacteroidetes; c__Flavobacteriia; o__Flavobacteriales; f__Flavobacteriaceae; g__Flavobacterium; s__
1146920	0.8452	0.0319	k__Bacteria; p__Bacteroidetes; c__Cytophagia; o__Cytophagales; f__Cytophagaceae; g__Hymenobacter; s__
988172	0.8452	0.0319	k__Bacteria; p__Bacteroidetes; c__Cytophagia; o__Cytophagales; f__Cytophagaceae; g__Hymenobacter; s__
827674	0.8452	0.0319	k__Bacteria; p__Bacteroidetes; c__Cytophagia; o__Cytophagales; f__Cytophagaceae; g__Hymenobacter; s__
574686	0.8452	0.0319	k__Bacteria; p__Bacteroidetes; c__Flavobacteriia; o__Flavobacteriales; f__Flavobacteriaceae; g__Flavobacterium; s__
745482	-0.8452	0.0319	k__Bacteria; p__Bacteroidetes; c__[Saprospirae]; o__[Saprospirales]; f__Chitinophagaceae; g__; s__
New.ReferenceOTU75	0.8452	0.0319	k__Bacteria; p__Bacteroidetes; c__Cytophagia; o__Cytophagales; f__Cytophagaceae; g__Adhaeribacter; s__
New.ReferenceOTU25	-0.8452	0.0319	k__Bacteria; p__Bacteroidetes; c__[Saprospirae]; o__[Saprospirales]; f__Chitinophagaceae; g__Flavisolibacter; s__
New.CleanUp.ReferenceOTU17	0.8452	0.0319	k__Bacteria; p__Bacteroidetes; c__Cytophagia; o__Cytophagales; f__Cytophagaceae; g__Adhaeribacter; s__
New.CleanUp.ReferenceOTU202	0.8452	0.0319	k__Bacteria; p__Bacteroidetes; c__Sphingobacteriia; o__Sphingobacteriales; f__; g__; s__
New.CleanUp.ReferenceOTU962	-0.8452	0.0319	k__Bacteria; p__Bacteroidetes; c__Cytophagia; o__Cytophagales; f__Cytophagaceae; g__; s__
New.CleanUp.ReferenceOTU1618	-0.8452	0.0319	k__Bacteria; p__Bacteroidetes; c__Cytophagia; o__Cytophagales; f__Cytophagaceae; g__; s__
New.CleanUp.ReferenceOTU1808	0.8452	0.0319	k__Bacteria; p__Bacteroidetes; c__Cytophagia; o__Cytophagales; f__Cytophagaceae; g__Hymenobacter; s__
New.CleanUp.ReferenceOTU2042	0.8452	0.0319	k__Bacteria; p__Bacteroidetes; c__Cytophagia; o__Cytophagales; f__Cytophagaceae; g__Hymenobacter; s__
New.CleanUp.ReferenceOTU2757	-0.8452	0.0319	k__Bacteria; p__Bacteroidetes; c__Sphingobacteriia; o__Sphingobacteriales; f__; g__; s__
New.CleanUp.ReferenceOTU3819	0.8452	0.0319	k__Bacteria; p__Bacteroidetes; c__Flavobacteriia; o__Flavobacteriales; f__Flavobacteriaceae; g__Flavobacterium; s__
New.CleanUp.ReferenceOTU6097	-0.8452	0.0319	k__Bacteria; p__Bacteroidetes; c__[Saprospirae]; o__[Saprospirales]; f__Chitinophagaceae; g__; s__
New.CleanUp.ReferenceOTU6892	-0.8452	0.0319	k__Bacteria; p__Bacteroidetes; c__[Saprospirae]; o__[Saprospirales]; f__Chitinophagaceae; g__; s__
New.CleanUp.ReferenceOTU7354	-0.8452	0.0319	k__Bacteria; p__Bacteroidetes; c__Flavobacteriia; o__Flavobacteriales; f__Cryomorphaceae; g__Fluviicola; s__
New.CleanUp.ReferenceOTU7525	-0.8452	0.0319	k__Bacteria; p__Bacteroidetes; c__Cytophagia; o__Cytophagales; f__Cytophagaceae; g__; s__
New.CleanUp.ReferenceOTU7576	0.8452	0.0319	k__Bacteria; p__Bacteroidetes; c__Cytophagia; o__Cytophagales; f__Cytophagaceae; g__Hymenobacter; s__
New.CleanUp.ReferenceOTU9008	-0.8452	0.0319	k__Bacteria; p__Bacteroidetes; c__Sphingobacteriia; o__Sphingobacteriales; f__; g__; s__
New.CleanUp.ReferenceOTU9283	-0.8452	0.0319	k__Bacteria; p__Bacteroidetes; c__Cytophagia; o__Cytophagales; f__; g__; s__
New.CleanUp.ReferenceOTU9603	-0.8452	0.0319	k__Bacteria; p__Bacteroidetes; c__[Saprospirae]; o__[Saprospirales]; f__Chitinophagaceae; g__; s__
New.CleanUp.ReferenceOTU10418	0.8452	0.0319	k__Bacteria; p__Bacteroidetes; c__Cytophagia; o__Cytophagales; f__Cytophagaceae; g__Adhaeribacter; s__
New.CleanUp.ReferenceOTU12137	0.8452	0.0319	k__Bacteria; p__Bacteroidetes; c__Cytophagia; o__Cytophagales; f__Cytophagaceae; g__Hymenobacter; s__
New.CleanUp.ReferenceOTU12435	0.8452	0.0319	k__Bacteria; p__Bacteroidetes; c__Cytophagia; o__Cytophagales; f__Cytophagaceae; g__Hymenobacter; s__
New.CleanUp.ReferenceOTU12863	-0.8452	0.0319	k__Bacteria; p__Bacteroidetes; c__[Saprospirae]; o__[Saprospirales]; f__Chitinophagaceae; g__; s__
New.CleanUp.ReferenceOTU13117	0.8452	0.0319	k__Bacteria; p__Bacteroidetes; c__[Saprospirae]; o__[Saprospirales]; f__Chitinophagaceae; g__; s__
New.CleanUp.ReferenceOTU13692	0.8452	0.0319	k__Bacteria; p__Bacteroidetes; c__Flavobacteriia; o__Flavobacteriales; f__Cryomorphaceae; g__Fluviicola; s__
New.CleanUp.ReferenceOTU14809	0.8452	0.0319	k__Bacteria; p__Bacteroidetes; c__[Rhodothermi]; o__[Rhodothermales]; f__Rhodothermaceae; g__Rubricoccus; s__
New.CleanUp.ReferenceOTU15472	-0.8452	0.0319	k__Bacteria; p__Bacteroidetes; c__[Saprospirae]; o__[Saprospirales]; f__Chitinophagaceae; g__Flavisolibacter; s__
New.CleanUp.ReferenceOTU16501	-0.8452	0.0319	k__Bacteria; p__Bacteroidetes; c__[Saprospirae]; o__[Saprospirales]; f__Saprospiraceae; g__; s__
New.CleanUp.ReferenceOTU17701	-0.8452	0.0319	k__Bacteria; p__Bacteroidetes; c__Flavobacteriia; o__Flavobacteriales; f__Cryomorphaceae; g__; s__
New.CleanUp.ReferenceOTU19353	0.8452	0.0319	k__Bacteria; p__Bacteroidetes; c__Sphingobacteriia; o__Sphingobacteriales; f__Sphingobacteriaceae; g__; s__

B)

OTU ID	Spearman rho	p value	taxonomy
835594	-0.8827	0.0162	k__Bacteria; p__Proteobacteria; c__Alphaproteobacteria; o__Rhizobiales; f__Methylobacteriaceae; g__Methylobacterium; s__
854272	0.8533	0.0280	k__Bacteria; p__Proteobacteria; c__Alphaproteobacteria; o__Rhodobacterales; f__Rhodobacteraceae; g__; s__
539878	-0.8452	0.0319	k__Bacteria; p__Proteobacteria; c__Alphaproteobacteria; o__Rhizobiales; f__Phyllobacteriaceae; g__; s__
567840	-0.8452	0.0319	k__Bacteria; p__Proteobacteria; c__Alphaproteobacteria; o__Rhizobiales; f__Bradyrhizobiaceae; g__Bosea; s__genosp.
560770	-0.8452	0.0319	k__Bacteria; p__Proteobacteria; c__Alphaproteobacteria; o__Caulobacterales; f__Caulobacteraceae; g__; s__
580312	-0.8452	0.0319	k__Bacteria; p__Proteobacteria; c__Alphaproteobacteria; o__Sphingomonadales; f__Sphingomonadaceae; g__Sphingomonas; s__
143392	-0.8452	0.0319	k__Bacteria; p__Proteobacteria; c__Alphaproteobacteria; o__Sphingomonadales; f__Sphingomonadaceae; g__Kaistobacter; s__
849761	-0.8452	0.0319	k__Bacteria; p__Proteobacteria; c__Alphaproteobacteria; o__Sphingomonadales; f__Sphingomonadaceae; g__Sphingobium; s__
209507	-0.8452	0.0319	k__Bacteria; p__Proteobacteria; c__Alphaproteobacteria; o__Rhizobiales; f__Hyphomicrobiaceae; g__Devosia; s__
676066	-0.8452	0.0319	k__Bacteria; p__Proteobacteria; c__Alphaproteobacteria; o__Rhizobiales; f__Bradyrhizobiaceae
70100	-0.8452	0.0319	k__Bacteria; p__Proteobacteria; c__Alphaproteobacteria; o__Rhizobiales; f__Hyphomicrobiaceae; g__Rhodoplanes; s__
173252	-0.8452	0.0319	k__Bacteria; p__Proteobacteria; c__Alphaproteobacteria; o__Rhizobiales; f__; g__; s__
186867	-0.8452	0.0319	k__Bacteria; p__Proteobacteria; c__Alphaproteobacteria; o__Rickettsiales; f__Rickettsiaceae; g__Rickettsia
576136	-0.8452	0.0319	k__Bacteria; p__Proteobacteria; c__Alphaproteobacteria; o__Ellin329; f__; g__; s__
508780	-0.8452	0.0319	k__Bacteria; p__Proteobacteria; c__Alphaproteobacteria; o__Rhizobiales; f__Hyphomicrobiaceae; g__Rhodoplanes; s__
792078	-0.8452	0.0319	k__Bacteria; p__Proteobacteria; c__Alphaproteobacteria; o__Rhizobiales; f__; g__; s__
4319059	0.8452	0.0319	k__Bacteria; p__Proteobacteria; c__Alphaproteobacteria; o__Sphingomonadales; f__; g__; s__
New.ReferenceOTU33	-0.8452	0.0319	k__Bacteria; p__Proteobacteria; c__Alphaproteobacteria; o__Rhodospirillales; f__Acetobacteraceae; g__; s__
New.CleanUp.ReferenceOTU70	-0.8452	0.0319	k__Bacteria; p__Proteobacteria; c__Alphaproteobacteria; o__Rickettsiales; f__Rickettsiaceae; g__Rickettsia
New.CleanUp.ReferenceOTU289	-0.8452	0.0319	k__Bacteria; p__Proteobacteria; c__Alphaproteobacteria; o__Sphingomonadales; f__Sphingomonadaceae; g__Sphingobium; s__
New.CleanUp.ReferenceOTU2137	-0.8452	0.0319	k__Bacteria; p__Proteobacteria; c__Alphaproteobacteria; o__Rhizobiales; f__Hyphomicrobiaceae; g__Devosia; s__
New.CleanUp.ReferenceOTU2521	-0.8452	0.0319	k__Bacteria; p__Proteobacteria; c__Alphaproteobacteria; o__Caulobacterales; f__Caulobacteraceae; g__; s__
New.CleanUp.ReferenceOTU5262	-0.8452	0.0319	k__Bacteria; p__Proteobacteria; c__Alphaproteobacteria; o__Rhodospirillales; f__Acetobacteraceae
New.CleanUp.ReferenceOTU6274	-0.8452	0.0319	k__Bacteria; p__Proteobacteria; c__Alphaproteobacteria; o__Rickettsiales; f__Rickettsiaceae; g__Rickettsia; s__
New.CleanUp.ReferenceOTU7270	0.8452	0.0319	k__Bacteria; p__Proteobacteria; c__Alphaproteobacteria; o__Rhodobacterales; f__Rhodobacteraceae; g__Rhodobacter; s__
New.CleanUp.ReferenceOTU8248	-0.8452	0.0319	k__Bacteria; p__Proteobacteria; c__Alphaproteobacteria; o__Rickettsiales; f__; g__; s__
New.CleanUp.ReferenceOTU9148	-0.8452	0.0319	k__Bacteria; p__Proteobacteria; c__Alphaproteobacteria; o__Rickettsiales; f__Rickettsiaceae; g__Rickettsia
New.CleanUp.ReferenceOTU10074	-0.8452	0.0319	k__Bacteria; p__Proteobacteria; c__Alphaproteobacteria; o__Rhizobiales; f__Hyphomicrobiaceae; g__Devosia; s__
New.CleanUp.ReferenceOTU10529	-0.8452	0.0319	k__Bacteria; p__Proteobacteria; c__Alphaproteobacteria; o__Sphingomonadales; f__Sphingomonadaceae; g__Sphingomonas; s__
New.CleanUp.ReferenceOTU12242	-0.8452	0.0319	k__Bacteria; p__Proteobacteria; c__Alphaproteobacteria; o__Rhodospirillales; f__Rhodospirillaceae; g__; s__
New.CleanUp.ReferenceOTU12570	-0.8452	0.0319	k__Bacteria; p__Proteobacteria; c__Alphaproteobacteria; o__Caulobacterales; f__Caulobacteraceae; g__Mycoplana; s__
New.CleanUp.ReferenceOTU13300	-0.8452	0.0319	k__Bacteria; p__Proteobacteria; c__Alphaproteobacteria; o__Sphingomonadales; f__Sphingomonadaceae; g__Sphingomonas; s__
New.CleanUp.ReferenceOTU13375	-0.8452	0.0319	k__Bacteria; p__Proteobacteria; c__Alphaproteobacteria; o__Sphingomonadales; f__Sphingomonadaceae; g__Sphingomonas; s__
New.CleanUp.ReferenceOTU15046	-0.8452	0.0319	k__Bacteria; p__Proteobacteria; c__Alphaproteobacteria; o__Caulobacterales; f__Caulobacteraceae; g__Mycoplana; s__
New.CleanUp.ReferenceOTU15642	-0.8452	0.0319	k__Bacteria; p__Proteobacteria; c__Alphaproteobacteria; o__Rhodospirillales; f__Acetobacteraceae; g__; s__
New.CleanUp.ReferenceOTU16194	-0.8452	0.0319	k__Bacteria; p__Proteobacteria; c__Alphaproteobacteria; o__Sphingomonadales; f__Sphingomonadaceae; g__Sphingomonas; s__wittichii
New.CleanUp.ReferenceOTU17433	-0.8452	0.0319	k__Bacteria; p__Proteobacteria; c__Alphaproteobacteria; o__Rhodospirillales; f__Acetobacteraceae; g__; s__
New.CleanUp.ReferenceOTU18693	-0.8452	0.0319	k__Bacteria; p__Proteobacteria; c__Alphaproteobacteria; o__Sphingomonadales; f__Sphingomonadaceae; g__Sphingomonas; s__

C)

OTU ID	Spearman rho	p value	taxonomy
798634	-0.8452	0.0319	k__Bacteria; p__Proteobacteria; c__Betaproteobacteria; o__Burkholderiales; f__Comamonadaceae
436550	-0.8452	0.0319	k__Bacteria; p__Proteobacteria; c__Betaproteobacteria; o__Burkholderiales; f__Comamonadaceae; g__; s__
4441674	-0.8452	0.0319	k__Bacteria; p__Proteobacteria; c__Betaproteobacteria; o__Burkholderiales; f__Comamonadaceae; g__; s__
4482839	-0.8452	0.0319	k__Bacteria; p__Proteobacteria; c__Betaproteobacteria; o__Burkholderiales; f__Comamonadaceae; g__; s__
4438613	0.8452	0.0319	k__Bacteria; p__Proteobacteria; c__Betaproteobacteria; o__Burkholderiales; f__Comamonadaceae; g__; s__
731707	-0.8452	0.0319	k__Bacteria; p__Proteobacteria; c__Betaproteobacteria; o__Burkholderiales; f__Comamonadaceae; g__Hylemonella; s__
4410974	0.8452	0.0319	k__Bacteria; p__Proteobacteria; c__Betaproteobacteria; o__Burkholderiales; f__Comamonadaceae; g__Polaromonas; s__
203357	-0.8452	0.0319	k__Bacteria; p__Proteobacteria; c__Betaproteobacteria; o__Burkholderiales; f__Comamonadaceae; g__; s__
New.ReferenceOTU21	-0.8452	0.0319	k__Bacteria; p__Proteobacteria; c__Betaproteobacteria; o__Burkholderiales; f__Comamonadaceae
New.CleanUp.ReferenceOTU786	-0.8452	0.0319	k__Bacteria; p__Proteobacteria; c__Betaproteobacteria; o__A21b; f__UD5; g__; s__
New.CleanUp.ReferenceOTU2394	-0.8452	0.0319	k__Bacteria; p__Proteobacteria; c__Betaproteobacteria; o__Burkholderiales; f__Comamonadaceae; g__; s__
New.CleanUp.ReferenceOTU4149	-0.8452	0.0319	k__Bacteria; p__Proteobacteria; c__Betaproteobacteria; o__SC-I-84; f__; g__; s__
New.CleanUp.ReferenceOTU5697	-0.8452	0.0319	k__Bacteria; p__Proteobacteria; c__Betaproteobacteria; o__SC-I-84; f__; g__; s__
New.CleanUp.ReferenceOTU7040	-0.8452	0.0319	k__Bacteria; p__Proteobacteria; c__Betaproteobacteria; o__Burkholderiales; f__Comamonadaceae; g__; s__
New.CleanUp.ReferenceOTU7632	-0.8452	0.0319	k__Bacteria; p__Proteobacteria; c__Betaproteobacteria; o__Burkholderiales; f__Comamonadaceae
New.CleanUp.ReferenceOTU9780	-0.8452	0.0319	k__Bacteria; p__Proteobacteria; c__Betaproteobacteria; o__Rhodocyclales; f__Rhodocyclaceae
New.CleanUp.ReferenceOTU10194	-0.8452	0.0319	k__Bacteria; p__Proteobacteria; c__Betaproteobacteria; o__SC-I-84; f__; g__; s__
New.CleanUp.ReferenceOTU14103	-0.8452	0.0319	k__Bacteria; p__Proteobacteria; c__Betaproteobacteria; o__Burkholderiales; f__Comamonadaceae; g__Methylibium
New.CleanUp.ReferenceOTU16685	-0.8452	0.0319	k__Bacteria; p__Proteobacteria; c__Betaproteobacteria; o__Burkholderiales; f__Comamonadaceae; g__; s__
New.CleanUp.ReferenceOTU16915	-0.8452	0.0319	k__Bacteria; p__Proteobacteria; c__Betaproteobacteria; o__SC-I-84; f__; g__; s__
New.CleanUp.ReferenceOTU17185	-0.8452	0.0319	k__Bacteria; p__Proteobacteria; c__Betaproteobacteria; o__Burkholderiales; f__Alcaligenaceae; g__; s__
New.CleanUp.ReferenceOTU18801	-0.8452	0.0319	k__Bacteria; p__Proteobacteria; c__Betaproteobacteria; o__SC-I-84; f__; g__; s__

Table S5 Supplementary Material. Microbialite Nitrogen content Spearman test (OTU level) results, cutoff= Spearman rho > 0.8, p <0.05

OTU ID	Spearman rho	p value	Taxonomy
3114957	0.9122	0.0076	k__Bacteria; p__Chloroflexi; c__Chloroflexi; o__[Roseiflexales]; f__; g__; s__
1823053	0.8827	0.0162	k__Bacteria; p__Firmicutes; c__Bacilli; o__Lactobacillales; f__Streptococcaceae; g__Lactococcus; s__
854272	0.8827	0.0162	k__Bacteria; p__Proteobacteria; c__Alphaproteobacteria; o__Rhodobacterales; f__Rhodobacteraceae; g__; s__
141302	0.8804	0.0170	k__Bacteria; p__Bacteroidetes; c__Flavobacteriia; o__Flavobacteriales; f__Flavobacteriaceae; g__Flavobacterium; s__
489865	0.8804	0.0170	k__Bacteria; p__Bacteroidetes; c__Flavobacteriia; o__Flavobacteriales; f__Flavobacteriaceae; g__Flavobacterium; s__
823745	0.8804	0.0170	k__Bacteria; p__Proteobacteria; c__Gammaproteobacteria; o__Pasteurellales; f__Pasteurellaceae
New.CleanUp.ReferenceOTU10285	0.8804	0.0170	k__Bacteria; p__Bacteroidetes; c__[Saprospirae]; o__[Saprospirales]; f__Chitinophagaceae; g__; s__
226126	0.8452	0.0319	k__Bacteria; p__Cyanobacteria; c__Synechococcophycideae; o__Pseudanabaenales; f__Pseudanabaenaceae; g__Pseudanabaena; s__
538315	0.8452	0.0319	k__Bacteria; p__Actinobacteria; c__Actinobacteria; o__Actinomycetales; f__Pseudonocardiaceae; g__Pseudonocardia; s__
130710	0.8452	0.0319	k__Bacteria; p__Actinobacteria; c__Actinobacteria; o__Actinomycetales; f__Micrococcaceae; g__; s__
254635	0.8452	0.0319	k__Bacteria; p__Actinobacteria; c__Actinobacteria; o__Actinomycetales; f__Frankiaceae; g__; s__
113915	0.8452	0.0319	k__Bacteria; p__Proteobacteria; c__Gammaproteobacteria; o__Xanthomonadales; f__Xanthomonadaceae; g__Arenimonas; s__
272797	0.8452	0.0319	k__Bacteria; p__Proteobacteria; c__Gammaproteobacteria; o__Xanthomonadales; f__Xanthomonadaceae; g__Arenimonas; s__
New.ReferenceOTU97	0.8452	0.0319	k__Bacteria; p__Cyanobacteria; c__Chloroplast; o__Chlorophyta; f__; g__; s__
388951	0.8332	0.0379	k__Bacteria; p__Proteobacteria; c__Gammaproteobacteria; o__Pseudomonadales; f__Moraxellaceae; g__Acinetobacter; s__
1088120	0.8286	0.0404	k__Bacteria; p__Bacteroidetes; c__Sphingobacteriia; o__Sphingobacteriales; f__Sphingobacteriaceae; g__; s__
241071	0.8286	0.0404	k__Bacteria; p__Cyanobacteria; c__Synechococcophycideae; o__Pseudanabaenales; f__Pseudanabaenaceae; g__Pseudanabaena; s__
1084865	0.8286	0.0404	k__Bacteria; p__Firmicutes; c__Bacilli; o__Bacillales; f__Staphylococcaceae; g__Staphylococcus; s__
1050608	0.8281	0.0406	k__Bacteria; p__Actinobacteria; c__Actinobacteria; o__Actinomycetales; f__Corynebacteriaceae; g__Corynebacterium; s__
22668	0.8281	0.0406	k__Bacteria; p__Firmicutes; c__Clostridia; o__Clostridiales; f__Clostridiaceae; g__Candidatus Arthromitus; s__
513586	0.8281	0.0406	k__Bacteria; p__Planctomycetes; c__Phycisphaerae; o__WD2101; f__; g__; s__
572889	-0.8281	0.0406	k__Bacteria; p__Fusobacteria; c__Fusobacteriia; o__Fusobacteriales; f__Fusobacteriaceae; g__Fusobacterium; s__
833317	0.8281	0.0406	k__Bacteria; p__Firmicutes; c__Bacilli; o__Bacillales; f__Planococcaceae; g__Sporosarcina; s__
767235	0.8281	0.0406	k__Bacteria; p__Chloroflexi; c__Anaerolineae; o__Anaerolineales; f__Anaerolinaceae; g__T78; s__
4365229	0.8281	0.0406	k__Bacteria; p__Proteobacteria; c__Betaproteobacteria; o__Burkholderiales; f__Oxalobacteraceae; g__Janthinobacterium; s__lividum
244972	0.8281	0.0406	k__Bacteria; p__Proteobacteria; c__Alphaproteobacteria; o__Rhodospirillales; f__; g__; s__
4484466	0.8281	0.0406	k__Bacteria; p__Proteobacteria; c__Gammaproteobacteria; o__Enterobacteriales; f__Enterobacteriaceae; g__; s__
978664	0.8281	0.0406	k__Bacteria; p__Actinobacteria; c__Actinobacteria; o__Actinomycetales; f__Mycobacteriaceae; g__Mycobacterium; s__
1109623	0.8281	0.0406	k__Bacteria; p__Proteobacteria; c__Gammaproteobacteria; o__Enterobacteriales; f__Enterobacteriaceae; g__; s__
New.CleanUp.ReferenceOTU6551	0.8281	0.0406	k__Bacteria; p__Proteobacteria; c__Gammaproteobacteria; o__Enterobacteriales; f__Enterobacteriaceae
New.CleanUp.ReferenceOTU8032	0.8281	0.0406	k__Bacteria; p__Cyanobacteria; c__Nostocophycideae; o__Stigonematales; f__Rivulariaceae; g__Rivularia; s__
New.CleanUp.ReferenceOTU18131	-0.8281	0.0406	k__Bacteria; p__Actinobacteria; c__Actinobacteria; o__Actinomycetales; f__Nakamurellaceae; g__; s__
New.CleanUp.ReferenceOTU19098	0.8281	0.0406	k__Bacteria; p__Bacteroidetes; c__[Saprospirae]; o__[Saprospirales]; f__Chitinophagaceae; g__Sediminibacterium; s__
564704	0.8197	0.0453	k__Bacteria; p__Firmicutes; c__Bacilli; o__Lactobacillales; f__Leuconostocaceae; g__; s__
873518	0.8117	0.0499	k__Bacteria; p__Proteobacteria; c__Deltaproteobacteria; o__Myxococcales; f__; g__; s__
1117222	0.8117	0.0499	k__Bacteria; p__Bacteroidetes; c__Flavobacteriia; o__Flavobacteriales; f__Flavobacteriaceae; g__Flavobacterium; s__
495084	0.8117	0.0499	k__Bacteria; p__Firmicutes; c__Clostridia; o__Clostridiales; f__[Tissierellaceae]; g__Anaerococcus; s__

Table S6 Supplementary Material. Microbialite Copper content Spearman test for Alphaproteobacteria (OTU level) results, cutoff= Spearman rho > 0.8, p <0.05

OTU ID	Spearman rho	p value	taxonomy
645729	0.9549	0.0011	k__Bacteria; p__Proteobacteria; c__Alphaproteobacteria; o__Sphingomonadales; f__Sphingomonadaceae; g__; s__
2228264	0.9549	0.0011	k__Bacteria; p__Proteobacteria; c__Alphaproteobacteria; o__Rhodobacterales; f__Rhodobacteraceae; g__Rubellimicrobium; s__
New.CleanUp.ReferenceOTU3070	0.9549	0.0011	k__Bacteria; p__Proteobacteria; c__Alphaproteobacteria; o__Sphingomonadales; f__Sphingomonadaceae; g__; s__
New.CleanUp.ReferenceOTU4511	0.9549	0.0011	k__Bacteria; p__Proteobacteria; c__Alphaproteobacteria; o__Rhodobacterales; f__Rhodobacteraceae
New.CleanUp.ReferenceOTU14798	0.9549	0.0011	k__Bacteria; p__Proteobacteria; c__Alphaproteobacteria; o__BD7-3; f__; g__; s__
New.CleanUp.ReferenceOTU19016	0.9412	0.0025	k__Bacteria; p__Proteobacteria; c__Alphaproteobacteria; o__Rhodobacterales; f__Rhodobacteraceae; g__Rubellimicrobium; s__
111797	0.9393	0.0027	k__Bacteria; p__Proteobacteria; c__Alphaproteobacteria; o__Rhizobiales
New.CleanUp.ReferenceOTU5811	0.9393	0.0027	k__Bacteria; p__Proteobacteria; c__Alphaproteobacteria; o__Sphingomonadales; f__Sphingomonadaceae; g__; s__
New.CleanUp.ReferenceOTU10170	0.9393	0.0027	k__Bacteria; p__Proteobacteria; c__Alphaproteobacteria; o__Sphingomonadales; f__Sphingomonadaceae; g__; s__
New.CleanUp.ReferenceOTU10344	0.9393	0.0027	k__Bacteria; p__Proteobacteria; c__Alphaproteobacteria; o__Rhodobacterales; f__Rhodobacteraceae; g__Rubellimicrobium; s__
New.CleanUp.ReferenceOTU16485	0.9393	0.0027	k__Bacteria; p__Proteobacteria; c__Alphaproteobacteria; o__Rhodobacterales; f__Rhodobacteraceae; g__Rubellimicrobium; s__
1052559	0.9276	0.0045	k__Bacteria; p__Proteobacteria; c__Alphaproteobacteria; o__Sphingomonadales; f__Sphingomonadaceae; g__Sphingomonas; s__
360432	0.9276	0.0045	k__Bacteria; p__Proteobacteria; c__Alphaproteobacteria; o__Rhodobacterales; f__Rhodobacteraceae; g__Rubellimicrobium; s__
854272	0.9255	0.0049	k__Bacteria; p__Proteobacteria; c__Alphaproteobacteria; o__Rhodobacterales; f__Rhodobacteraceae; g__; s__
822853	0.8986	0.0112	k__Bacteria; p__Proteobacteria; c__Alphaproteobacteria; o__Sphingomonadales; f__Sphingomonadaceae; g__; s__
4479555	0.8986	0.0112	k__Bacteria; p__Proteobacteria; c__Alphaproteobacteria; o__Rhodospirillales; f__Acetobacteraceae
249034	0.8933	0.0128	k__Bacteria; p__Proteobacteria; c__Alphaproteobacteria; o__; f__; g__; s__
544841	0.8933	0.0128	k__Bacteria; p__Proteobacteria; c__Alphaproteobacteria; o__Sphingomonadales; f__Sphingomonadaceae; g__Sphingomonas; s__
2994818	0.8933	0.0128	k__Bacteria; p__Proteobacteria; c__Alphaproteobacteria; o__Sphingomonadales; f__; g__; s__
New.ReferenceOTU40	0.8933	0.0128	k__Bacteria; p__Proteobacteria; c__Alphaproteobacteria; o__Sphingomonadales; f__Sphingomonadaceae; g__; s__
New.CleanUp.ReferenceOTU206	0.8933	0.0128	k__Bacteria; p__Proteobacteria; c__Alphaproteobacteria; o__; f__; g__; s__
New.CleanUp.ReferenceOTU9578	0.8933	0.0128	k__Bacteria; p__Proteobacteria; c__Alphaproteobacteria; o__Sphingomonadales; f__Sphingomonadaceae; g__; s__
New.CleanUp.ReferenceOTU17329	0.8933	0.0128	k__Bacteria; p__Proteobacteria; c__Alphaproteobacteria; o__Caulobacterales; f__Caulobacteraceae; g__; s__
593441	0.8676	0.0219	k__Bacteria; p__Proteobacteria; c__Alphaproteobacteria; o__Rhodobacterales; f__Rhodobacteraceae; g__Anaerospora; s__
New.CleanUp.ReferenceOTU19464	0.8676	0.0219	k__Bacteria; p__Proteobacteria; c__Alphaproteobacteria; o__Sphingomonadales; f__Sphingomonadaceae; g__Novosphingobium; s__
4404	0.8575	0.0262	k__Bacteria; p__Proteobacteria; c__Alphaproteobacteria; o__Rhodospirillales; f__Acetobacteraceae; g__Roseococcus; s__
619602	0.8575	0.0262	k__Bacteria; p__Proteobacteria; c__Alphaproteobacteria; o__Rhodobacterales; f__Rhodobacteraceae; g__Rhodobacter; s__
New.CleanUp.ReferenceOTU369	0.8575	0.0262	k__Bacteria; p__Proteobacteria; c__Alphaproteobacteria; o__Sphingomonadales; f__Sphingomonadaceae
New.CleanUp.ReferenceOTU1529	0.8575	0.0262	k__Bacteria; p__Proteobacteria; c__Alphaproteobacteria; o__Rickettsiales; f__mitochondria; g__Vermamoeba; s__vermiformis
New.CleanUp.ReferenceOTU3450	0.8575	0.0262	k__Bacteria; p__Proteobacteria; c__Alphaproteobacteria; o__Sphingomonadales; f__; g__; s__
New.CleanUp.ReferenceOTU6988	0.8575	0.0262	k__Bacteria; p__Proteobacteria; c__Alphaproteobacteria; o__; f__; g__; s__
New.CleanUp.ReferenceOTU10038	0.8575	0.0262	k__Bacteria; p__Proteobacteria; c__Alphaproteobacteria; o__Rhodobacterales; f__Rhodobacteraceae; g__Rhodobacter; s__
New.CleanUp.ReferenceOTU11870	0.8575	0.0262	k__Bacteria; p__Proteobacteria; c__Alphaproteobacteria; o__Rhodospirillales; f__Acetobacteraceae; g__Roseococcus; s__
New.CleanUp.ReferenceOTU15305	0.8575	0.0262	k__Bacteria; p__Proteobacteria; c__Alphaproteobacteria; o__Rhodospirillales; f__Acetobacteraceae; g__; s__
807415	0.8508	0.0292	k__Bacteria; p__Proteobacteria; c__Alphaproteobacteria; o__Rhodobacterales; f__Rhodobacteraceae; g__Rhodobacter; s__
147648	0.8454	0.0318	k__Bacteria; p__Proteobacteria; c__Alphaproteobacteria; o__Sphingomonadales; f__Sphingomonadaceae; g__; s__
New.CleanUp.ReferenceOTU15962	0.8454	0.0318	k__Bacteria; p__Proteobacteria; c__Alphaproteobacteria; o__Sphingomonadales; f__Sphingomonadaceae; g__Zymomonas; s__
4408928	0.8402	0.0343	k__Bacteria; p__Proteobacteria; c__Alphaproteobacteria; o__Sphingomonadales; f__Sphingomonadaceae; g__Novosphingobium; s__
New.CleanUp.ReferenceOTU1602	0.8402	0.0343	k__Bacteria; p__Proteobacteria; c__Alphaproteobacteria; o__Sphingomonadales; f__Sphingomonadaceae; g__Kaistobacter; s__
New.CleanUp.ReferenceOTU6333	0.8402	0.0343	k__Bacteria; p__Proteobacteria; c__Alphaproteobacteria; o__Caulobacterales; f__Caulobacteraceae; g__Mycoplana; s__
New.CleanUp.ReferenceOTU10749	0.8402	0.0343	k__Bacteria; p__Proteobacteria; c__Alphaproteobacteria; o__Rhodobacterales; f__Rhodobacteraceae; g__Rhodobacter; s__
New.CleanUp.ReferenceOTU12783	0.8402	0.0343	k__Bacteria; p__Proteobacteria; c__Alphaproteobacteria; o__Sphingomonadales; f__Sphingomonadaceae; g__Kaistobacter; s__
New.CleanUp.ReferenceOTU16203	0.8402	0.0343	k__Bacteria; p__Proteobacteria; c__Alphaproteobacteria; o__Rhodospirillales; f__Acetobacteraceae; g__Roseococcus; s__
559211	-0.8317	0.0387	k__Bacteria; p__Proteobacteria; c__Alphaproteobacteria; o__Rhizobiales; f__Bradyrhizobiaceae; g__; s__

Table S7 Supplementary Material. Microbialite Cobalt content Spearman test (OTU level) results, cutoff= Spearman rho > 0.8, p <0.05

OTU ID	Spearman rho	p value	taxonomy
572889	0.9798	0.0001	k__Bacteria; p__Fusobacteria; c__Fusobacteriia; o__Fusobacteriales; f__Fusobacteriaceae; g__Fusobacterium; s__
New.CleanUp.ReferenceOTU18131	0.9798	0.0001	k__Bacteria; p__Actinobacteria; c__Actinobacteria; o__Actinomycetales; f__Nakamurellaceae; g__; s__
1121806	-0.8764	0.0185	k__Bacteria; p__Chloroflexi; c__Gitt-GS-136; o__; f__; g__; s__
233981	-0.8764	0.0185	k__Bacteria; p__Proteobacteria; c__Gammaproteobacteria; o__Enterobacteriales; f__Enterobacteriaceae
560709	-0.8485	0.0303	k__Bacteria; p__Proteobacteria; c__Gammaproteobacteria; o__Pasteurellales; f__Pasteurellaceae; g__Aggregatibacter; s__
818188	-0.8452	0.0319	k__Bacteria; p__Cyanobacteria; c__Synechococcophycideae; o__Pseudanabaenales; f__Pseudanabaenaceae; g__; s__
839235	-0.8452	0.0319	k__Bacteria; p__Proteobacteria; c__Gammaproteobacteria; o__Aeromonadales; f__Aeromonadaceae; g__; s__
922761	-0.8452	0.0319	k__Bacteria; p__Proteobacteria; c__Gammaproteobacteria; o__Enterobacteriales; f__Enterobacteriaceae; g__; s__
1032653	-0.8356	0.0367	k__Bacteria; p__Actinobacteria; c__Thermoleophilia; o__Solirubrobacterales; f__Solirubrobacteraceae; g__; s__
129048	-0.8356	0.0367	k__Bacteria; p__Cyanobacteria; c__Chloroplast; o__Streptophyta; f__; g__; s__
3114957	-0.8356	0.0367	k__Bacteria; p__Chloroflexi; c__Chloroflexi; o__[Roseiflexales]; f__; g__; s__
1823053	-0.8356	0.0367	k__Bacteria; p__Firmicutes; c__Bacilli; o__Lactobacillales; f__Streptococcaceae; g__Lactococcus; s__
654742	-0.8356	0.0367	k__Bacteria; p__Proteobacteria; c__Alphaproteobacteria; o__Sphingomonadales; f__Sphingomonadaceae; g__Kaistobacter; s__
797560	-0.8356	0.0367	k__Bacteria; p__Firmicutes; c__Bacilli; o__Lactobacillales; f__Streptococcaceae; g__Streptococcus; s__
854272	-0.8356	0.0367	k__Bacteria; p__Proteobacteria; c__Alphaproteobacteria; o__Rhodobacterales; f__Rhodobacteraceae; g__; s__
New.ReferenceOTU91	-0.8356	0.0367	k__Bacteria; p__Chloroflexi; c__Chloroflexi; o__Chloroflexales; f__Oscillochloridaceae; g__Oscillochloris
New.CleanUp.ReferenceOTU6668	-0.8356	0.0367	k__Bacteria; p__Verrucomicrobia; c__[Spartobacteria]; o__[Chthoniobacterales]; f__[Chthoniobacteraceae]; g__Candidatus Xiphinematobacter; s__
859313	-0.8232	0.0433	k__Bacteria; p__Actinobacteria; c__Acidimicrobiia; o__Acidimicrobiales; f__C111; g__; s__
873518	-0.8232	0.0433	k__Bacteria; p__Proteobacteria; c__Deltaproteobacteria; o__Myxococcales; f__; g__; s__
927623	-0.8232	0.0433	k__Bacteria; p__Bacteroidetes; c__Cytophagia; o__Cytophagales; f__Cytophagaceae; g__Hymenobacter; s__
189028	-0.8232	0.0433	k__Bacteria; p__Cyanobacteria; c__Synechococcophycideae; o__Pseudanabaenales; f__Pseudanabaenaceae; g__; s__
1117222	-0.8232	0.0433	k__Bacteria; p__Bacteroidetes; c__Flavobacteriia; o__Flavobacteriales; f__Flavobacteriaceae; g__Flavobacterium; s__
1141758	-0.8232	0.0433	k__Bacteria; p__Cyanobacteria; c__Chloroplast; o__Streptophyta; f__; g__; s__
217746	-0.8232	0.0433	k__Bacteria; p__Chloroflexi; c__Ellin6529; o__; f__; g__; s__
35188	-0.8232	0.0433	k__Bacteria; p__Cyanobacteria; c__Oscillatoriophycideae; o__Oscillatoriales; f__Phormidiaceae; g__Phormidium; s__
3077248	-0.8232	0.0433	k__Bacteria; p__Proteobacteria; c__Alphaproteobacteria; o__Rhizobiales; f__Rhodobiaceae; g__Afifella; s__
107234	-0.8232	0.0433	k__Archaea; p__Crenarchaeota; c__Thaumarchaeota; o__Nitrososphaerales; f__Nitrososphaeraceae; g__Candidatus Nitrososphaera; s__SCA1145
495084	-0.8232	0.0433	k__Bacteria; p__Firmicutes; c__Clostridia; o__Clostridiales; f__[Tissierellaceae]; g__Anaerococcus; s__
150955	-0.8232	0.0433	k__Bacteria; p__Bacteroidetes; c__Cytophagia; o__Cytophagales; f__Cytophagaceae; g__Hymenobacter; s__
809485	-0.8232	0.0433	k__Bacteria; p__Bacteroidetes; c__Cytophagia; o__Cytophagales; f__Cytophagaceae; g__; s__
New.CleanUp.ReferenceOTU15689	-0.8232	0.0433	k__Bacteria; p__Bacteroidetes; c__Flavobacteriia; o__Flavobacteriales; f__Flavobacteriaceae; g__Flavobacterium; s__
New.CleanUp.ReferenceOTU17024	-0.8232	0.0433	k__Bacteria; p__Firmicutes; c__Clostridia; o__Clostridiales; f__[Tissierellaceae]; g__Helcococcus; s__
New.CleanUp.ReferenceOTU19016	-0.8232	0.0433	k__Bacteria; p__Proteobacteria; c__Alphaproteobacteria; o__Rhodobacterales; f__Rhodobacteraceae; g__Rubellimicrobium; s__
1116186	0.8216	0.0442	k__Bacteria; p__Chloroflexi; c__Anaerolineae; o__Caldilineales; f__Caldilineaceae; g__; s__

Table S8 Supplementary Material. Microbialite Cadmium content Spearman test (OTU level) results, cutoff= Spearman rho > 0.91, p <0.008

OTU ID	Spearman rho	p value	taxonomy
150955	-0.9856	0.0000	k__Bacteria; p__Bacteroidetes; c__Cytophagia; o__Cytophagales; f__Cytophagaceae; g__Hymenobacter; s__
New.CleanUp.ReferenceOTU15689	-0.9856	0.0000	k__Bacteria; p__Bacteroidetes; c__Flavobacteriia; o__Flavobacteriales; f__Flavobacteriaceae; g__Flavobacterium; s__
797560	-0.9710	0.0003	k__Bacteria; p__Firmicutes; c__Bacilli; o__Lactobacillales; f__Streptococcaceae; g__Streptococcus; s__
904882	-0.9411	0.0025	k__Bacteria; p__Bacteroidetes; c__Cytophagia; o__Cytophagales; f__Cytophagaceae; g__Hymenobacter; s__
159973	-0.9411	0.0025	k__Bacteria; p__Bacteroidetes; c__Cytophagia; o__Cytophagales; f__Cytophagaceae; g__Hymenobacter; s__
312035	-0.9411	0.0025	k__Bacteria; p__Cyanobacteria; c__Nostocophycideae; o__Nostocales; f__Nostocaceae; g__Nostoc; s__
645729	-0.9411	0.0025	k__Bacteria; p__Proteobacteria; c__Alphaproteobacteria; o__Sphingomonadales; f__Sphingomonadaceae; g__; s__
821737	-0.9411	0.0025	k__Bacteria; p__Cyanobacteria; c__Synechococcophycideae; o__Pseudanabaenales; f__Pseudanabaenaceae; g__Leptolyngbya; s__
2228264	-0.9411	0.0025	k__Bacteria; p__Proteobacteria; c__Alphaproteobacteria; o__Rhodobacterales; f__Rhodobacteraceae; g__Rubellimicrobium; s__
540464	-0.9411	0.0025	k__Bacteria; p__Verrucomicrobia; c__Verrucomicrobiae; o__Verrucomicrobiales; f__Verrucomicrobiaceae; g__Luteolibacter; s__
836522	-0.9411	0.0025	k__Bacteria; p__Bacteroidetes; c__Sphingobacteriia; o__Sphingobacteriales; f__Sphingobacteriaceae; g__; s__
New.CleanUp.ReferenceOTU3070	-0.9411	0.0025	k__Bacteria; p__Proteobacteria; c__Alphaproteobacteria; o__Sphingomonadales; f__Sphingomonadaceae; g__; s__
New.CleanUp.ReferenceOTU3432	-0.9411	0.0025	k__Bacteria; p__Bacteroidetes; c__Cytophagia; o__Cytophagales; f__Cytophagaceae; g__Hymenobacter; s__
New.CleanUp.ReferenceOTU3437	-0.9411	0.0025	k__Bacteria; p__Bacteroidetes; c__Sphingobacteriia; o__Sphingobacteriales; f__Sphingobacteriaceae; g__; s__
New.CleanUp.ReferenceOTU4376	-0.9411	0.0025	Unassigned
New.CleanUp.ReferenceOTU4511	-0.9411	0.0025	k__Bacteria; p__Proteobacteria; c__Alphaproteobacteria; o__Rhodobacterales; f__Rhodobacteraceae
New.CleanUp.ReferenceOTU7534	-0.9411	0.0025	Unassigned
New.CleanUp.ReferenceOTU10674	-0.9411	0.0025	k__Bacteria; p__Proteobacteria; c__Deltaproteobacteria; o__; f__; g__; s__
New.CleanUp.ReferenceOTU14798	-0.9411	0.0025	k__Bacteria; p__Proteobacteria; c__Alphaproteobacteria; o__BD7-3; f__; g__; s__
New.CleanUp.ReferenceOTU16846	-0.9411	0.0025	k__Bacteria; p__Bacteroidetes; c__Flavobacteriia; o__Flavobacteriales; f__Flavobacteriaceae; g__Flavobacterium; s__
New.CleanUp.ReferenceOTU17531	-0.9411	0.0025	k__Bacteria; p__Proteobacteria; c__Deltaproteobacteria; o__Spirobacillales; f__; g__; s__
873518	-0.9276	0.0045	k__Bacteria; p__Proteobacteria; c__Deltaproteobacteria; o__Myxococcales; f__; g__; s__
1117222	-0.9276	0.0045	k__Bacteria; p__Bacteroidetes; c__Flavobacteriia; o__Flavobacteriales; f__Flavobacteriaceae; g__Flavobacterium; s__
New.CleanUp.ReferenceOTU19016	-0.9276	0.0045	k__Bacteria; p__Proteobacteria; c__Alphaproteobacteria; o__Rhodobacterales; f__Rhodobacteraceae; g__Rubellimicrobium; s__
111797	-0.9258	0.0048	k__Bacteria; p__Proteobacteria; c__Alphaproteobacteria; o__Rhizobiales
2603	-0.9258	0.0048	k__Bacteria; p__Bacteroidetes; c__Cytophagia; o__Cytophagales; f__Cytophagaceae; g__Hymenobacter; s__
886673	-0.9258	0.0048	k__Bacteria; p__Bacteroidetes; c__Flavobacteriia; o__Flavobacteriales; f__Flavobacteriaceae; g__Flavobacterium; s__
New.CleanUp.ReferenceOTU5811	-0.9258	0.0048	k__Bacteria; p__Proteobacteria; c__Alphaproteobacteria; o__Sphingomonadales; f__Sphingomonadaceae; g__; s__
New.CleanUp.ReferenceOTU10170	-0.9258	0.0048	k__Bacteria; p__Proteobacteria; c__Alphaproteobacteria; o__Sphingomonadales; f__Sphingomonadaceae; g__; s__
New.CleanUp.ReferenceOTU10344	-0.9258	0.0048	k__Bacteria; p__Proteobacteria; c__Alphaproteobacteria; o__Rhodobacterales; f__Rhodobacteraceae; g__Rubellimicrobium; s__
New.CleanUp.ReferenceOTU12521	-0.9258	0.0048	k__Bacteria; p__Bacteroidetes; c__Sphingobacteriia; o__Sphingobacteriales; f__Sphingobacteriaceae; g__; s__
New.CleanUp.ReferenceOTU13057	-0.9258	0.0048	Unassigned
New.CleanUp.ReferenceOTU15704	-0.9258	0.0048	k__Bacteria; p__Bacteroidetes; c__Flavobacteriia; o__Flavobacteriales; f__Flavobacteriaceae; g__Flavobacterium; s__
New.CleanUp.ReferenceOTU16485	-0.9258	0.0048	k__Bacteria; p__Proteobacteria; c__Alphaproteobacteria; o__Rhodobacterales; f__Rhodobacteraceae; g__Rubellimicrobium; s__
New.CleanUp.ReferenceOTU18068	-0.9258	0.0048	k__Bacteria; p__Proteobacteria; c__Deltaproteobacteria; o__Bdellovibrionales; f__Bacteriovoracaceae; g__; s__
854272	-0.9122	0.0076	k__Bacteria; p__Proteobacteria; c__Alphaproteobacteria; o__Rhodobacterales; f__Rhodobacteraceae; g__; s__

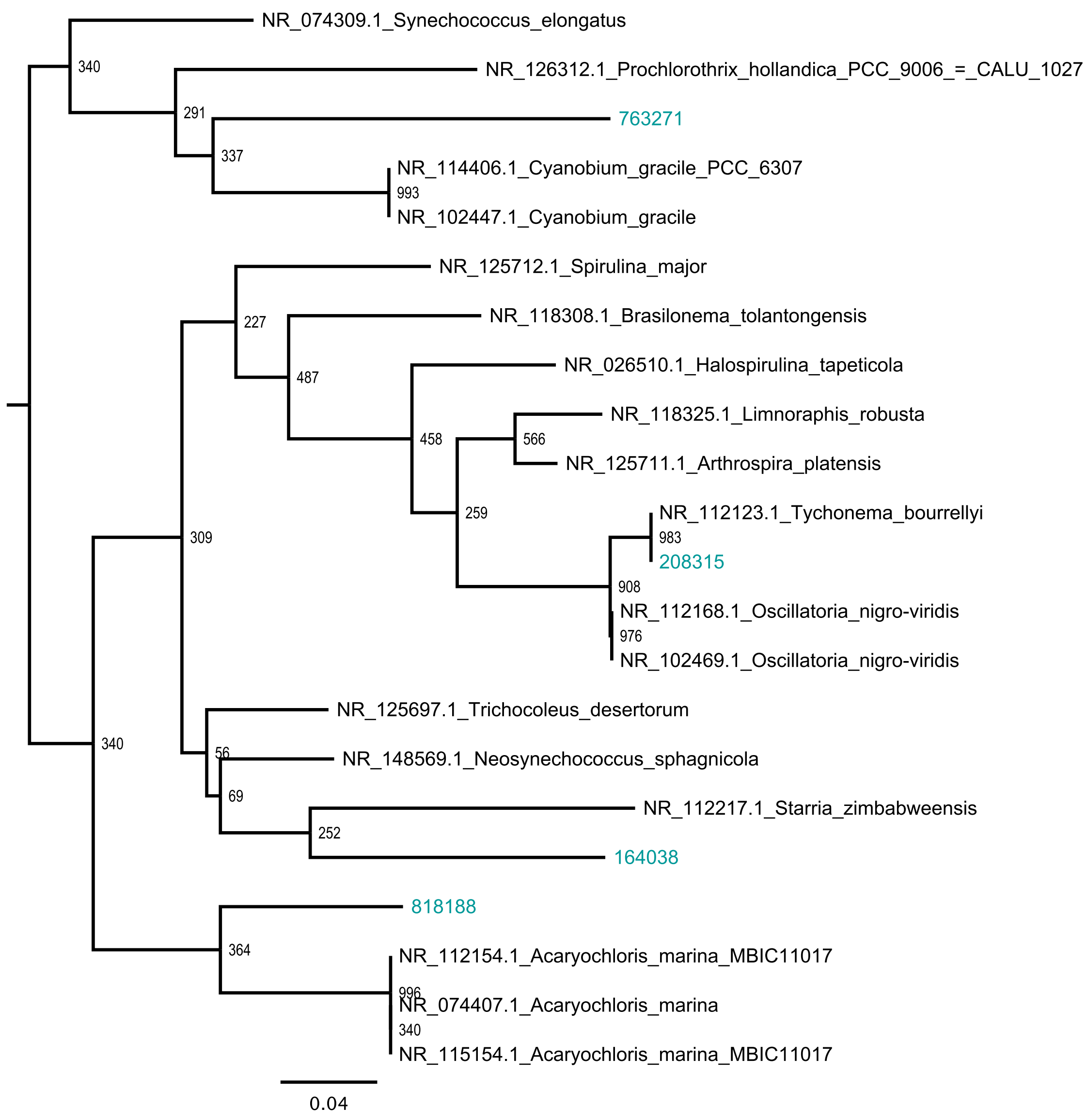

	Greengenes 13.5		Closest taxonomic affiliation (Silva 128 Database)		
OTU ID	Genus	Order	Species	Order	Habitat, features (NCBI)
164038	*Leptolyngbya*	Synechococcales	*Starria zimbabweënsis*	Oscillatoriales	
818188		Synechococcales	*Acharyochloris marina*	Synechococcales	Coral holobiont, produces chlorophyll d
208315		Oscillatoriales	*Tychonema bourrellyi*	Oscillatoriales	Pelagic freshwater, anatoxin-a production
763271	*Leptolyngbya*	Synechococcales	*Synechococcus elongatus*	Synechococcales	

Figure S2 Supplementary Material. Taxonomic affiliation exploration of shared (shared at least in four microbialites) cyanobacterial OTUs, using refseq Database, NCBI (tree construction: GTR model, Maximum likelihood, 1000 bootstrap). Microbialite OTU IDs are written in turquoise color.
