# Supplementary material for: Exploring Biogeochemistry and Microbial Diversity of Extant Microbialites in Mexico and Cuba
Source: Front Microbiol. 2018 Apr 3;9:510. doi: 10.3389/fmicb.2018.00510 (PMC5891642; doi:10.3389/fmicb.2018.00510)
Supplement: Supplementary file 2 [file DataSheet2.PDF]

Table S9 Supplementary Material. Elemental chemistry of microbialites, average and standard deviation of XRF triplicate measurements and elemental analyses (C_{org} and N_{total}) quintuplicate measurements of microbialites from PAI (Pozas Azules I, Cuatro Ciénegas Basin); AS and AC (Alchichica crater lake); BAC (Bacalar Lagoon); MU (Muyil Lagoon, Quintana Roo) and CU (Cayo Sabinal, Northern Keys, Cuba).

Element	Na	Na	Mg	Mg	Al	Al	Si	Si	P	P	S	S	Cl	Cl	K	K
Dimension	%	rror (%)	%	rror (%)	%	rror (%)	%	rror (%)	%	rror (%)	%	rror (%)	%	rror (%)	%	rror (%)
(1) AC	0.397	0.015	1.861	0.015	0.044	0.002	2.687	0.004	0.050	0.000	0.225	0.000	0.008	0.000	0.068	0.002
(2) AC	0.429	0.016	1.686	0.015	0.033	0.002	2.496	0.003	0.047	0.000	0.202	0.000	0.012	0.000	0.066	0.002
(3) AC	0.389	0.015	1.743	0.015	0.068	0.002	2.713	0.004	0.048	0.000	0.215	0.000	0.009	0.000	0.069	0.002
Average	0.405		1.763		0.048		2.632		0.048		0.214		0.010		0.068	
Standard deviation	0.021		0.089		0.018		0.118		0.001		0.011		0.002		0.002	
(1) AS	< 0.010	0.000	13.870	0.020	0.809	0.005	1.913	0.002	0.042	0.000	0.103	0.000	0.227	0.000	0.135	0.001
(2) AS	< 0.010	0.000	12.350	0.020	0.709	0.005	1.777	0.002	0.040	0.000	0.084	0.000	0.152	0.000	0.129	0.001
(3) AS	< 0.010	0.000	12.530	0.020	0.813	0.005	1.870	0.002	0.041	0.000	0.097	0.000	0.183	0.000	0.134	0.001
Average			12.917		0.777		1.853		0.041		0.095		0.187		0.132	
Standard deviation			0.831		0.059		0.070		0.001		0.009		0.038		0.003	
(1) BAC	0.251	0.010	0.869	0.014	< 0.0020	0.000	0.100	0.001	0.013	0.000	1.173	0.001	0.00020	0.000	0.019	0.002
(2) BAC	0.529	0.020	0.841	0.015	< 0.0020	0.000	0.122	0.002	0.012	0.000	1.136	0.001	0.00020	0.000	0.018	0.002
(3) BAC	0.372	0.015	0.832	0.014	< 0.0020	0.000	0.128	0.002	0.012	0.000	1.137	0.001	0.00020	0.000	0.020	0.002
Average	0.384		0.847				0.117		0.013		1.149				0.019	
Standard deviation	0.139		0.019				0.015		0.000		0.021				0.001	
(1) MU	< 0.010	0.000	1.208	0.012	< 0.0020	0.000	0.005	0.000	0.014	0.000	0.209	0.000	0.007	0.000	0.019	0.002
(2) MU	< 0.010	0.000	1.269	0.013	< 0.0020	0.000	0.010	0.000	0.013	0.000	0.203	0.000	0.00020	0.000	0.016	0.002
(3) MU	< 0.010	0.000	1.334	0.013	0.041	0.002	0.133	0.001	0.013	0.000	0.221	0.000	0.00020	0.000	0.019	0.002
Average			1.270				0.049		0.013		0.211				0.018	
Standard deviation			0.063				0.072		0.001		0.009				0.002	
(1) PAI	0.734	0.026	0.937	0.015	0.029	0.003	1.779	0.003	0.013	0.000	1.154	0.001	0.014	0.000	0.035	0.002
(2) PAI	0.549	0.021	0.865	0.014	< 0.0020	0.000	1.512	0.003	0.013	0.000	1.107	0.001	0.011	0.000	0.033	0.002
(3) PAI	0.589	0.022	0.867	0.014	0.005	0.003	1.579	0.003	0.013	0.000	1.102	0.001	0.009	0.000	0.030	0.002
Average	0.624		0.890		0.017		1.623		0.013		1.121		0.011		0.033	
Standard deviation	0.097		0.041		0.017		0.139		0.000		0.029		0.002		0.002	
(1) CU	20.950	0.440	4.709	0.084	0.011	0.011	0.220	0.004	0.030	0.001	2.113	0.002	19.930	0.010	0.466	0.002
(2) CU	14.970	0.330	4.395	0.058	0.069	0.014	0.230	0.004	0.030	0.000	2.264	0.002	12.850	0.010	0.439	0.002
(3) CU	16.150	0.340	4.675	0.061	0.096	0.014	0.302	0.004	0.030	0.000	2.304	0.002	13.040	0.010	0.443	0.002
Average	17.357		4.593		0.059		0.250		0.030		2.227		15.273		0.449	
Standard deviation	3.167		0.172		0.043		0.045		0.000		0.101		4.034		0.015	

Element	Ca	Ca	V	V	Cr	Cr	Fe	Fe	Co	Co	Ni	Ni	Cu	Cu	As	As	Se	Se
Dimension	%	rror (%)	µg/g	or (µg/g)	µg/g	or (µg/g)	%	rror (%)	µg/g	or (µg/g)	µg/g	or (µg/g)	µg/g	or (µg/g)	µg/g	or (µg/g)	µg/g	or (µg/g)
(1) AC	29.250	0.020	10.200	2.400	7.500	0.600	0.00010	0.000	< 3.0	0.000	7.900	0.700	15.300	0.800	17.900	0.300	< 0.5	-0.500
(2) AC	29.070	0.020	14.400	2.400	8.400	0.600	0.00010	0.000	3.600	2.000	13.500	0.800	15.100	0.800	17.300	0.400	0.500	0.100
(3) AC	29.170	0.020	14.400	2.400	8.500	0.600	0.00010	0.000	< 3.0	0.000	14.000	0.800	15.700	0.800	17.400	0.400	< 0.5	-0.400
Average	29.163		13.000		8.133						11.800		15.367		17.533			
Standard deviation	0.090		2.425		0.551						3.387		0.306		0.321			
(1) AS	6.875	0.005	7.700	1.500	11.900	0.600	1.769	0.004	< 3.0	-2.000	19.300	0.700	27.200	0.800	7.700	0.200	< 0.5	0.000
(2) AS	6.920	0.005	9.200	1.500	13.100	0.600	1.666	0.003	< 3.0	0.000	28.600	0.800	26.100	0.800	7.500	0.200	< 0.5	0.000
(3) AS	6.971	0.005	11.800	1.500	14.300	0.600	1.710	0.004	< 3.0	-1.800	25.900	0.800	27.100	0.800	7.200	0.200	< 0.5	0.000
Average	6.922		9.567		13.100		1.715				24.600		26.800		7.467			
Standard deviation	0.048		2.074		1.200		0.052				4.784		0.608		0.252			
(1) BAC	25.470	0.020	9.400	0.900	6.600	0.400	0.00010	0.000	< 3.0	-1.300	6.100	0.500	15.600	0.800	< 0.5	0.000	0.900	0.100
(2) BAC	25.670	0.020	11.900	0.900	6.500	0.400	0.00010	0.000	< 3.0	0.000	8.100	0.500	15.400	0.800	< 0.5	0.000	0.800	0.100
(3) BAC	25.620	0.020	10.700	0.900	6.800	0.400	0.00010	0.000	< 3.0	0.000	8.500	0.500	15.900	0.800	< 0.5	0.000	0.900	0.100
Average	25.587		10.667		6.633						7.567		15.633				0.867	
Standard deviation	0.104		1.250		0.153						1.286		0.252				0.058	
(1) MU	30.000	0.020	10.600	0.900	5.400	0.400	0.00010	0.000	7.100	2.100	5.800	0.500	15.200	0.700	< 0.5	0.000	0.700	0.100
(2) MU	31.480	0.020	9.100	1.000	5.500	0.400	0.00010	0.000	4.300	2.200	8.800	0.500	14.600	0.700	< 0.5	0.000	0.600	0.100
(3) MU	31.340	0.020	9.900	1.000	6.300	0.400	0.00010	0.000	3.100	2.200	7.400	0.500	15.400	0.700	< 0.5	0.000	< 0.5	-0.500
Average	30.940		9.867		5.733				4.833		7.333		15.067				0.650	
Standard deviation	0.817		0.751		0.493				2.053		1.501		0.416				0.071	
(1) PAI	23.690	0.010	9.400	0.900	5.800	0.400	0.00010	0.000	3.100	2.200	6.900	0.500	6.500	0.300	2.600	0.200	0.900	0.100
(2) PAI	24.010	0.010	8.200	0.900	5.600	0.400	0.00010	0.000	3.700	2.300	10.700	0.500	8.300	0.400	2.100	0.200	0.900	0.100
(3) PAI	24.150	0.010	9.100	0.900	5.800	0.400	0.00010	0.000	< 3.0	-2.100	10.200	0.500	6.400	0.300	2.700	0.200	0.700	0.100
Average	23.950		8.900		5.733				3.400		9.267		7.067		2.467		0.833	
Standard deviation	0.236		0.624		0.115				0.424		2.065		1.069		0.321		0.115	
(1) CU	6.495	0.004	12.900	1.000	11.300	0.500	0.028	0.000	< 3.0	0.000	13.200	0.600	20.600	0.700	< 0.5	0.000	< 0.5	-0.500
(2) CU	7.470	0.005	12.500	1.000	10.500	0.500	0.030	0.000	< 3.0	-2.800	18.600	0.600	20.200	0.700	< 0.5	0.000	< 0.5	-0.500
(3) CU	7.470	0.005	11.400	1.000	10.900	0.500	0.031	0.000	< 3.0	-1.700	16.400	0.600	20.400	0.700	< 0.5	0.000	< 0.5	-0.400
Average	7.145		12.267		10.900		0.029				16.067		20.400					
Standard deviation	0.563		0.777		0.400		0.002				2.715		0.200					

Element	Rb	Rb	Sr	Sr	Mo	Mo	Ag	Ag	Cd	Cd	Pb	Pb	Bi	Bi	Th	Th	U	U
Dimension	µg/g	or (µg/g)	µg/g	or (µg/g)	µg/g	or (µg/g)	µg/g	or (µg/g)	µg/g	or (µg/g)	µg/g	or (µg/g)	µg/g	or (µg/g)	µg/g	or (µg/g)	µg/g	or (µg/g)
(1) AC	1.800	0.300	3057.000	3.000	1.700	0.400	< 2.0	0.000	4.700	1.400	< 1.0	0.000	2.000	0.500	< 1.0	0.000	< 1.0	-0.400
(2) AC	1.800	0.300	3065.000	3.000	1.400	0.400	< 2.0	0.000	2.300	1.200	< 1.0	0.000	1.900	0.500	< 1.0	0.000	< 1.0	-0.700
(3) AC	1.900	0.300	3084.000	3.000	1.400	0.400	< 2.0	0.000	4.400	1.400	< 1.0	-0.300	2.100	0.500	< 1.0	0.000	< 1.0	0.000
Average	1.833		3068.667		1.500				3.800				2.000					
Standard deviation	0.058		13.868		0.173				1.308				0.100					
(1) AS	5.700	0.200	1126.000	1.000	2.100	0.300	< 2.0	0.000	< 2.0	0.000	< 1.0	-0.300	< 1.0	0.000	< 1.0	0.000	< 1.0	-0.800
(2) AS	6.300	0.200	1123.000	1.000	1.600	0.300	9.000	1.800	2.500	0.900	1.300	0.300	< 1.0	0.000	< 1.0	0.000	< 1.0	0.000
(3) AS	6.400	0.200	1151.000	1.000	2.100	0.300	8.100	1.900	< 2.0	-2.000	1.800	0.300	< 1.0	0.000	< 1.0	0.000	< 1.0	0.000
Average	6.133		1133.333		1.933		8.550		2.500		1.550							
Standard deviation	0.379		15.373		0.289		0.636				0.354							
(1) BAC	0.900	0.300	2230.000	2.000	2.400	0.400	< 2.0	0.000	3.100	1.500	< 1.0	0.000	2.400	0.400	< 1.0	0.000	< 1.0	0.000
(2) BAC	0.600	0.300	2268.000	2.000	1.900	0.400	< 2.0	0.000	8.200	1.800	< 1.0	0.000	2.300	0.400	< 1.0	0.000	< 1.0	-0.500
(3) BAC	< 0.5	-0.500	2259.000	2.000	2.000	0.400	< 2.0	0.000	3.400	1.500	< 1.0	0.000	2.800	0.500	< 1.0	0.000	< 1.0	0.000
Average	0.750		2252.333		2.100				4.900				2.500					
Standard deviation	0.212		19.858		0.265				2.862				0.265					
(1) MU	< 0.5	0.000	1390.000	1.000	3.300	0.400	< 2.0	0.000	7.600	1.700	< 1.0	0.000	3.000	0.400	< 1.0	0.000	< 1.0	0.000
(2) MU	< 0.5	0.000	1488.000	1.000	3.400	0.400	< 2.0	0.000	4.300	1.700	< 1.0	0.000	3.200	0.400	< 1.0	0.000	< 1.0	-0.800
(3) MU	< 0.5	-0.100	1486.000	1.000	3.300	0.400	< 2.0	0.000	7.100	1.800	< 1.0	-0.200	1.800	0.400	< 1.0	0.000	< 1.0	0.000
Average			1454.667		3.333				6.333				2.667					
Standard deviation			56.012		0.058				1.779				0.757					
(1) PAI	1.300	0.400	3923.000	3.000	< 1.0	-0.900	< 2.0	0.000	3.100	1.400	< 1.0	-0.500	2.300	0.500	< 1.0	0.000	< 1.0	0.000
(2) PAI	2.100	0.400	3979.000	3.000	< 1.0	0.000	< 2.0	0.000	7.100	1.700	1.800	0.200	2.800	0.500	< 1.0	0.000	< 1.0	-1.000
(3) PAI	1.000	0.400	3990.000	3.000	< 1.0	-0.500	< 2.0	0.000	6.500	1.600	< 1.0	-1.000	2.900	0.500	< 1.0	0.000	1.300	0.100
Average	1.467		3964.000						5.567				2.667				1.300	
Standard deviation	0.569		35.930						2.157				0.321					
(1) CU	< 0.5	0.000	1205.000	1.000	2.900	0.300	< 2.0	0.000	2.700	0.900	4.900	0.300	2.400	0.400	5.600	0.400	4.300	0.200
(2) CU	< 0.5	0.000	1275.000	1.000	2.700	0.300	< 2.0	0.000	< 2.0	-1.500	4.700	0.300	2.900	0.400	3.900	0.400	3.800	0.200
(3) CU	< 0.5	0.000	1281.000	1.000	2.500	0.300	< 2.0	0.000	< 2.0	-1.600	5.100	0.300	2.600	0.400	4.700	0.400	3.600	0.200
Average			1253.667		2.700				2.700		4.900		2.633		4.733		3.900	
Standard deviation			42.253		0.200						0.200		0.252		0.850		0.361	

Element	Corg	N
Dimension	mg/g	mg/g
(1) AC	18.12	2.31
(2) AC	19.73	2.59
(3) AC	8.52	1.06
(4) AC	15.16	1.77
(5) AC	25.15	2.82
(6) AC	31.26	2.99
Average	19.66	2.26
Standard deviation	7.89	0.72
(1) AS	11.28	1.63
(2) AS	16.28	2.81
(3) AS	20.86	2.70
(4) AS	9.49	1.26
(5) AS	29.51	4.09
(6) AS	32.11	4.99
Average	19.92	2.92
Standard deviation	9.36	1.42
(1) BAC	36.40	3.80
(2) BAC	85.99	3.56
(3) BAC	28.62	2.55
(4) BAC	48.77	1.87
(5) BAC	34.55	3.88
(6) BAC	32.33	3.64
Average	44.44	3.22
Standard deviation	21.47	0.82
(1) MU	15.09	1.75
(2) MU	17.55	2.07
(3) MU	18.80	2.16
(4) MU	19.46	2.32
(5) MU	21.56	2.33
(6) MU	14.57	1.67
(7) MU	24.33	2.46
(8) MU	14.71	1.39
(9) MU	22.54	2.41
(10) MU	19.18	2.05
Average	18.78	2.06
Standard deviation	3.38	0.36
(1) PAI	11.25	1.46
(2) PAI	15.62	2.10
(3) PAI	11.83	1.57
(4) PAI	17.34	2.09
(5) PAI	15.75	1.97
(7) PAI	22.94	2.87
(6) PAI	12.13	1.26
Average	15.27	1.90
Standard deviation	4.11	0.54
(1) CU	25.49	3.76
(2) CU	25.33	3.40
(3) CU	24.72	3.74
(4) CU	27.41	3.69
(5) CU	24.11	3.17
Average	25.41	3.55
Standard deviation	1.24	0.26
